# Epidrug screening identifies type I PRMT inhibitors as modulators of lysosomal exocytosis and drug sensitivity in cancers

**DOI:** 10.1038/s41419-025-07900-w

**Published:** 2025-08-08

**Authors:** Baris Sergi, Neslihan Yuksel-Catal, Selahattin Can Ozcan, Hamzah Syed, Umamaheswar Duvvuri, Kirill Kiselyov, Ceyda Acilan

**Affiliations:** 1 Graduate School of Health Sciences, Koc University, Istanbul, Türkiye; 2 Research Center for Translational Medicine (KUTTAM), Koc University, Istanbul, Türkiye; 3 NYU Grossman School of Medicine, NYU Langone Health, New York, NY USA; 4 Department of Biological Sciences, University of Pittsburgh, Pittsburgh, PA USA; 5 School of Medicine, Koc University, Istanbul, Türkiye

**Keywords:** Targeted therapies, Cancer epigenetics

## Abstract

Epigenetic changes drive gene expression alterations, contributing to oncogenesis and drug resistance. Lysosomes play a key role in cell signaling and sequestering toxins, including chemotherapeutic agents, which are then expelled through lysosomal exocytosis—a process linked to drug resistance. However, the epigenetic regulation of lysosomal exocytosis is poorly understood. We hypothesize that epigenetic modifier drugs (epidrugs) inhibiting this exocytosis could serve as potential cancer therapeutics. To explore this, we screened more than 150 epidrugs targeting various epigenetic proteins for their combined cytotoxic effects with cisplatin, their impact on lysosomal exocytosis, and lysosomal biogenesis. Two type I PRMT inhibitors, MS023 and GSK3368715, showed synergy with cisplatin, reduced cell viability, and inhibited lysosomal exocytosis without altering lysosomal biogenesis gene expression. RNA-seq analysis revealed differentially expressed genes involved in vesicular trafficking and lysosome dynamics, suggesting novel regulatory mechanisms. These inhibitors also synergized with other lysosome-sequestered drugs, indicating a broader application in overcoming drug resistance. Analysis of patient data further linked lower type I PRMT levels to better responses, highlighting their potential as combination therapy candidates to enhance chemotherapy efficacy and improve cancer survival rates.

## Introduction

Tumor resistance to chemotherapy is a growing concern, reducing the effectiveness of cytotoxic drugs across various cancer types and treatments. This resistance, whether innate or acquired, presents significant challenges in drug development and cancer therapy, highlighting the need for innovative strategies [1, 2]. Epigenetic regulation drives gene expression changes that promote oncogenic transformation or drug resistance [3, 4]. Small molecule inhibitors of these epigenetic factors (epidrugs) can induce growth arrest, cell differentiation, or apoptosis [5]. To this end, epidrugs offer a promising solution by altering the epigenetic profile of cancer cells, making them more responsive to conventional therapies [1, 2, 6]. Several epigenetic inhibitors have been FDA-approved for treating various cancers, supported by evidence of their efficacy in adult cardiovascular, neurodegenerative, inflammatory, and specific pediatric lysosomal storage diseases [7–13].

One resistance mechanism in cancer cells is the sequestration of chemotherapeutic drugs within lysosomes [14, 15]. Lysosomes, with their high acidity, degrade and absorb nutrients and damaged cellular components and sequester toxins, along with toxic metals [16–19]. This lysosomal sequestration process is documented for many cytotoxic drugs, including platinum compounds like cisplatin, carboplatin, and oxaliplatin [20, 21]. Especially for organic weak base drugs since the acidity of the lysosomal lumen attracts these drugs naturally [22, 23]. Following sequestration, lysosomal exocytosis expels drugs, where lysosomal membranes fuse with the plasma membrane to release contents into the extracellular space, reducing intracellular chemotherapeutic agent concentrations and diminishing their cytotoxic effects [24, 25]. This process is regulated by molecular motors and cytoskeletal elements, involving complex molecular machinery such as SNAREs, trafficking proteins, cytoskeleton, molecular motors, adaptors, and calcium channels, which remove chemotherapeutic agents and modulate the tumor microenvironment, promoting tumor growth and progression [26, 27]. Lysosomal enzyme release during exocytosis degrades extracellular matrix components, promoting cancer cell invasion and metastasis [28–30].

Previous research revealed that blocking lysosomal exocytosis increases the toxicity and intracellular accumulation of transition metals and platinum compounds [31–34]. Thus, manipulating lysosomal flux has the potential to enhance cancer treatments [26, 32, 35, 36]. Understanding the epigenetic regulators of lysosomal exocytosis could develop interventions that potentiate existing chemotherapeutic agents. Research on epigenetic regulation of lysosomal exocytosis is limited. An epigenetic rheostat, including HDAC2 and TFEB, regulates lysosomal and autophagic pathways [37]. HDAC10 depletion sensitizes cells to doxorubicin by inhibiting drug efflux via lysosomal exocytosis [36]. Reduced expression of Sirtuin 1 (SIRT1) inhibits lysosomal function, promoting breast cancer aggressiveness and survival [38]. These studies highlight the relationship between epigenetic modulation and lysosomal dynamics, suggesting targeted epigenetic interventions could disrupt lysosome-mediated drug resistance mechanisms. Our hypothesis focuses on reversing drug resistance by enhancing the cytotoxic activity of chemotherapeutic agents through inhibiting lysosomal exocytosis with epigenetic regulators.

This study reports a novel mechanism of epigenetic regulation impacting lysosomal exocytosis and its implications for cancer therapeutics. We focused on epidrugs due to the increasingly recognized role of epigenetics in cancer biology and the emerging understanding of lysosomal exocytosis in cancer progression and therapy resistance. Using an epigenetic modifier drug library, we screened for drugs that significantly inhibit lysosomal exocytosis, revealing a poorly understood intersection of these two fundamental biological processes.

## Materials and methods

### Cell culture

Du145 (ATCC, HTB-81) and H1299 (ATCC, CRL-5803) cells were maintained in RPMI-1640 (Gibco,11875093), supplemented with 10% FBS (Biowest, S1810) and 1% Pen/Strep (Biowest, L0022) at 37 °C and 5% CO_2_ humidified incubator. Panc1 (ATCC, CRL-1469) and HEK293T (ATCC, CRL-3216) cells were maintained in DMEM (Gibco, 11965118), supplemented with 10% FBS and 1% Pen/Strep at 37 °C and 5% CO_2_ humidified incubator. The rest of the cells were maintained at the ATCC’s recommended medium and culturing conditions. Cells were harvested using 0.05% Trypsin-EDTA (Gibco, 25300-054), passaged, or medium refreshed every 3-4 days, depending on the confluency.

### Epigenetic modifier drug (Epidrug) library screening

The epidrug library containing 175 epigenetic modifier inhibitors was applied (Cayman, 11076) on Du145, Panc1, and H1299 cells. For epidrug and cisplatin combination cell viability screening, 1 × 10^3^ cells were seeded on 96-well plates, and the next day, cells were pre-treated (primed) with epidrugs at a final concentration of 5 µM for 72 h. Following priming, cells were subjected to combinatorial treatment with epidrugs and cisplatin at an indicated concentration for a further 72 h (combination). After treatment incubations were done, screenings proceeded to Sulforhodamine B (SRB) cell viability assay, as indicated in the materials and methods section.

For epidrug library screening with β-Hex lysosomal exocytosis or Lysotracker Green (LTG) staining assays, Du145 cells were seeded on 12-well plates (2 × 10^5^ cell/well) and 96 well plates (4 × 10^3^ cell/well), respectively. The next day, cells were treated with epidrugs (at a constant final concentration of 5 μM) for 72 h. Following epidrug treatment, β-Hex lysosomal exocytosis or Lysotracker Green (LTG) staining assays were performed as indicated in the materials and methods section.

### β-Hex lysosomal exocytosis assay

β-Hex activity was assayed with regular buffer (10 mM HEPES pH 7.4, 150 mM NaCl, 5 mM KCl, 1 mM CaCl_2_, 1 mM MgCl_2_, 2 g/L glucose). 2 × 10^5^ cells were seeded on 12-well plates. The next day, cells were washed once with regular buffer, and cells were incubated with a regular buffer with (stimulated) or without (basal) CuCl_2_ (Sigma, 222011) for 1 h. Buffer on top of cells was collected and further incubated with 3 mM 4-nitrophenyl-N-acetyl-β-D-glucosaminide substrate (Sigma, N9376) for 1 h at 37 °C. The addition of borate buffer stopped reactions, and the absorbance was measured at 405 nm. To determine the total cellular content of β-Hex, cells were lysed with 1% Triton X-100 in PBS, and the cell extract was subjected to the same enzyme activity reaction. Enzyme activity was determined via colorimetric reading as the amount of 4-nitrophenol produced at the end of the reaction, and the activity directly gave the lysosomal exocytosis rate of cells. Absorbance was calibrated with standards made up of 4-nitrophenol (Sigma, N7660) in 0.1 M citrate buffer. To determine the total % of β-hex secreted, the following formula was used: β-Hex secreted, % total = [(Abs_405_ of regular buffer sample)/(Abs_405_ of regular buffer sample + Abs_405_ of lysate sample)].

### LysoTracker Green (LTG) staining assay

To assess changes in lysosomal content following epidrug treatment, LysoTracker Green (LTG) staining was performed. Du145, Panc1, and H1299 cells were seeded in 96-well plates at a density of 4 × 10³ cells/well. The following day, cells were treated with the indicated drugs. At the experimental endpoint, cells were washed once with PBS (Biowest, L0615), and culture medium containing 100 nM LysoTracker Green (Thermo, L7526) was added. Cells were incubated at 37 °C for 1 h in the presence of the dye. After incubation, cells were washed five times with PBS to remove excess dye. Then, fluorescence was measured using a Synergy H1 Hybrid reader (BioTek). Subsequently, cells were lysed with 25 μl of 0.2% SDS, and total protein content was quantified using the BCA Protein Assay Kit (Thermo, 23225). The measured fluorescence values were blank-subtracted, and the resulting relative fluorescence units (RFU) were normalized to the total protein content (μg) to obtain RFU/μg protein.

For flow cytometry analysis, cells were stained with 100 nM LysoTracker Green as described above. Following the 1-h incubation, cells were washed twice with PBS, then detached using 0.25% Trypsin-EDTA and resuspended in PBS containing 2% FBS to maintain cell viability. Samples were analyzed using a Beckman Coulter CytoFLEX flow cytometer, with a minimum of 10,000 events recorded per sample. Median fluorescence intensity (MFI) values were calculated for each sample to assess changes in lysosomal content and normalization to control samples were applied. Population shifts toward increased fluorescence following epidrug treatments were visualized using FlowJo software (version 10.10.0).

### Sulforhodamine B (SRB) cell viability assay

Prior to drug exposure, cells were seeded in 96-well plates at an indicated density. After treatment incubations, the cells were fixed with a final concentration of 10% (w/v) TCA (Sigma, T6399) for 1 h at 4 °C, washed with deionized water, and allowed to air dry at room temperature. 50 μl of 0.4% (w/v) SRB (Santa Cruz, sc-253615) dye was used to stain the cells for 30 min, followed by washing with 1% acetic acid. The SRB dye was dissolved using 150 μl of a 10 mM Tris base solution (Sigma, T1503), and absorbances were measured at 564 nm using a microplate reader (Synergy H1 Hybrid reader, BioTek). Colorimetric measurements were read at 564 nm wavelength. Viabilities were calculated as follows; % Viability = [100 x (Sample Abs-blank)/(Non-treated control Abs-blank)].

### Crystal violet colony formation assay

0.5–2 × 10^3^ cells were seeded on 12-well plates overnight, and then cells were treated, with the medium being refreshed every 72 h after treatments were done. Colony formation was expected to occur within 1–3 weeks. After this period, the medium was aspirated, and the wells were washed with PBS (Biowest, L0616). The cells were then fixed with cold methanol for 15–20 min at −20 °C and then washed twice with PBS. Subsequently, the cells were stained with 0.05% (w/v) crystal violet for 30–60 min, washed with dH2O, and dried, and the stained colonies were visualized. The percentage of formed colonies was analyzed using ImageJ software.

### Calculation of Combination Index (CI)

The “ComBenefit” software was used to calculate the synergy index values for two-drug combinations. SRB cell viability assay was used to obtain the dose-response curves for each drug treatment alone and in combination, and the ComBenefit program calculated the synergistic interactions and CI values by the BLISS scoring model. Effect is calculated by [Effect (*a* + *b*) = *E* (*a*) + *E* (*b*) – *E* (*a*) *E* (*b*)]. The results were interpreted according to the program guidelines. According to the ComBenefit synergy scoring, a negative score indicates antagonism, whereas a positive score indicates synergy. Additionally, increasing absolute scores further potentiates either synergy or antagonism at combined doses of two drugs, stated as an increase of color intensity in surface plots showing cell viability and synergy status.

### Western blotting

Cells were lysed in RIPA buffer (EcoTech, RIPA-100) containing PMSF (Merck, 10837091001) and phosSTOP (Merck, 4906845001) according to the manufacturer’s instructions. Protein concentrations were measured using the BCA Protein Assay Kit (Thermo Fisher, 2322). Proteins were denatured at 95 °C for 10 min in Laemmli buffer (Bio-Rad, 1610747) with DTT (Sigma, D0632) and separated on SDS-PAGE using Mini-PROTEAN TGX Stain-Free Gels (Bio-Rad, 4568086). Following transfer to PVDF (Bio-Rad, 1620177), membranes were blocked in 5% BSA (Sigma, A3733) and incubated overnight at 4 °C with primary antibodies: anti-aDMA (1:5000) (CST, 13522), anti-sDMA (1:2500) (CST, 13222), anti-PRMT1 (1:1000) (Abclonal, A1055), anti-PRMT6 (1:1000) (Abclonal, A5085), anti- FLAG (1:2000) (Thermo, #F3165), and anti-GAPDH (1:5000) (Abcam, AB9485). Membranes were washed with TBS-T, followed by incubation with secondary antibody (Abcam, ab205718) for 1.5 h at room temperature. Proteins were detected using Immobilon Forte western HRP substrate (EMD Millipore, WBLUF0500) and visualized on the Licor Odyssey FC Imaging System.

### Real-time quantitative PCR (RT-qPCR)

Total RNA was isolated using the NucleoSpin RNA II kit (Zymo, R1051) following the manufacturer’s instructions. One microgram of total RNA was reverse transcribed using M-MLV Reverse Transcriptase (Invitrogen, 28025-013) cDNA synthesis kit following the manufacturer’s protocol. RT-qPCR was performed using a commercially available LightCycler 480 SYBR Green I master mix (QIAGEN, 1076717) and the designed primers. The reaction mixture included the master mix, cDNA template, and target gene-specific primers (Supplementary Table 1) in a final volume of 20 μl. Real-time PCR amplification was performed on LightCycler 480 Instrument II (Roche) using the following cycling conditions: initial denaturation at 95 °C for 10 min, followed by 40 cycles of denaturation at 95 °C for 15 s, annealing and extension at 72 °C for 1 min. The target gene expressions were normalized to GAPDH housekeeping gene expression, and fold changes were calculated with the 2^(−ΔΔCt)^ method. To confirm amplification specificity, the PCR products were subjected to a melting curve analysis and subsequent agarose gel electrophoresis if necessary. Statistical analysis was performed using Student’s t-test to compare the expression levels between different groups or conditions with at least three biological replicates.

### Cloning PRMT1 and PRMT6 into pLJC2 lentiviral overexpression vector

To generate FLAG-tagged PRMT1 and PRMT6 overexpression vectors for subsequent ChIP-qPCR experiments using a FLAG antibody, we amplified the coding regions of PRMT1 and PRMT6 from commercially available overexpression vectors (DNASU, #HsCD00950899 for PRMT1 and #HsCD00962806 for PRMT6) using gene-specific primers designed for subcloning (Supplementary Table 2). The amplification was performed with the Phusion DNA Polymerase kit (NEB, #E0553L), following the manufacturer’s instructions. The resulting PCR products were digested with PacI (NEB, #R0547) and NotI (NEB, #R0189) to create compatible sticky ends. Concurrently, the pLJC2-GPT2-3XFLAG vector (Addgene, #163447) was digested with the same enzymes to remove the GPT2 insert and prepare the vector for ligation. Both the digested insert fragments and the vector backbone were separated on a 1% agarose gel, and the desired DNA bands were purified using the NucleoSpin Gel and PCR Clean-up Kit (Macherey-Nagel, #740609.50), as per the manufacturer’s protocol. The purified inserts were then ligated into the prepared pLJC2-3XFLAG vector using T4 DNA Ligase (Thermo Fisher Scientific, #EL0013), following the supplier’s recommended protocol. The ligation mixtures were transformed into *Escherichia coli* Stbl3 competent cells, and transformants were selected on LB agar plates containing the appropriate antibiotic. Positive colonies were screened, and plasmid DNA was isolated using a plasmid DNA purification kit (Macherey-Nagel, #740588.50). The confirmed plasmids were subsequently used to produce viral particles for transduction experiments.

### Generation of PRMT1/6-FLAG overexpressing cells

Early-passage HEK293T cells were seeded at a density of 3.5 × 10⁶ cells per 100 mm dish. The following day, the culture medium was replaced with an antibiotic-free medium. For lentiviral production, a transfection mixture was prepared by combining 2.25 μg psPAX2 (Addgene, #12260), 250 ng VSVG (Addgene, #8454), and 2.5 μg of the plasmid of interest in 200 μl Opti-MEM (Thermo Fisher, #31985062). Separately, 200 μl Opti-MEM was mixed with 15 μl polyethyleneimine (PEI) (Sigma, #408727). The two solutions were combined and incubated at room temperature for 25 min to allow complex formation. The transfection mixture was then vortexed briefly and then added dropwise on top of HEK293T cells and incubated for 18–24 h, after which the medium was replaced with fresh complete medium. At 48 and 72 h post transfection, the conditioned medium containing lentiviral particles was collected and filtered through a 0.45 μm PVDF filter. To concentrate the viral particles, the filtered supernatant was mixed with PEG8000 (Sigma, #25322) and incubated at 4 °C for 24 h. The mixture was then centrifuged at 3000 × *g* for 30 min, and the resulting pellet was resuspended in PBS. The concentrated viral particles were aliquoted and stored at −80 °C until further use.

For viral transduction, Du145, Panc1, and H1299 cells were seeded at a density of 5 × 10⁴ cells per well in a 6-well plate. Then, 30 μl of the concentrated viral suspension was added to each well in the presence of 8 μg/ml protamine sulfate. The next day, the medium was replaced with fresh complete medium, and the cells were allowed to grow for an additional 24 h. At 48 h post transduction, the selection process was initiated by replacing the medium with one containing 2 μg/ml puromycin. The medium was refreshed every 48 h, maintaining puromycin selection until complete death of non-transduced control cells.

### Chromatin Immunoprecipitation qPCR (ChIP-qPCR)

PRMT1/6-FLAG overexpressing Du145, Panc1, and H1299 cells were pelleted as 3 × 10^6^ cells per sample. Subsequently, crosslinking was carried out with 1% formaldehyde (Sigma Aldrich, #818708) at room temperature for 10 min. The reaction was stopped by adding glycine (Sigma Aldrich, #G7126) to a final concentration of 125 mM for 5 min. The cell suspension was then centrifuged at 1000 × *g* for 5 min at 4 °C. After centrifugation, the pellet was washed twice with cold PBS and resuspended in ChIP Lysis Buffer (50 mM HEPES, 1 mM EDTA, 140 mM NaCl, 1% Triton X-100, 0.1% sodium deoxycholate, 1% SDS, 1 mM PMSF, and a Protease Inhibitor Cocktail). The samples were kept on ice for 15 min. Chromatin was subsequently released by sonication using the Bioruptor Plus sonicator (Diagenode), and the resulting lysate was centrifuged at 10,000 × *g* for 10 min at 4 °C. The supernatant containing sheared chromatin fragments (100–500 bp) was collected. To avoid non-specific binding, the chromatin preparation was incubated with prewashed Dynabeads® (Thermo Fisher, Protein G magnetic beads, # 88848) for 30 min at 4 °C (pre-clearing step). Antibodies against anti-mouse FLAG (Thermo, #F3165) and anti-mouse IgG (Sigma Aldrich, # 10400 C) as a control were incubated with magnetic beads in PBST for 1 h at room temperature. Then, the magnetic bead-antibody complexes were combined with the pre-cleared chromatin and incubated overnight. The magnetic beads were then washed successively with ChIP Lysis Buffer, low-salt buffer (0.1% SDS, 1% Triton X-100, 2 mM EDTA, 20 mM Tris-HCl, 140 mM NaCl, pH 8.1), high-salt buffer (0.1% SDS, 1% Triton X-100, 2 mM EDTA, 20 mM Tris-HCl, 500 mM NaCl, pH 8.1), LiCl-containing buffer (0.25 M LiCl, 1% IGEPAL-CA 630, 1% sodium deoxycholate, 1 mM EDTA, 10 mM Tris-HCl, pH 8.1), and finally with Tris-EDTA solution in order, twice for 5 min in each round. DNA elution was carried out using an elution buffer containing 1% SDS (Bio-Rad, #1610418) and 0.1 M NaHCO_3_ (Merck, #S6014). Reverse crosslinking was then performed at 37 °C with RNase A (0.3 mg/ml) (Thermo Fisher Scientific, #12091039) and 5 M NaCl (Merck, #S9888) for 30 min, followed by incubation with proteinase K (0.3 mg/ml) (Thermo Fisher Scientific, #EO0492) at 65 °C for 1 h. DNA was purified using the ChIP DNA Clean & Concentrator kit (Zymo Research, #D5205). ChIP-qPCR primers were designed using UCSC Genome Browser (https://genome.ucsc.edu/) and Ensembl Genome Browser (https://www.ensembl.org/index.html), and their sequences are provided in Supplementary Table 2. Quantitative PCR (qPCR) was performed using the LightCycler 480 system instrument (Roche) to quantify the immunoprecipitated DNA, and the results of the ChIP-qPCR were analyzed using the “% input” method.

### RNA sequencing and analysis

Total RNA was isolated with the Qiagen RNeasy Mini Kit following the manufacturer’s instructions. BGI Group conducted poly-A capture, library preparation, and next-generation sequencing as a contract research service. Sequencing was done with BGIseq, which generated 20 million reads/sample and 150 bp paired-end reads. The sequencing data was stored securely and analyzed on the Koc University high-performance computing cluster (KUACC). The resulting sequencing data were analyzed with a standard RNA-seq pipeline. First, the quality of the reads was assessed using FastQC software. FastQC provided information on the quality score across reads and the per base sequence content and identified adapter contamination. If contamination was present or there were low-quality regions, then TrimGalore was used for trimming. The sequencing data were then mapped to the most recent human reference genome (NCBI GRCh38) with the software HiSAT2. All mitochondrial, mis-spliced genes and duplicates were identified and removed using RSeQC and Picard Tools. Performing this detailed quality control procedure ensured the success of our study in identifying differentially expressed genes. Once the sequencing reads were aligned, the total read counts for each gene were quantified using featureCounts, part of the Subreads package. The results were then analyzed using the R package DESeq2 to identify differentially expressed genes. The significant results from the gene expression analysis were annotated using the biomart database. The final step was performing Gene Set Enrichment Analysis to understand which pathways or gene networks the differentially expressed genes were implicated in. GEO accession number of our RNA-seq analysis is GSE277490.

### Statistical analysis

All data were analyzed in Prism 8 (GraphPad Software, Inc.), and statistical tests were applied as described in the figure legends. Representation of error bars, sample sizes, and *p* values with asterisks were also indicated for each experiment at the figure legends.

## Results

### Cell line panel-based analysis confirms the negative correlation between lysosomal exocytosis and lysosomal volume

Previously published data indicate that chemotherapeutic drugs are sequestered in lysosomes and expelled through lysosomal exocytosis, with the efficiency of this mechanism depending on lysosomal volume and exocytosis rate. Although epigenetic regulation of lysosomal flux is not well understood, the use of epigenetic drug libraries and precise assays presents an opportunity to explore this new translational axis.

**Fig. 1:**
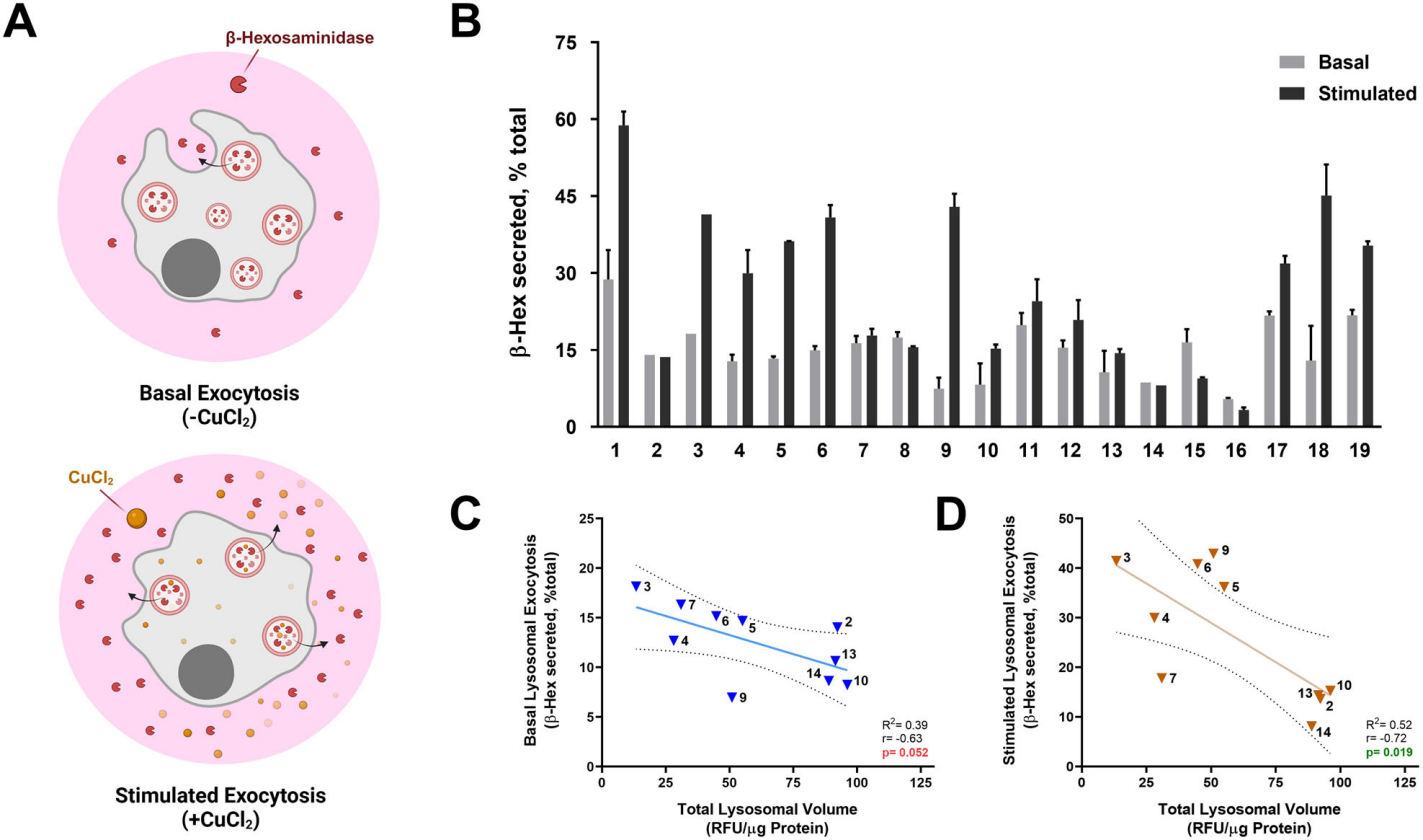
Screening and correlation analysis of lysosomal exocytosis and lysosomal volume across multiple cancer cell lines. **A** Schematic representation of basal and CuCl₂-stimulated lysosomal exocytosis assays (Created with BioRender.com). **B** Lysosomal exocytosis rates (% total of β-hexosaminidase secretion) under basal and CuCl₂-stimulated conditions were measured across a panel of cancer cell lines. Correlation analysis between total lysosomal volume (Lysotracker fluorescence intensity) and lysosomal exocytosis under (**C**) basal and (**D**) stimulated conditions. Each numbered data point represents an individual cell line as follows: **1**. MIA-PaCa-2 (PAAD), **2**. HeLa (CESC), **3**. U2OS (SARC), **4**. U373 (GBM), **5**. PC3 (PRAD), **6**. Du145 (PRAD), **7**. 22Rv1 (PRAD), **8**. LNCaP (PRAD), **9**. H1299 (LUAD), **10**. A549 (LUAD), **11**. HepG2 (LIHC), **12**. Huh7 (LIHC), **13**. MCF-7 (BRCA), **14**. MDA-MB-231 (BRCA), **15**. A2780 (OV), **16**. Kuramochi (HGSOC), **17**. OSCC40 (HNSC), **18**. Panc1 (PAAD), **19**. RPE-1 Cell lines without complete data sets were excluded from correlation plots. Pearson’s correlation (*r*) and *R*² values are indicated on the plots along with corresponding p-values. Shaded areas with dashed line indicate 95% confidence intervals.

To select the optimal cell line for studying lysosomal exocytosis, it was necessary to identify a cell line that yielded satisfactory absorbance values within the linear range for screening changes in exocytosis via β-Hexosaminidase (β-Hex) assay. Additionally, the ideal cell line should exhibit a higher exocytosis rate upon stimulation with copper, which is a common method used to enhance exocytosis by releasing it from lysosomes (Fig. 1A). After evaluating 19 cell lines, Du145 emerged as the most suitable choice due to its superior performance in the exocytosis assay, alongside Panc1, MIA-PaCa-2, H1299 and U373 (Fig. 1B).

To better understand the relationship between lysosomal exocytosis and total lysosomal volume, we hypothesized that inhibiting lysosomal exocytosis would lead to an accumulation of lysosomal content, resulting in increased lysosomal volume. Correlation analysis revealed an inverse relationship between total lysosomal volume and exocytosis, with a modest but near-significant correlation at basal levels (*R*² = 0.39, *p* = 0.052) and a statistically significant correlation under stimulated conditions (*R*² = 0.52, *p* = 0.019) (Fig. 1C, D). Given the biological variability in lysosomal regulation, these findings suggest a consistent association between lysosomal volume and exocytosis, providing a relevant framework for interpreting drug-induced effects.

### Epigenetic drug library screening on Du145 cells identified epidrugs affecting the lysosomal exocytosis, lysosomal volume, and cell viability in combination with cisplatin

To systematically evaluate epidrugs that enhance the efficacy of cisplatin, with a particular focus on those that may operate through lysosomal sequestration and exocytosis, we conducted three separate screens using a drug library comprising 175 small molecules that target diverse epigenetic ‘writer’ (such as Histone Methyl-, Histone Acetyl-, and DNA Methyltransferases), ‘eraser’ (including Histone-Deacetylase and -Demethylases), and ‘reader’ (containing bromodomains) proteins. It is noteworthy that 33 of these compounds are currently undergoing clinical trials, and 13 have been granted FDA approval. Our approach involved measuring the basal and stimulated exocytosis rates of various cell lines.

**Fig. 2:**
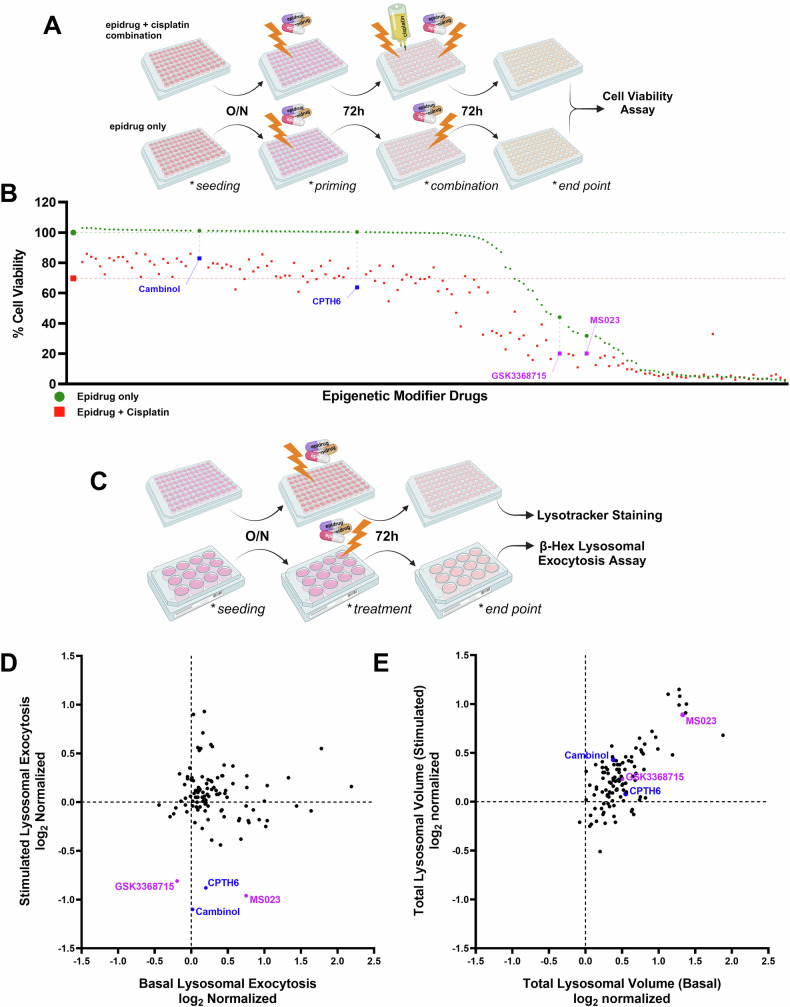
High-content screening identifies epidrugs that synergize with cisplatin and modulate lysosomal exocytosis and lysosomal volume in Du145 cells. **A** Schematic representation of the screening procedure used to identify epidrugs synergizing with cisplatin in a viability assay. Du145 cells were seeded on 96-well plates and pre-treated (primed) with epidrugs (5 µM) for 72 h, followed by combined treatment with epidrugs (5 µM) and cisplatin (5 µM) for an additional 72 h (created with BioRender.com). **B** Scatter plot representing cell viability results from the epidrug library screening, with each dot indicating an individual epidrug. Drugs were classified based on their ability to synergize with cisplatin (red) compared to epidrug-only treatments (green). Key compounds are indicated in blue and purple. **C** Schematic overview of the lysosomal exocytosis and lysosomal volume screening assay (Created with BioRender.com). **D** Screening results showing the effects of epidrugs (5 µM, 72 h) on lysosomal exocytosis activity under basal and stimulated conditions, quantified as extracellular and intracellular β-hexosaminidase activity and represented in log_2_-normalized values. **E** Lysosomal volume changes upon epidrug treatment (5 µM, 72 h), measured by Lysotracker staining. Total lysosomal volume was quantified at basal and stimulated conditions, and values are normalized to the untreated control.

The four cell lines with robust activity were evaluated, and the Du145 cells were selected for further screening since their exocytic activity fell within the linear range in both the basal and stimulated states. To assess cytotoxicity in Du145 cells, a mild dose of cisplatin was utilized, ensuring that approximately 70% of the cells remained viable. Additionally, a consistent dose of epigenetic drugs (5 µM) was administered, as these drugs typically lack cytotoxicity and instead prime cells for subsequent therapies by reprogramming cancer cells to a more normal state. Given the large-scale nature of the epidrug library screening, a single-dose strategy was employed to ensure feasibility. While this approach may limit insights into dose-dependent effects, it provided a practical means to identify potential hits for further investigation. Cells were pre-treated with epigenetic drugs for 72 h and then co-treated with cisplatin for a further 72 h (Fig. 2A). Figure 2B indicates that the majority of tested epigenetic drugs did not significantly impact cell viability (green dots, representing each epidrug aligned on the *x*-axis), suggesting their potential use in sensitizing cancer cells to cisplatin without causing harmful effects on cell viability. A total number of 14 epigenetic drugs that showed decreased cell viability when combined with cisplatin (red dots) were further investigated in cell viability assays with various dose combinations.

A comparable methodology was employed for the follow-up screens, in which cells were pre-treated with 5 µM of epidrugs for 72 h, after which their lysosomal exocytosis and volume were assessed via β-Hex and LysoTracker staining assays, respectively (Fig. 2C). The motivation for examining lysosomal exocytosis and volume assays in both basal and stimulated conditions was rooted in the hypothesis that we would not diminish the basal level of exocytosis in the cells since the exocytosis activity plays a vital role in fundamental cellular processes. We posited that the inhibition of this lysosomal activity, crucial for numerous cellular processes and whose deficiency would result in lethal consequences, could not be accurately replicated in the experimental setup. As shown in Fig. 2D, various drugs showed potent and specific effects on lysosomal flux components. Among these, four epidrugs, type I PRMT inhibitors GS3368715 and MS023, a lysine acetyltransferase inhibitor known as CPTH6, and a SIRT1/2 inhibitor called Cambinol, distinguished themselves for their inhibitory effect on lysosomal exocytosis. These four epidrugs also resulted in a considerable accumulation of lysosomal volume (Fig. 2E), which is consistent with the buildup of lysosomes due to deficiencies in lysosomal exocytosis, as would be expected based on the inverse correlation between exocytosis and lysosome volume in our initial analysis.

To assess whether the findings are cell line-specific or a broader phenomenon, the initial epidrug library screening in Du145 cells was repeated in Panc1 and H1299 cells, followed by cell viability assessment after priming and co-treatment with cisplatin. Consistent with Du145 results, the Type I PRMT inhibitors MS023 and GSK3368715 showed synergy with cisplatin (Supplementary Fig. 1), suggesting a generalizable effect across cell lines.

**Fig. 3:**
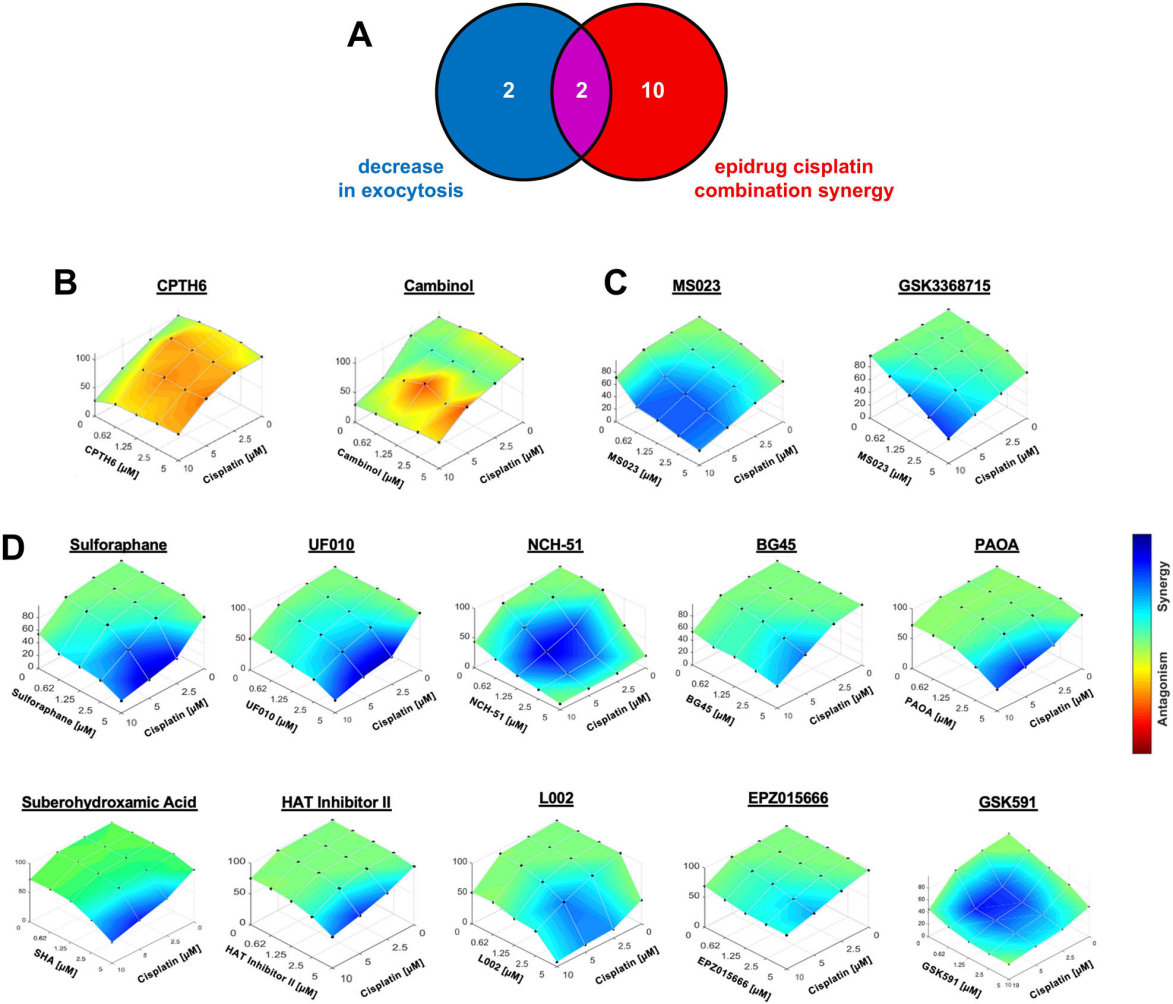
Effect of selected epidrugs from the library on cell viability in combination with cisplatin. **A** Venn diagram summarizing the results of the epidrug library screening performed on Du145 cells, identifying epidrugs that inhibit lysosomal exocytosis and/or show synergistic effects with cisplatin. *Surface plots represent combined drug response (epidrug + cisplatin) in viability assays:* **B** two epidrugs (CPTH6, Cambinol) that decreased lysosomal exocytosis but did not synergize with cisplatin, **C** two epidrugs (MS023, GSK3368715) that both inhibited exocytosis and exhibited synergy with cisplatin, and **D** ten epidrugs that synergized with cisplatin without affecting lysosomal exocytosis. Color gradients indicate the intensity of synergy and antagonism, while cell viabilities are shown on the *y*-axis for combined doses of two drugs.

Based on our findings from three separate screens, we have identified 14 potential epidrugs that may influence lysosomal exocytosis, lysosomal volume, and cisplatin efficacy (Fig. 3A). Through pre-treatment of Du145 cells with these 14 epidrugs in combination with cisplatin at different doses, we discovered that only 2 of the 4 epidrugs that inhibited lysosomal exocytosis also demonstrated synergy with cisplatin (Fig. 3B, C). The remaining 10 epidrugs, although they did not inhibit exocytosis, did synergize with cisplatin (Fig. 3D). In detail, 6 of them are HDAC inhibitors, 2 of them are p300 inhibitors, and the remaining 2 are PRMT5 inhibitors (the synergy scoring for these drugs is presented in detail in Supplementary Fig. 2). Following these screens, we selected the type I PRMT inhibitors “MS023” and “GSK3368715” for further study as they both inhibit exocytosis and demonstrate synergy with cisplatin.

### Type I PRMT inhibitors MS023 and GSK3368715 alter their target cellular processes on Du145, Panc1, and H1299 cells

**Fig. 4:**
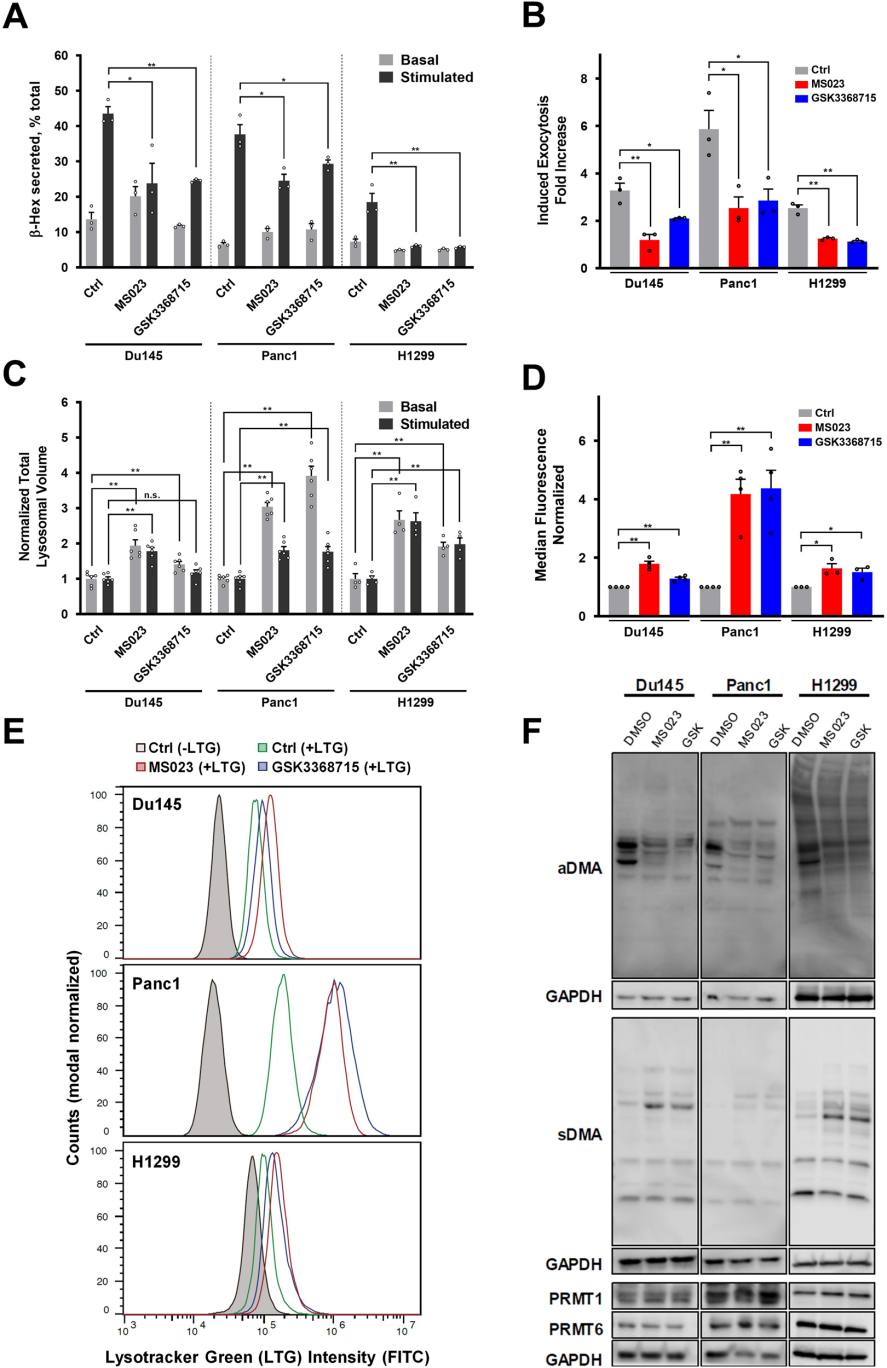
MS023 and GSK3368715 inhibit lysosomal exocytosis, alter lysosomal volume, and reduce arginine methylation in Du145, Panc1, and H1299 cells. Cells were treated with type I PRMT inhibitors MS023 and GSK3368715 (5 μM, 72 h). **A** Lysosomal exocytosis was quantified by measuring β-hexosaminidase secretion at basal and stimulated conditions. **B** Fold induction of exocytosis upon stimulation was calculated (stimulated/basal). Total lysosomal volume after treatment was evaluated using Lysotracker Green staining and quantified by fluorescence intensity measurement (**C**) and flow cytometry analysis (**D**, **E**). Representative flow cytometry histograms are shown in (**E**). **F** Western blot analysis shows the impact of PRMT inhibitors on asymmetric dimethylarginine (aDMA) and symmetric dimethylarginine (sDMA) modifications, as well as the expression levels of PRMT1 and PRMT6 proteins. GAPDH was used as a loading control. Error bars represent the standard deviation from at least three biological replicates (*p < 0.05, **p < 0.01, Student’s *t* test; n.s. not significant).

Protein arginine methyltransferases (PRMTs) are enzymes responsible for the methylation of arginine residues on specific target proteins, a key post-translational modification impacting numerous cellular functions [39]. Type I PRMTs, encompassing PRMT1, -3, -4, -6, and -8, predominantly generate asymmetric dimethylarginine (aDMA) marks on the target protein. This modification plays a significant role in regulating gene expression, signal transduction, RNA processing, and protein-protein interactions [40, 41]. Through the methylation of histones and various nuclear proteins, Type I PRMTs influence chromatin architecture and accessibility, thereby contributing to the epigenetic regulation of gene transcription [39, 42, 43]. Both epidrugs identified as hits in the drug library screens conducted in our study, MS023 and GSK3368715, are defined as drugs that inhibit Type I PRMTs at different inhibition concentrations [44, 45] (Supplementary Table 3). To better understand the properties of these epidrugs, we examined their effects on lysosomal exocytosis, total lysosomal volume, arginine methylation of target proteins, and the expression of lysosomal genes. The epidrugs were tested on three cell lines, Du145, Panc1, and H1299, which were among the most suitable cell lines identified in previous experiments. The β-Hex lysosomal exocytosis assay conducted on three cell lines showed that the treatment with MS023 and GSK3368715 for 72 h inhibited lysosomal exocytosis in the stimulated state (Fig. 4A). Additionally, the ratio of exocytosis at the stimulated and basal conditions (stimulated/basal), which is referred to as induced exocytosis, was reduced post-exposure to epidrugs, indicating that these hit epidrugs alter the way cells release the lysosomal content (Fig. 4B). Moreover, brief exposure to Type I PRMT inhibitors did not affect lysosomal exocytosis rates, confirming that the reduction in exocytosis required transcriptomic priming of the cells rather than being a direct response to the inhibitor (Supplementary Fig. 3).

Lysotracker staining assays demonstrated that Type I PRMT inhibitors led to an increase in the total lysosomal volume in Du145 and Panc1 cells (Fig. 4C). Complementing the fluorescence intensity measurements from Lysotracker staining, flow cytometry experiments showed a rightward shift in the cell population within the treatment groups, further confirming the increase in lysosomal volume (Fig. 4D, E). Given that lysosomal alterations can sometimes be secondary to cytotoxic effects, we assessed the viability of cells treated with these inhibitors. Cell viability assays in all three cell lines revealed that both inhibitors had IC_50_ values exceeding 40 μM at 72 h, far above the 5 μM concentration used in this study (Supplementary Figure 4), indicating that the observed lysosomal changes were unlikely a consequence of acute cytotoxicity.

To assess the impact of the drugs on the stability of type I PRMTs, particularly PRMT1 and PRMT6 -the main targets of MS023 and GSK3368715- we blotted for these proteins. Our findings showed similar levels of protein expression, indicating that the inhibition does not affect protein expression but instead directly influences protein activity (Fig. 4F). Since type I PRMTs mediate asymmetric dimethylation of arginine residues on target proteins, we also analyzed arginine methylation levels to evaluate target specificity and inhibition efficacy. As expected, treatment with both drugs resulted in decreased levels of asymmetric dimethyl arginine (aDMA) and increased levels of symmetric dimethyl arginine (sDMA) as a compensatory mechanism, confirming effective inhibition of the intended targets (Fig. 4F).

Lysosomal biogenesis encompasses not only changes in lysosomal volume but also the expression of lysosomal genes. To investigate this, we examined the expression levels of several lysosomal genes, including the master regulators, TFEB and MiTF. Our panel of lysosomal genes included various lysosomal enzymes, transmembrane proteins, ATP pumps, ABC transporters, and proteins involved in lysosomal exocytosis. After treating Du145, Panc1, and H1299 cells with MS023 and GSK3368715, we observed that the expression of most lysosomal genes in the panel did not change significantly (Supplementary Fig. 5). A more detailed analysis revealed that treatment with these inhibitors affected specific genes involved in endolysosomal function, such as TRPML1 (a key regulator of lysosomal exocytosis and biogenesis encoded by MCOLN1 [46]), TMEM205 (a transmembrane protein associated with cisplatin resistance [47, 48]), and ABCA3 (a lipid transporter involved in lysosomal function [49, 50]). Interestingly, while TRPML1 is part of the CLEAR lysosomal biogenesis network, MS023 did not broadly impact other components of this network, including the structural protein LAMP1, the enzyme palmitoyl-protein thioesterase 1 (PPT1), or genes encoding various cathepsin lysosomal proteases (Supplementary Figure 5). We conclude that type I PRMT inhibitors selectively affect components of the trafficking machinery related to lysosomal exocytosis without broadly disrupting the entire lysosomal biogenesis network.

**Fig. 5:**
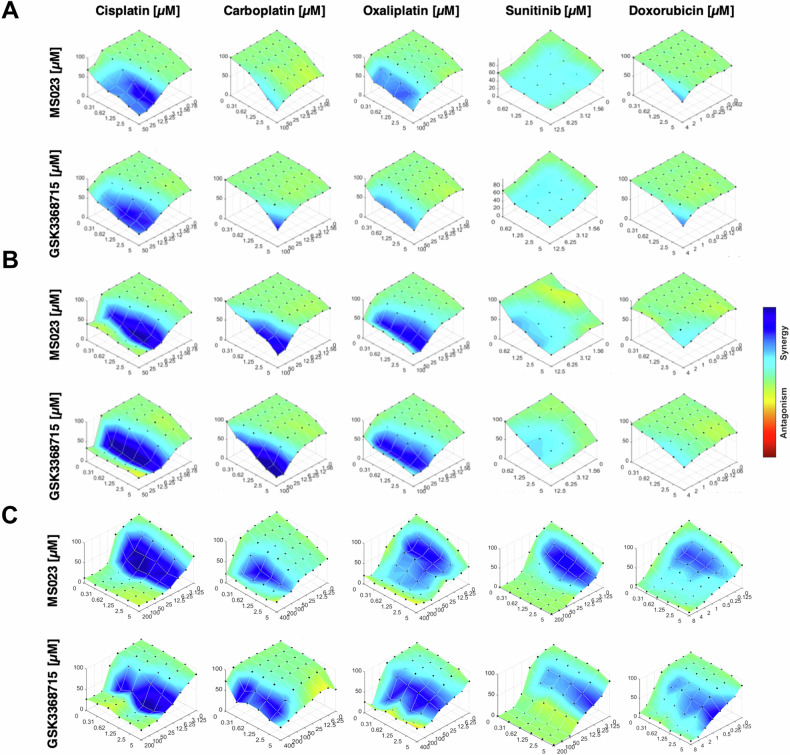
Type I PRMT inhibitors synergize with platinum-based agents and chemotherapeutics known to undergo lysosomal sequestration. Surface plots illustrate the cell viability (y-axis) and synergistic interactions between type I PRMT inhibitors (MS023 and GSK3368715) and various chemotherapeutics (cisplatin, carboplatin, oxaliplatin, sunitinib, and doxorubicin) in (**A**) Du145, **B** Panc1, and **C** H1299 cell lines. Cells were first primed with epidrugs (MS023 or GSK3368715) for 72 h, followed by combined treatment with the indicated drugs at specified concentrations for an additional 72 h. Cell viability was assessed, and synergy analysis was performed using the Bliss model via Combenefit software, where the deepest shade of blue indicates the highest level of synergy.

We further investigated whether epidrugs exhibit synergistic effects with other chemotherapeutic agents, beyond cisplatin, that are known to undergo lysosomal sequestration and exocytosis, which limits their efficacy. Sunitinib, a receptor tyrosine kinase inhibitor, and Doxorubicin, a topoisomerase II inhibitor, are among the drugs that experience lysosomal sequestration [51–54]. We combined the inhibitors MS023 and GSK3368715 with Sunitinib, Doxorubicin, and platinum-based drugs including cisplatin, carboplatin, and oxaliplatin on Du145, Panc1, and H1299 cell lines. The Combenefit analysis demonstrated that both epidrugs exhibited synergistic effects with these chemotherapeutic agents across various concentrations in all cell lines (Fig. 5) (the synergy scoring for these drugs is presented in detail in Supplementary Fig. 6). Notably, similar to the need for reduced exocytosis rates, the synergy between epidrugs and agents like cisplatin and sunitinib was only observed when cells were pre-treated with the epidrugs for 72 h; co-treatment without priming did not show a significant synergy (Supplementary Fig. 7).

The colony formation assay performed on Du145 and Panc1 cells further confirmed this synergy by showing a reduction in the cells’ colony-forming capacity after the combination treatment (Supplementary Fig. 8). These results cumulatively suggest that the inhibition of lysosomal exocytosis by epidrugs enhances the cytotoxicity of the chemotherapeutic agents.

### RNA sequencing analysis on MS023-treated cells reveals differentially expressed genes and enriched gene sets

**Fig. 6:**
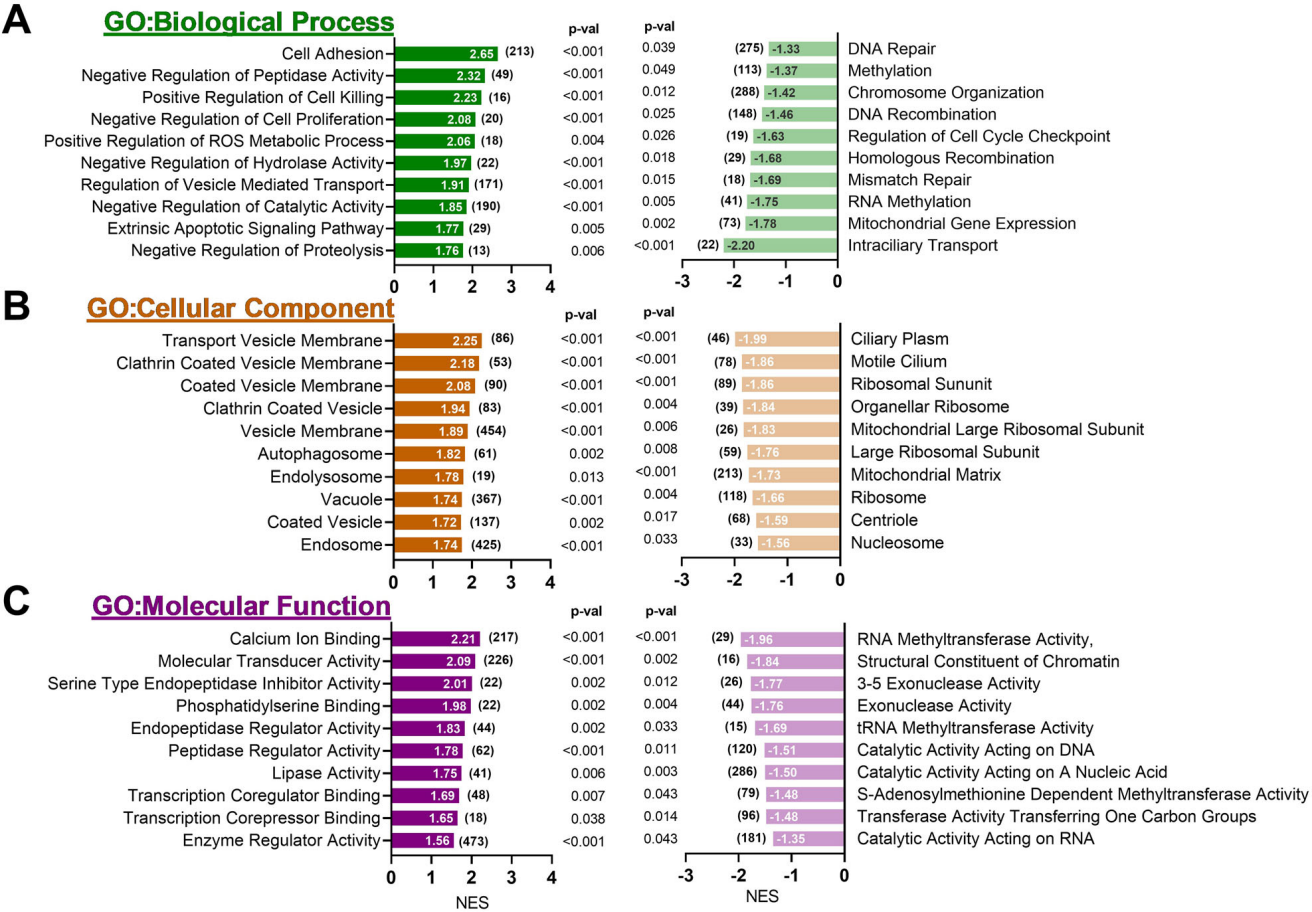
Gene Set Enrichment Analysis (GSEA) identifies enriched pathways upon MS023 treatment in Du145 cells. RNA-seq data from MS023-treated Du145 cells were analyzed using GSEA to identify significantly enriched pathways. Positively (left panel) and negatively (right panel) enriched gene sets are shown, categorized by (**A**) Gene Ontology, Biological Process “c5.go.bp.v2023.1.Hs.symbols.gmt”, (**B)** Gene Ontology, Cellular Components “c5.go.cc.v2023.1.Hs.symbols.gmt”, and (**C)** Gene Ontology, Molecular Function “c5.go.mf.v2023.1.Hs.symbols.gmt” datasets in the MsigDB database.

To explore the synergistic effects of type I PRMT inhibitors with chemotherapy agents and their influence on lysosome volume and exocytosis, we performed RNA-seq analysis on Du145 cells treated with MS023. Initially, the expression changes of the top up- and down-regulated genes within the DEGs were validated through qPCR experiments (Supplementary Fig. 9). Gene ontology analysis confirmed the previously known roles of PRMTs, such as their involvement in DNA repair, methylation, and epigenetic modifications (Fig. 6A), supporting the robustness of our experimental approach. The analysis also revealed the effects of MS023 treatment on lysosomal flux, showing a negative enrichment in genes associated with lysosomal hydrolases, lipases, peptidases, and catalytic activity (Fig. 6A, B). Moreover, GO Molecular Function analysis identified significant changes in cellular components, particularly in vesicular transport, coated vesicles, and vesicle membranes (Fig. 6C).

Notably, there was an enrichment of autophagosomes, endolysosomes, and vacuoles following MS023 treatment, aligning with its impact on lysosomal exocytosis and the observed increase in lysosomal content. The enrichment of coated vesicles, particularly in clathrin-coated vesicle formation (NES scores >2), speculatively suggests increased endocytosis and transport of coated particles from the trans-Golgi network to the lysosomes, contributing to the observed increase in lysosomal content during epidrug treatment experiments.

**Fig. 7:**
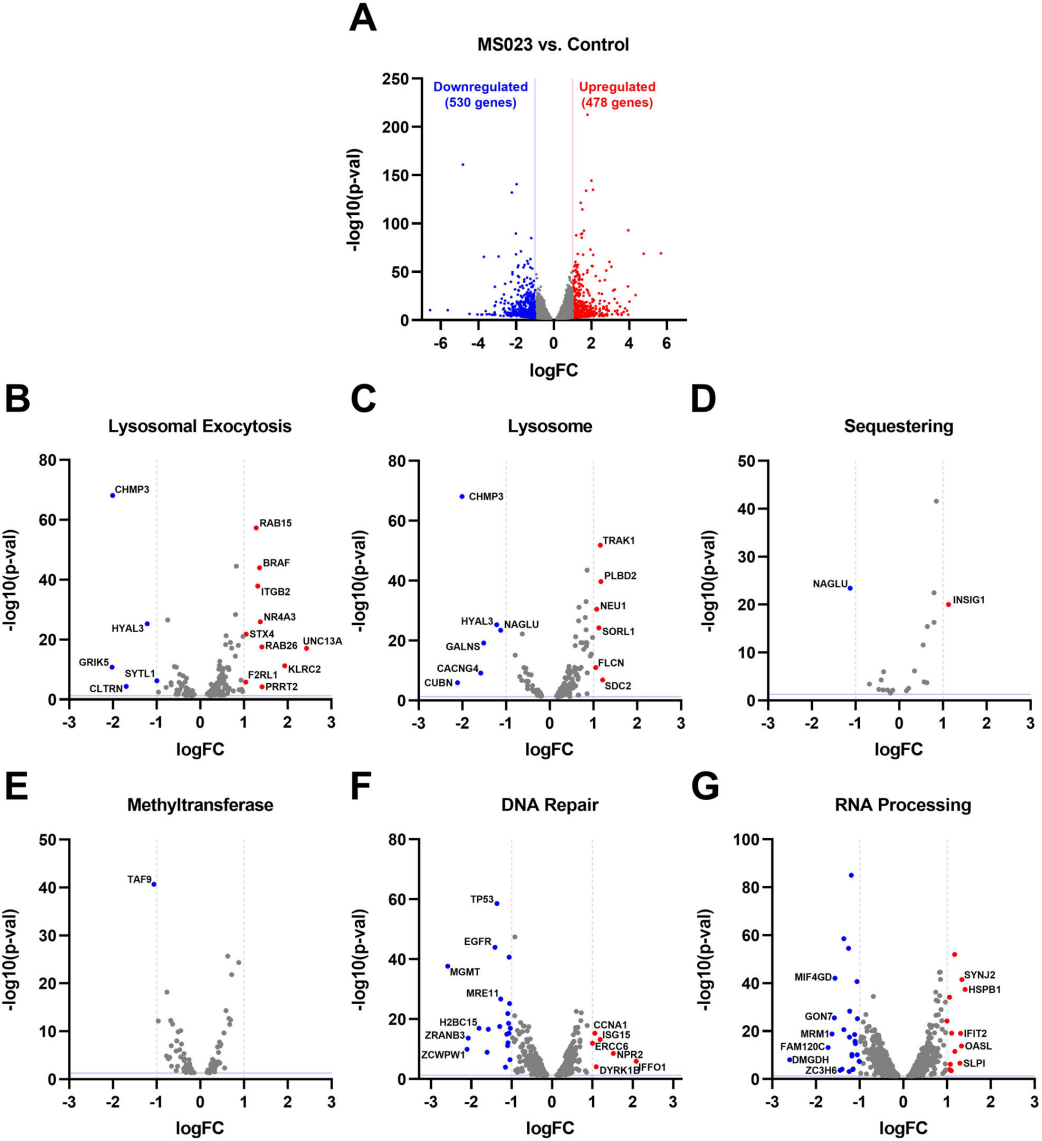
RNA-seq analysis identifies differentially expressed genes within combined gene sets. **A** The volcano plot from the RNA-seq analysis shows the differentially expressed genes following MS023 treatment (*p*-val < 0.05; |log(FC)| > 1). Gene sets from the GSEA Molecular Signature Database were manually selected and categorized into six distinct “combined gene sets” based on their functional relevance (details given in Supplementary Table 4): (**B**) lysosomal exocytosis-related gene sets, (**C**) lysosome-related gene sets, (**D**) sequestration-related gene sets, (**E**) methyltransferase-related gene sets, (**F**) DNA repair-related gene sets, and (**G**) RNA processing-related gene sets. The differentially expressed genes within each of these combined gene sets are marked, and volcano plots were generated, with grey vertical lines indicating the |logFC| > 1 cutoff and the blue horizontal line representing p-val < 0.05.

In addition to GSEA analyses, we examined existing gene sets in the Molecular Signature Database. Based on the intracellular effects of MS023 reported in the literature and its observed impact on lysosomal functions, we generated six main combined gene sets: Exocytosis-, Lysosome-, Sequestering-, Methyltransferase-, DNA Repair-, and RNA Processing-related combined gene sets. Supplementary Table 4 provides a summary of the composition of each combined dataset. We then overlapped the DEGs from our RNA-seq analysis with these combined gene sets to determine which DEGs might be involved in these functions, potentially explaining the reduction in lysosomal exocytosis or the observed drug synergy. Indeed, several genes were identified, and the top up-regulated and down-regulated genes (|logFC| > 1) are highlighted in the volcano plots (Fig. 7).

**Fig. 8:**
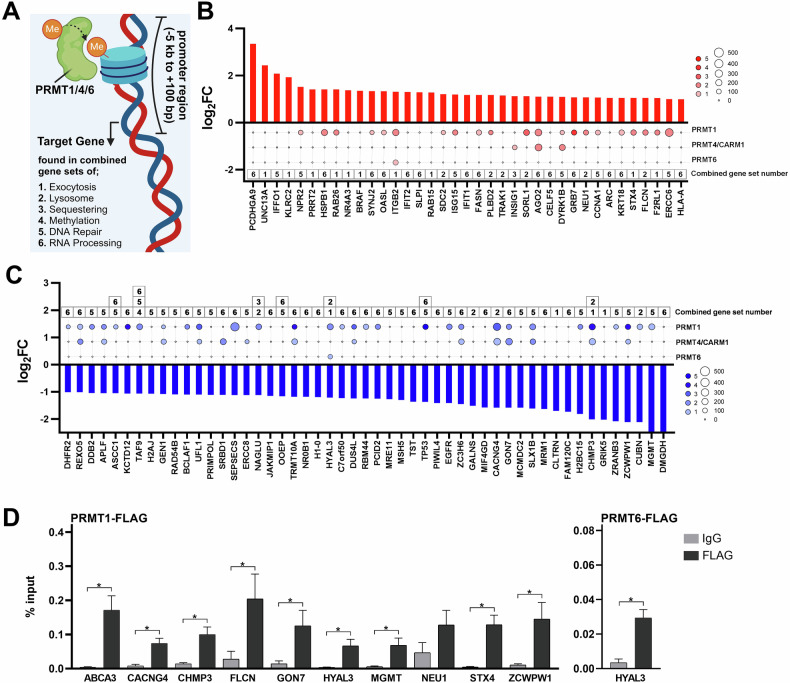
Integrative analysis of RNA-seq and ChIP-seq data reveals type I PRMT occupancy at target gene promoters, validated by ChIP-qPCR. **A** Schematic overview illustrating how promoter regions (–5 kb to +100 bp relative to transcription start sites) of selected target genes from combined gene sets were analyzed for occupancy by PRMT1, PRMT4/CARM1, and PRMT6 using publicly available ChIP-seq datasets from the ChIP-Atlas database (Created with BioRender.com). **B**, **C** Differentially expressed genes identified by RNA-seq (upregulated in red and downregulated in blue) following MS023 treatment were categorized based on their associated combined gene sets: 1. Exocytosis, 2. Lysosome, 3. Sequestering, 4. Methylation, 5. DNA Repair, and 6. RNA Processing. Bar plots indicate logFC expression values, and bubble plots represent PRMT occupancy at promoter regions. Bubble size corresponds to average ChIP-seq enrichment scores, and bubble color intensity reflects the number of ChIP-seq datasets confirming the occupancy peaks. **D** ChIP-qPCR validation confirming PRMT1 and PRMT6 occupancy at selected gene promoters. Du145 cells expressing PRMT6-FLAG or PRMT1-FLAG were used to measure promoter occupancy. Error bars indicate standard error of the mean (SEM) from three independent biological replicates done in duplicate (*p < 0.05, Mann–Whitney *U* test).

To investigate whether type I PRMTs contribute to the regulation of DEGs identified in Fig. 7, we retrieved ChIP-seq data from publicly available datasets on the ChIP Atlas platform (Fig. 8A). Given data availability, we focused on PRMT1, PRMT4, and PRMT6 (based on data availability only for those Type I PRMTs on ChIP-Atlas platform) occupancy at the promoter regions of genes exhibiting significant up- or downregulation in our RNA-seq data (|logFC| > 1) (Fig. 8B, C). This analysis provided insights into PRMT-mediated methyltransferase activity and its potential role in transcriptional regulation.

To further validate these findings, we conducted ChIP-qPCR experiments in Du145 cells to assess PRMT1 and PRMT6 occupancy at the promoter regions of selected DEGs. PRMT1 and PRMT6 were cloned into FLAG-tagged lentiviral vectors to generate stable overexpression models, which were confirmed via Western blot (Supplementary Fig. 10). ChIP-qPCR using gene-specific primers (Supplementary Table 2) demonstrated PRMT binding at the promoter regions of most selected genes (Fig. 8D), in agreement with the public dataset analysis. Similar ChIP-qPCR experiments on Panc1 and H1299 cells confirmed PRMT occupancy at the promoter regions of key DEGs in these cell lines as well (Supplementary Fig. 11). These observations strongly support the hypothesis that type I PRMTs directly regulate the expression of specific genes involved in lysosomal functions and related pathways, highlighting the potential of type I PRMT inhibitors to modulate epigenetic marks and alter gene expression, ultimately impacting cellular processes such as lysosomal exocytosis.

To further validate our findings and assess the clinical relevance of PRMT-1, -4, -6, and -8 in chemotherapy resistance, we analyzed patient data from the Cancer Treatment Response Gene Signature Database (CTR-DB), a publicly available resource. This analysis aimed to determine whether the expression levels of type I PRMTs could serve as predictive biomarkers for chemotherapy response, particularly for treatments known to involve lysosomal sequestration. We specifically compared the expression levels of these PRMTs between patients who responded to therapy and those who did not, to establish a connection between our in vitro results and actual patient outcomes.

**Fig. 9:**
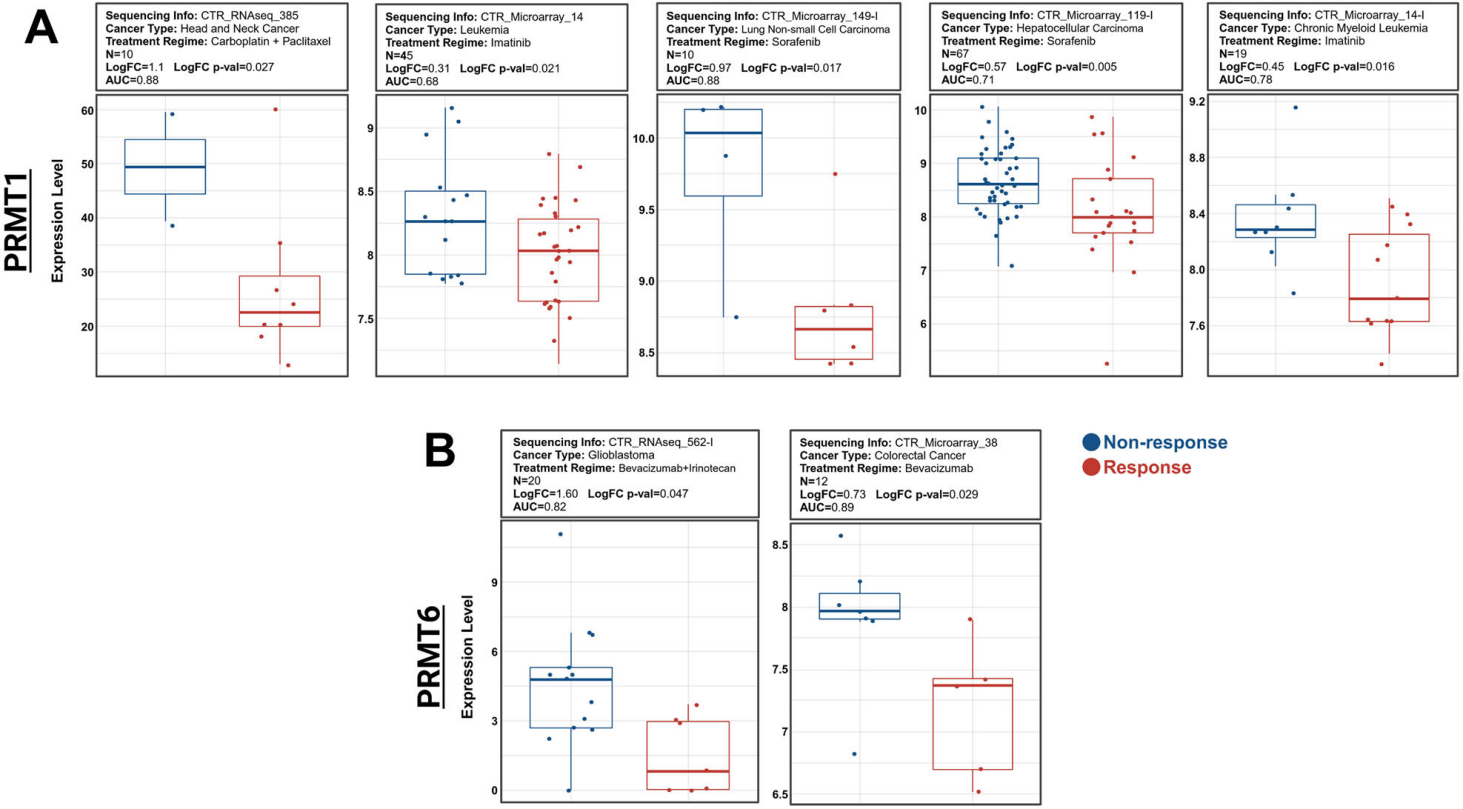
Correlation of PRMT1 and PRMT6 expression levels with patient response to chemotherapy. Expression levels of (**A**) PRMT1 and (**B)** PRMT6 in patients classified as responsive (red) or non-responsive (blue) to various chemotherapy regimens. Patient data were retrieved from the Cancer Treatment Response Gene Signature Database (CTR-DB). Box plots illustrate differences in PRMT1 or PRMT6 expression levels between the two groups, indicating generally higher PRMT expression in non-responsive patients. The specific cancer types, chemotherapy regimens, sample numbers (N), fold-change values (LogFC), statistical significance (*p*-values), and area under the curve (AUC) values are indicated for each comparison.

The patient cohort consisted of individuals diagnosed with a specific type of cancer, divided into two groups based on their response to standard chemotherapy. Responders were defined as patients who exhibited a significant reduction in tumor size or disease stabilization, while non-responders showed no improvement or disease progression despite treatment during treatment [55]. Using this classification, we focused on the patient groups who take specific chemotherapy drugs which attributed to the lysosomal sequestration and exocytosis events, including cisplatin [34, 56], carboplatin [57], Imatinib [58, 59], Sorafenib [60], and Bevacizumab [61]. Our analysis did not find any significant association between PRMT-3, -4, or -8 expression and chemotherapy resistance. However, we observed that PRMT1 and PRMT6 expression levels were significantly higher in the non-responder group compared to the responder group under the specified treatment regimens, consistent with our hypothesis (Fig. 9). The correlation between low PRMT1 and PRMT6 expression and better therapeutic response reinforces our in vitro findings, supporting the idea that inhibiting type I PRMTs can reduce lysosomal exocytosis-mediated drug tolerance and enhance chemotherapy efficacy.

## Discussion

This study aimed to explore the intersection of epigenetic regulation and lysosomal exocytosis in cancer therapy, particularly in overcoming chemotherapeutic resistance. Our findings underscore the significance of lysosomes in sequestering and expelling chemotherapeutic drugs, a process that contributes to drug resistance. By targeting the epigenetic regulation of lysosomal exocytosis, we sought to enhance the therapeutic efficacy of chemotherapy.

We implemented a comprehensive screening process that utilized an epigenetic drug library in order to pinpoint epidrugs that modulate lysosomal exocytosis. The identification of specific epidrugs that inhibit lysosomal exocytosis without broadly affecting lysosomal biogenesis genes underscores the specificity and clinical utility of our findings. We narrowed our analysis to two epidrugs, MS023 and GSK3368715, which target the same epigenetic modifiers, type I PRMTs, emerging as potential molecules that synergize with cisplatin while reducing exocytosis. Notably, the efficacy of the drug combination varied depending on the chemotherapeutic agent used. RNA-seq analysis revealed that these epidrugs affected genes involved in several pathways, including lysosomal function and DNA repair. This suggests that drugs like cisplatin, which cause DNA damage and are sequestered to lysosomes, can synergize through both mechanisms, while Sunitinib, acting through a different pathway, shows less synergy, potentially due to its reliance solely on reduced exocytosis. The observed synergy highlights the potential of using epigenetic drugs to enhance the effectiveness of DNA-damaging agents in cancer therapy by simultaneously targeting multiple pathways, including lysosomal exocytosis.

Type I PRMTs regulate critical cellular processes, including DNA damage response, repair, RNA processing, and splicing [62, 63]. PRMT1 and PRMT6 influence DNA repair by methylating key proteins [64, 65], while PRMT4 modulates RNA splicing under stress [66]. PRMT8, primarily studied in the nervous system, is involved in RNA processing [67]. Targeting these enzymes with inhibitors is a promising therapeutic strategy, particularly in cancer. Inhibition of PRMT1 in triple-negative breast cancer (TNBC) cells triggers an interferon response, leading to cell death [68], and disrupts chromatin recruitment, enhancing cisplatin efficacy [69]. Our results indicate that, in addition to the previously discussed mechanisms, Type I PRMT inhibitors decrease lysosomal exocytosis—a novel finding that, to our knowledge, is being described for the first time. This discovery further enhances the potential of these inhibitors in improving chemotherapy efficacy.

Our RNA-seq analysis suggested potential PRMT-mediated regulation of lysosomal genes. Although publicly available ChIP-seq datasets provided initial insights, they had limitations, as they originated from different cell lines than those used in our study. To address this, we performed ChIP-qPCR experiments, confirming PRMT1 and PRMT6 occupancy at the promoters of several key genes identified in our RNA-seq analysis. These results support the direct regulation of these genes by type I PRMTs. Moreover, the correlation between low PRMT1 and PRMT6 expression and better therapy response in patient data underscores the relevance of our in vitro results. This supports the idea that type I PRMT inhibitors can mitigate lysosomal exocytosis-mediated drug resistance, potentially enhancing the effectiveness of chemotherapeutic agents, as shown in our cell line studies. While this correlation underscores the potential role of PRMT1 and PRMT6 in influencing chemotherapy response, we recognize that these data alone are not sufficient to establish those PRMTs as definitive predictive biomarkers. Further investigation is warranted to validate these observations and determine their potential clinical relevance.

The discovery of MS023 and GSK3368715 as promising candidates for combination therapy, identified through a comprehensive epigenetic drug library, opens new avenues for epigenetic drug research in cancer treatment. However, several key questions remain. The precise mechanisms by which these and other epigenetic drugs influence lysosomal exocytosis and biogenesis need further exploration. In this study, we focused on the TRPML1/TFEB axis as the paradigmal lysosomal regulation pathway. As discussed above, inhibition of type I PRMTs only partially affects this pathway, suggesting the involvement of other regulatory circuits. In line with this, other pathways, including RAS-MAPK/ERK and AMPK, appear to regulate the lysosomal system [70, 71]. Our gene expression analysis revealed differential regulation of key lysosomal hydrolases that influence lysosomal exocytosis and cell signaling. NEU1, a known negative regulator of lysosomal exocytosis [72], was upregulated, while NAGLU and GALNS, involved in glycosaminoglycan degradation [73–75], were downregulated. The resulting accumulation of glycosaminoglycans may disrupt cellular homeostasis and impact signaling pathways. These findings suggest that type I PRMT inhibition may indirectly affect the EGFR-RAS-MAPK signaling axis via lysosomal hydrolase activity, ultimately influencing lysosomal exocytosis and drug sensitivity in cancer cells.

Future investigations should clarify whether PRMT1 inhibition counteracts drug-induced lysosomal exocytosis through MAPK-dependent mechanisms and how these pathways interact with epigenetic regulation. Additionally, determining the cancer types most susceptible to this modulation will be essential for translating these findings into therapeutic strategies. Overall, our results establish a mechanistic framework for targeting lysosomal exocytosis through epigenetic interventions as a means to enhance drug sensitivity in cancer cells.

## Supplementary information

Merged Supplemental Materials

## Data Availability

The RNA-seq data generated and analyzed in this study are publicly available in the Gene Expression Omnibus (GEO) under the accession number GSE277490. No other datasets were generated during the current study.
