# Supplementary material for: Epidrug screening identifies type I PRMT inhibitors as modulators of lysosomal exocytosis and drug sensitivity in cancers
Source: Cell Death Dis. 2025 Aug 8;16(1):600. doi: 10.1038/s41419-025-07900-w (PMC12334744; doi:10.1038/s41419-025-07900-w)
Supplement: Supplementary file 1 — Merged Supplemental Materials [file 41419_2025_7900_MOESM1_ESM.pdf]

Supplemental Material: Legends of Supplementary Figures and Tables

The following supplementary material section includes legends of supplementary figures and tables of this study

Legends of Supplementary Figures and Tables

Supplementary Figure 1. Epidrug library screening identifies epidrugs that synergize with cisplatin on Panc1 and H1299 cells. (**A**) Panc1 and (**B**) H1299 cells were seeded on 96-well plates and pre-treated (primed) with the epidrugs (at a final concentration of 5 μM) for 72h. Panc1 cells were then exposed to 5 μM epidrug and 5 μM cisplatin combination for another 72h, whereas H1299 cells were exposed to 5 μM epidrug and 25 μM cisplatin combination for 72h. SRB cell viability test was conducted at the end of the treatment. Viabilities were normalized to untreated condition and expressed as % cell viability. Red dashed line corresponds to % cell viability when cisplatin alone is used, whereas green dashed line corresponds to the non-treated % cell viability (normalized, 100%). Two Type I PRMT inhibitor hits (MS023 and GSK3368715) are highlighted as purple color on the graph.

Supplementary Figure 2. Synergy scores of combinatorial treatments involving hit epidrugs and cisplatin indicate synergy on Du145 cells. Cell viability assay results from combination treatments of the 14 picked epidrugs from the library cell viability screening with cisplatin on Du145 cells were analyzed using the Combenefit software employing the BLISS model. Synergy status of each drug combinations is indicated in the synergy score matrix.

Supplementary Figure 3. Type I PRMT inhibitors MS023 and GSK3368715 do not inhibit the lysosomal exocytosis upon brief exposure. Du145, Panc1, and H1299 cells were briefly exposed (1h) to MS023 and GSK3368715 epidrugs, then subjected to β-Hex lysosomal exocytosis assay. Error bars represent the standard deviation of at least three biological replicates (Student's t-test, *$p<0.05$, n.s.=not significant).

Supplementary Figure 4. Type I PRMT inhibitors MS023 and GSK3368715 show minimal to no cytotoxicity at high doses on Du145, Panc1, and H1299 cells. Cells were treated with (**A**) MS023 and (**B**) GSK3368715 at indicated doses for 72 h. Cell viability was determined via SRB assay. Error bars represent the standard deviation of 3 biological replicates.

Supplementary Figure 5. Type I PRMT inhibitors, MS023 and GSK3368715, do not cause an overall expression change in lysosomal gene network. Expressions of genes in the lysosomal gene panel were quantified after MS023 and GSK3368715 treatments (5 μM, 72 h) in (**A**) Du145, (**B**) Panc1, and (**C**) H1299 cells. Error bars represent the standard deviation of three biological replicates (Student's t-test; *$p<0.05$).

Supplementary Figure 6. Synergy scores of combinatorial treatments involving Type I PRMT inhibitors and chemotherapeutic agents indicate synergy on cancer cells. Cell viability assay results from combination treatments of the Type I PRMT inhibitors MS023 and GSK3368715 with chemotherapeutic agents in (**A**) Du145, (**B**) Panc1, and (**C**) H1299 cells were analyzed using the Combenefit software employing the BLISS model. Synergy status of each drug combinations is indicated in the synergy score matrix.

Supplementary Figure 7. Type I PRMT inhibitors MS023 and GSK3368715 do not synergize with chemotherapeutic agents without priming. Epidrugs were only co-treated with cisplatin or sunitinib for 72 h without priming on (**A**) Du145, (**B**) Panc1, and (**C**) H1299 cells. Epidrug and cisplatin/sunitinib combination doses were shown at the horizontal axis, whereas % cell viability values were plotted at the vertical axis. Calculated synergy scores are indicated below as a matrix.

Supplementary Figure 8. Effect of synergy between Type I PRMT inhibitors and chemotherapeutic agents on colony formation capacity of cells. Colony formation assay performed on Du145 and Panc1 cells confirmed the synergistic effect through inhibition of colony forming capacity of cells after the combination of each epidrug with chemotherapeutic agents. Du145 cells and Panc1 cells were primed with epidrugs MS023 and GSK3368715 for 72 h, then combination treatments were carried out with epidrugs and indicated chemotherapeutic agents for 72 h.

Supplementary Figure 9. Validation of mRNA expression change of top selected up- and down-regulated genes. Top (**A**) up- and (**B**) down-regulated genes were picked, and expression changes were validated via RT-qPCR. Error bars represent the standard deviation of three biological replicates (Student's t-test; *$p<0.05$).

Supplementary Figure 10. Western blot experiments confirm the stable PRMT1/6-FLAG overexpression on Du145, Panc1, and H1299 cells. PRMT1 and PRMT6 constructs were cloned into pLJC2-3xFLAG lentiviral overexpression backbone and stable overexpression was validated via western blotting experiments using anti-FLAG, anti-PRMT1, and anti-PRMT6 antibodies. GAPDH was used as a loading control.

Supplementary Figure 11. ChIP-qPCR analysis confirm that promoter regions of selected genes found in combined gene sets are occupied by PRMT1 and PRMT6. PRMT1 and PRMT6 ChIP-qPCR experiments performed on PRMT1/6-FLAG overexpressing (**A**) Panc1 and (**B**) H1299 cells confirm that promoter regions of selected target genes are occupied by PRMT1 and PRMT6. Error bars indicate standard error of the mean (SEM) from three independent biological replicates done in duplicate (*$p<0.05$, Mann–Whitney U test).

Supplementary Table 1. Sequence information of primers used in the RT-qPCR experiments.

Supplementary Table 2. Primers used in the ChIP-qPCR experiments and PRMT1/6 cloning into pLJC2 lentiviral overexpression vector.

Supplementary Table 3. Information about type I PRMT inhibitors and their inhibition IC50 values on target PRMTs

Supplementary Table 4. The list of gene sets being used to curate combined gene sets for RNA-seq analysis

Supplemental Material: Supplementary Figures and Tables

The following supplementary material section includes supplementary figures and tables of this study

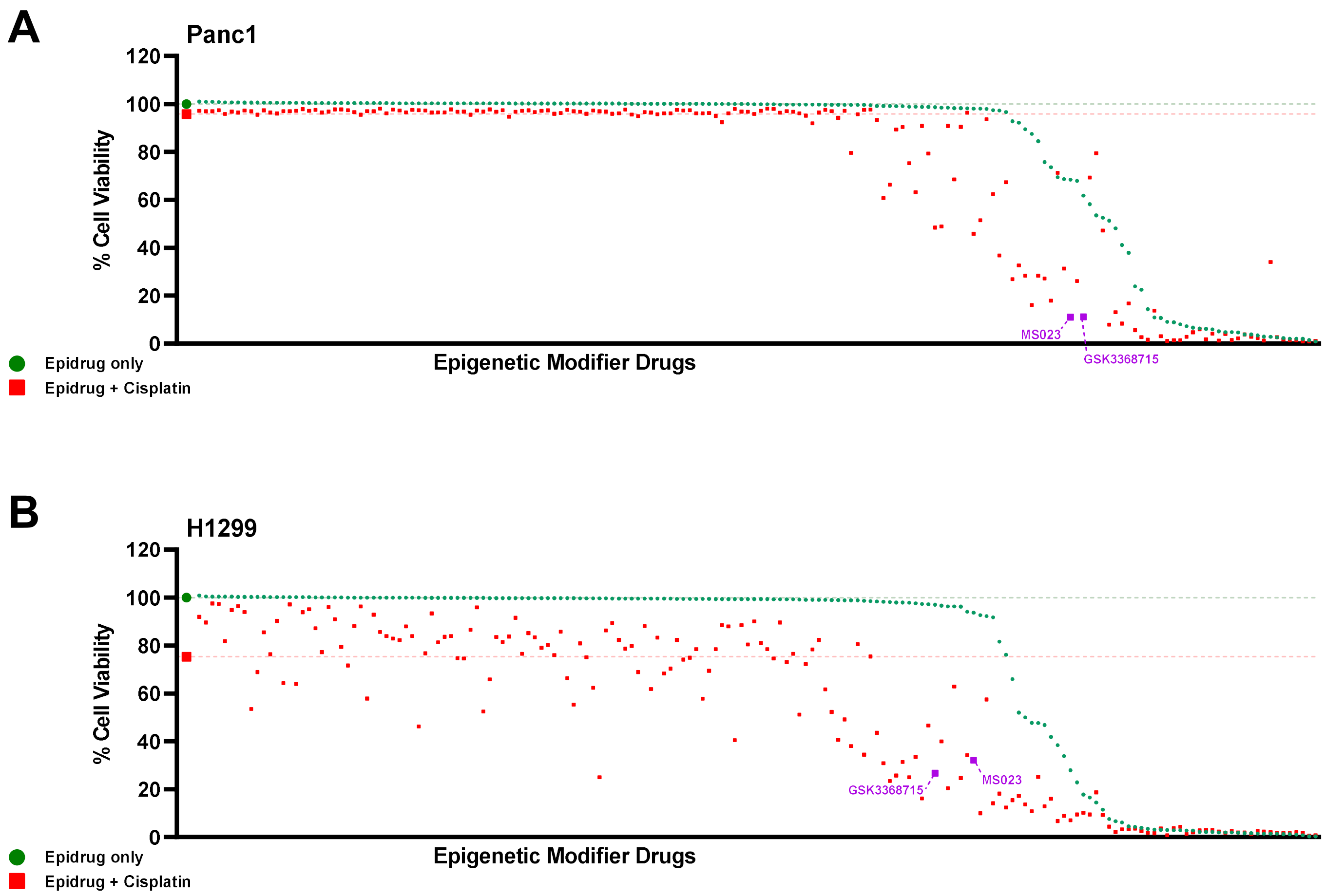

Supplementary Figure 1. Epidrug library screening identifies epidrugs that synergize with cisplatin on Panc1 and H1299 cells. (**A**) Panc1 and (**B**) H1299 cells were seeded on 96-well plates and pre-treated (primed) with the epidrugs (at a final concentration of 5 μM) for 72h. Panc1 cells were then exposed to 5 μM epidrug and 5 μM cisplatin combination for another 72h, whereas H1299 cells were exposed to 5 μM epidrug and 25 μM cisplatin combination for 72h. SRB cell viability test was conducted at the end of the treatment. Viabilities were normalized to untreated condition and expressed as % cell viability. Red dashed line corresponds to % cell viability when cisplatin alone is used, whereas green dashed line corresponds to the non-treated % cell viability (normalized, 100%). Two Type I PRMT inhibitor hits (MS023 and GSK3368715) are highlighted as purple color on the graph.

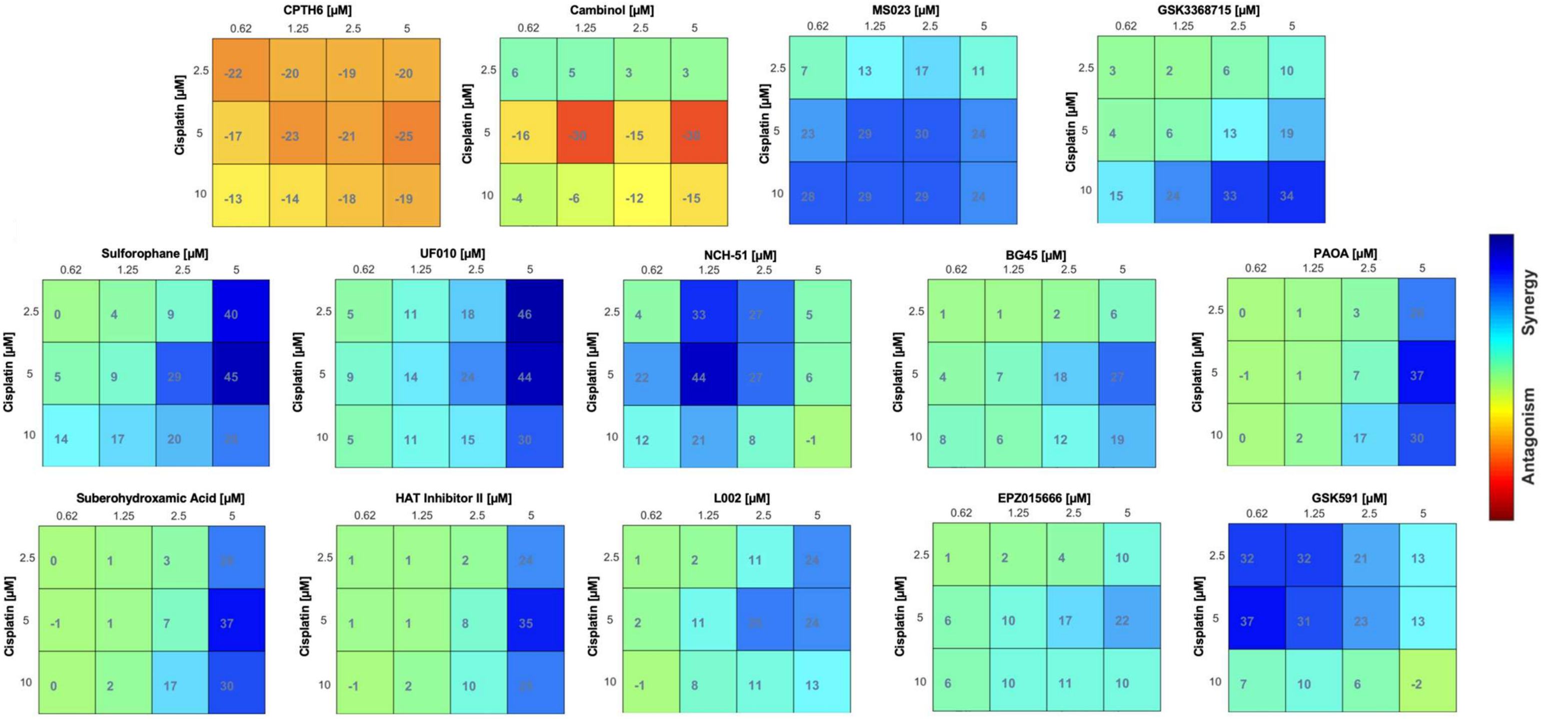

Supplementary Figure 2. Synergy scores of combinatorial treatments involving hit epidrugs and cisplatin indicate synergy on Du145 cells. Cell viability assay results from combination treatments of the 14 picked epidrugs from the library cell viability screening with cisplatin on Du145 cells were analyzed using the Combenefit software employing the BLISS model. Synergy status of each drug combinations is indicated in the synergy score matrix.

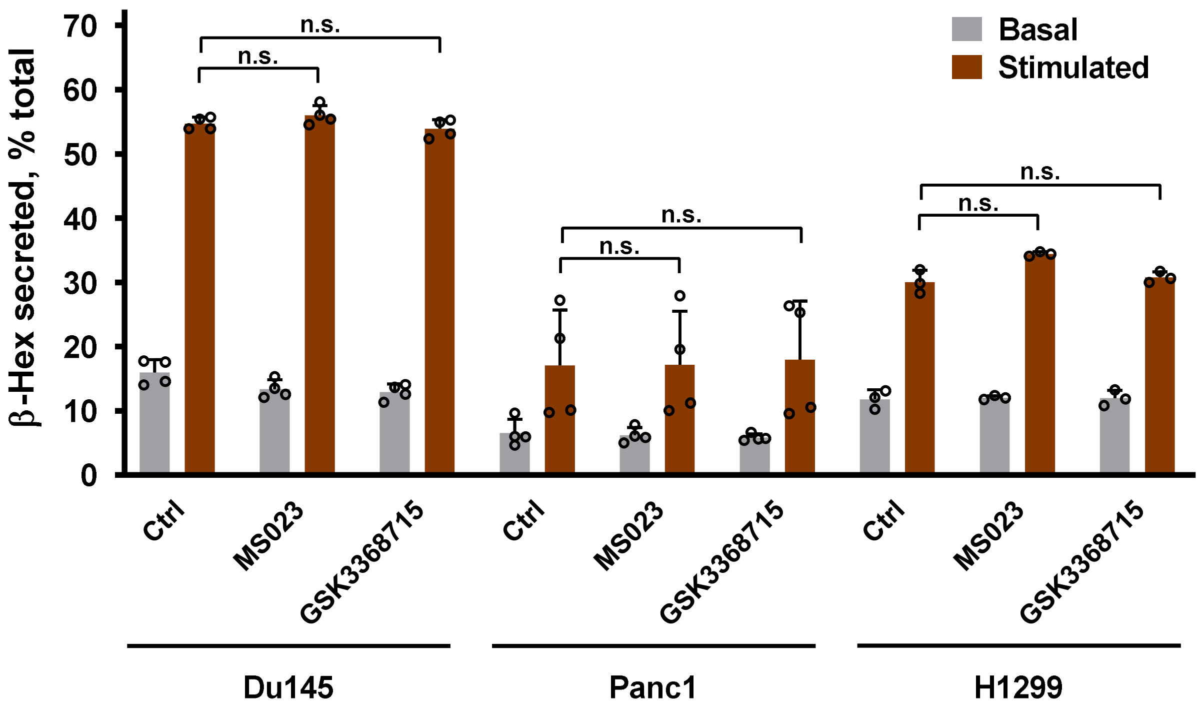

Supplementary Figure 3. Type I PRMT inhibitors MS023 and GSK3368715 do not inhibit the lysosomal exocytosis upon brief exposure. Du145, Panc1, and H1299 cells were briefly exposed (1h) to MS023 and GSK3368715 epidrugs, then subjected to β-Hex lysosomal exocytosis assay. Error bars represent the standard deviation of at least three biological replicates (Student's t-test, *p<0.05, n.s.=not significant).

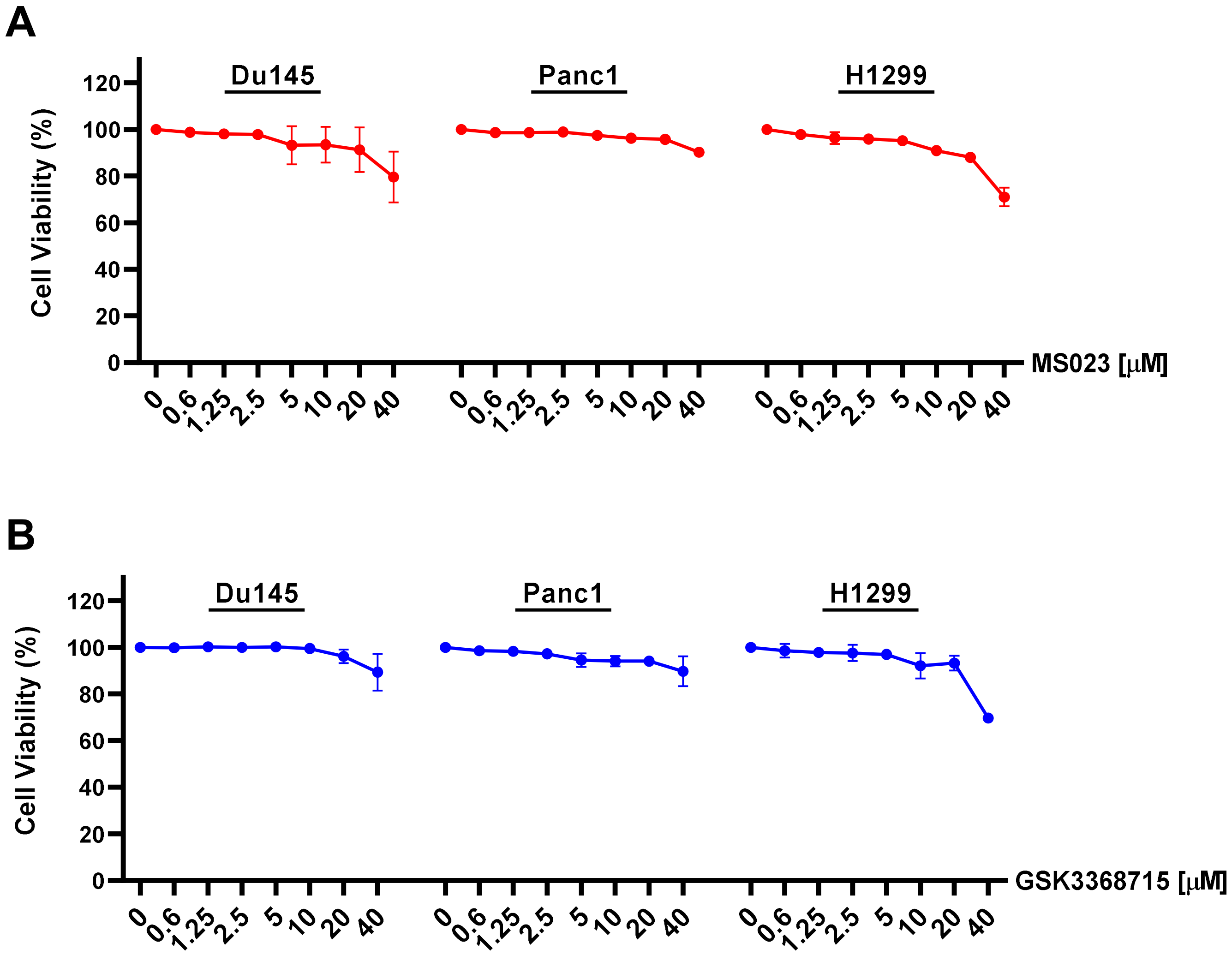

Supplementary Figure 4. Type I PRMT inhibitors MS023 and GSK3368715 show minimal to no cytotoxicity at high doses on Du145, Panc1, and H1299 cells. Cells were treated with (**A**) MS023 and (**B**) GSK3368715 at indicated doses for 72 h. Cell viability was determined via SRB assay. Error bars represent the standard deviation of 3 biological replicates.

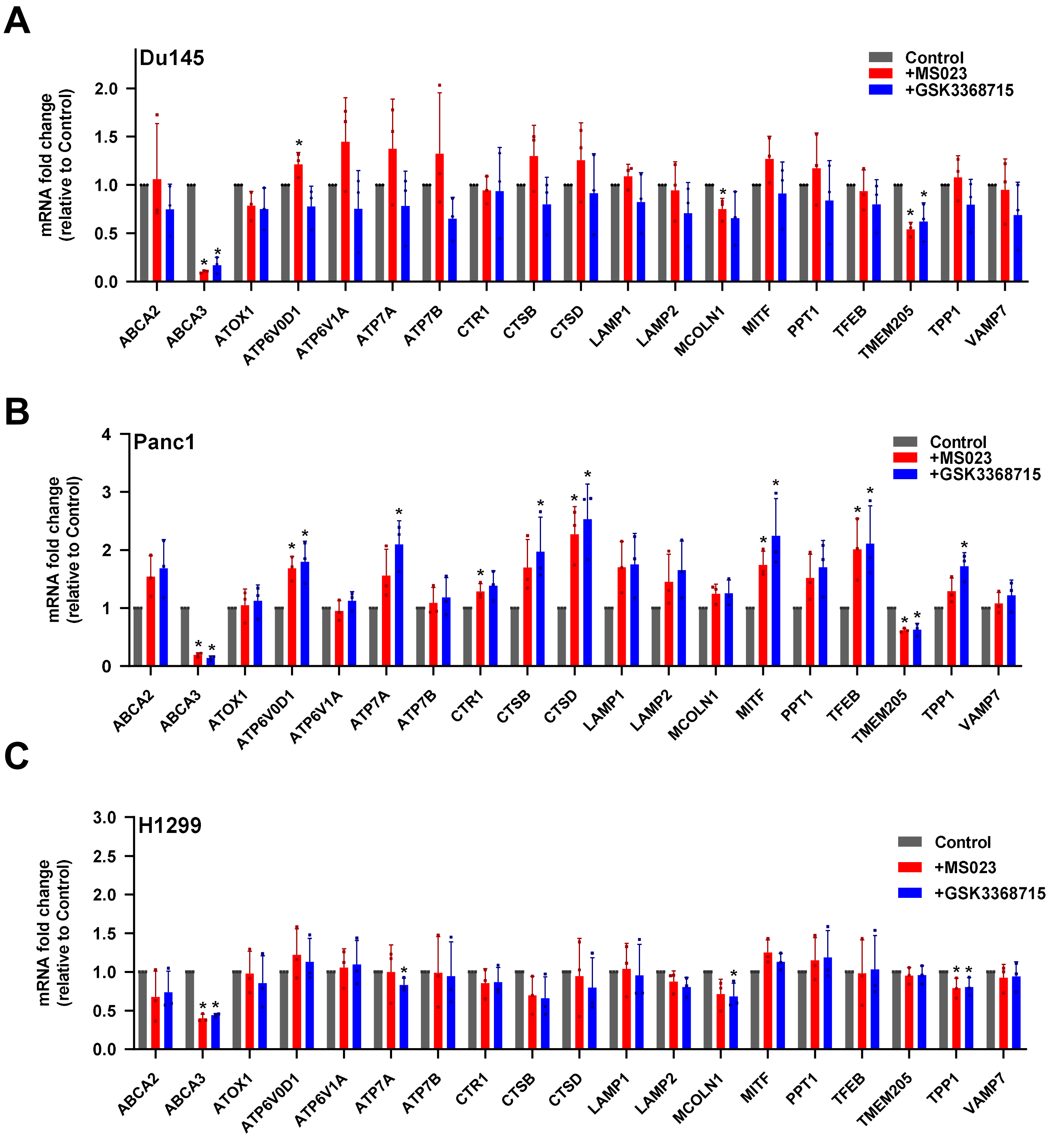

Supplementary Figure 5. Type I PRMT inhibitors, MS023 and GSK3368715, do not cause an overall expression change in lysosomal gene network. Expressions of genes in the lysosomal gene panel were quantified after MS023 and GSK3368715 treatments (5 μM, 72 h) in (**A**) Du145, (**B**) Panc1, and (**C**) H1299 cells. Error bars represent the standard deviation of three biological replicates (Student's t-test; *p<0.05).

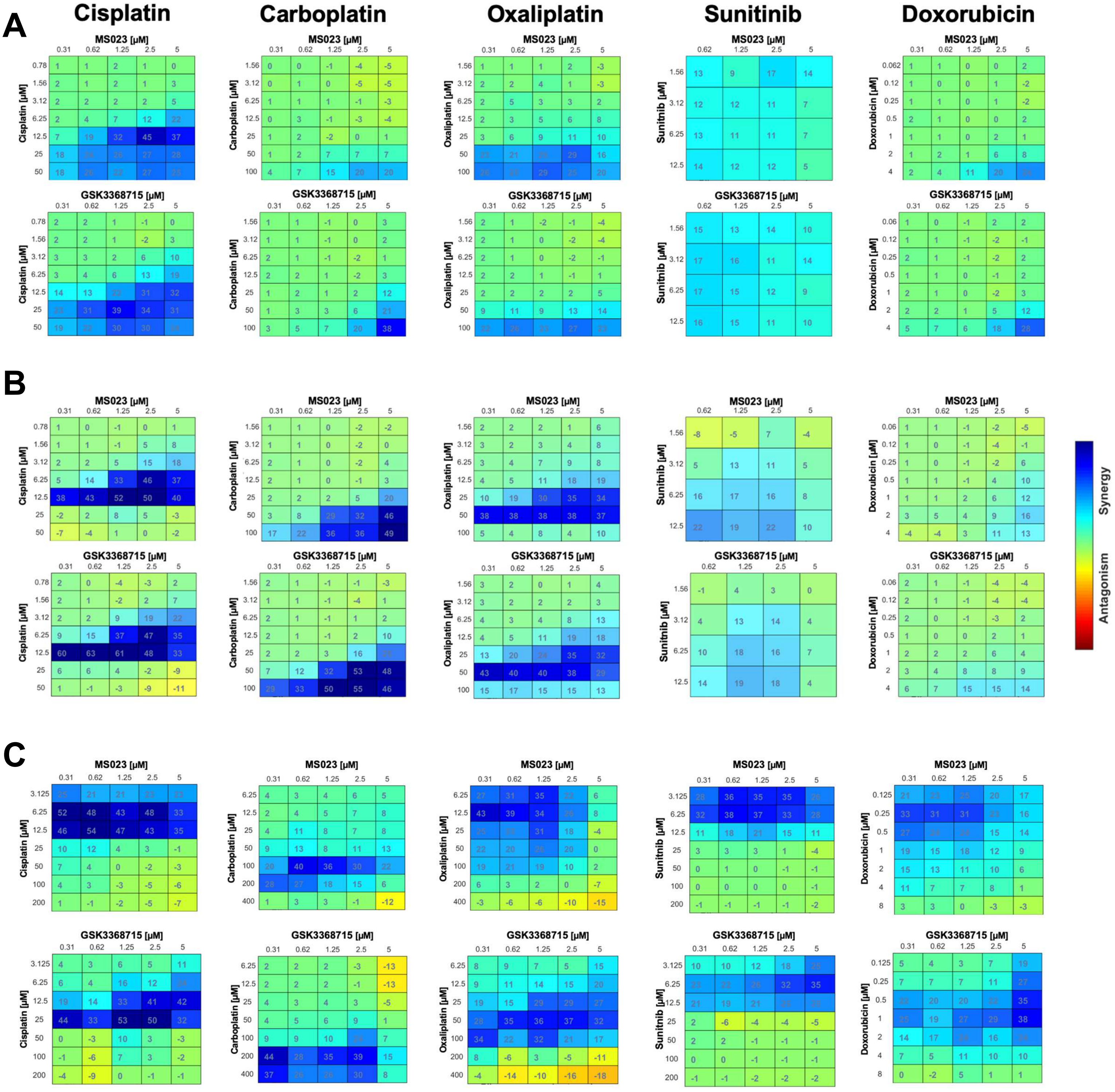

Supplementary Figure 6. Synergy scores of combinatorial treatments involving Type I PRMT inhibitors and chemotherapeutic agents indicate synergy on cancer cells. Cell viability assay results from combination treatments of the Type I PRMT inhibitors MS023 and GSK3368715 with chemotherapeutic agents in (**A**) Du145, (**B**) Panc1, and (**C**) H1299 cells were analyzed using the Combenefit software employing the BLISS model. Synergy status of each drug combinations is indicated in the synergy score matrix.

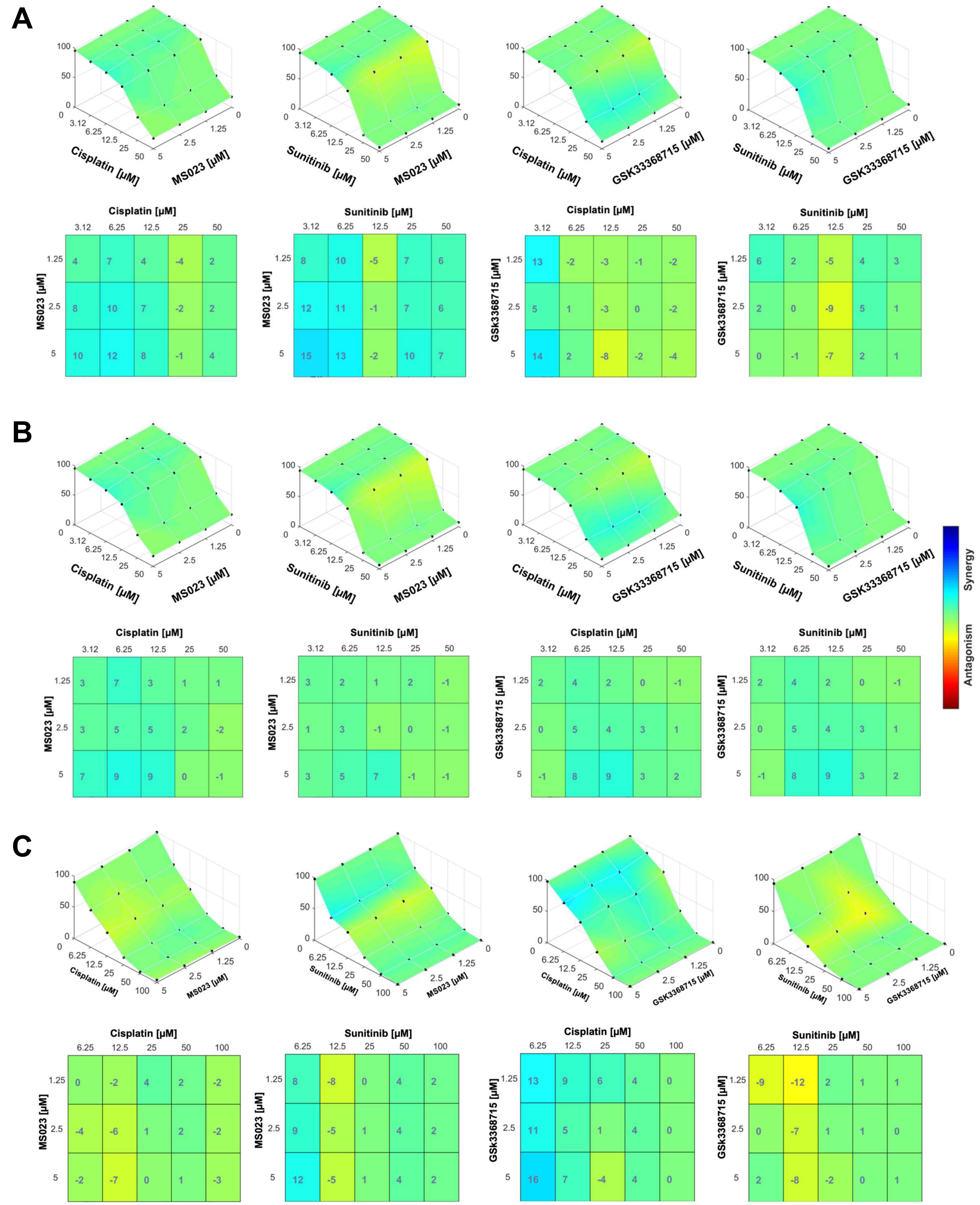

Supplementary Figure 7. Type I PRMT inhibitors MS023 and GSK3368715 do not synergize with chemotherapeutic agents without priming. Epidrugs were only co-treated with cisplatin or sunitinib for 72 h without priming on (**A**) Du145, (**B**) Panc1, and (**C**) H1299 cells. Epidrug and cisplatin/sunitinib combination doses were shown at the horizontal axis, whereas % cell viability values were plotted at the vertical axis. Calculated synergy scores are indicated below as a matrix.

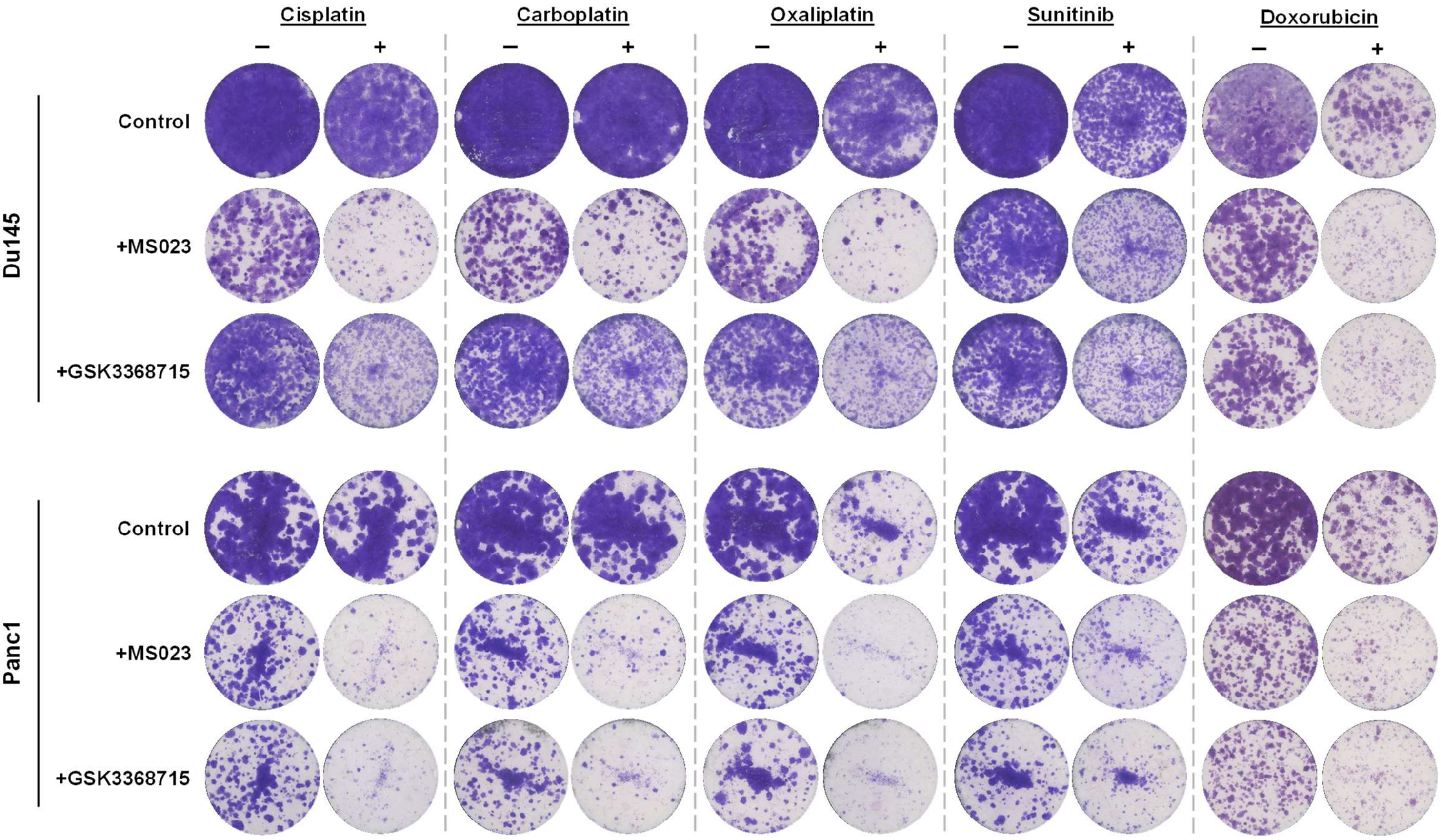

Supplementary Figure 8. Effect of synergy between Type I PRMT inhibitors and chemotherapeutic agents on colony formation capacity of cells. Colony formation assay performed on Du145 and Panc1 cells confirmed the synergistic effect through inhibition of colony forming capacity of cells after the combination of each epidrug with chemotherapeutic agents. Du145 cells and Panc1 cells were primed with epidrugs MS023 and GSK3368715 for 72 h, then combination treatments were carried out with epidrugs and indicated chemotherapeutic agents for 72 h.

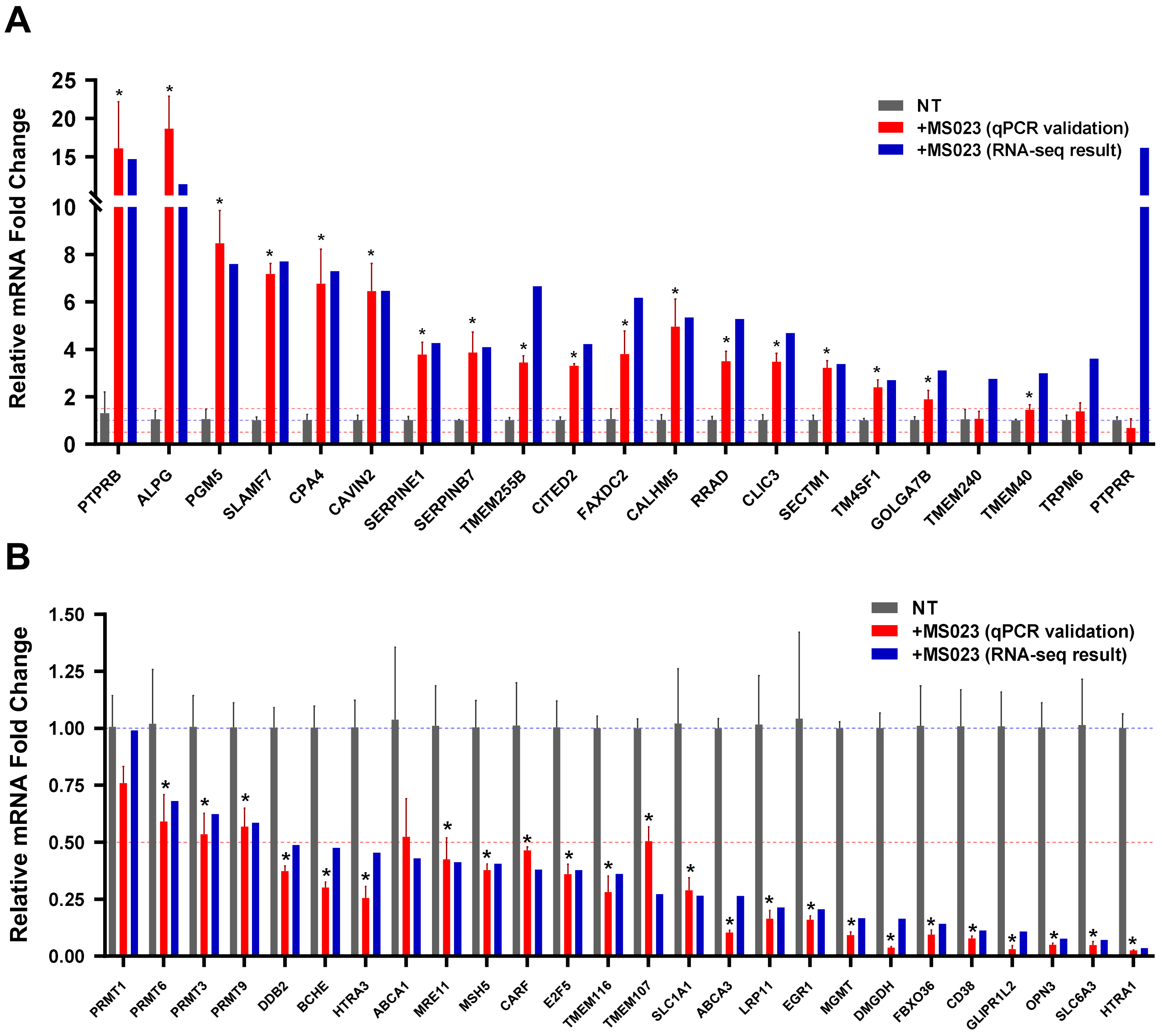

Supplementary Figure 9. Validation of mRNA expression change of top selected up- and down-regulated genes. Top (**A**) up- and (**B**) down-regulated genes were picked, and expression changes were validated via RT-qPCR. Error bars represent the standard deviation of three biological replicates (Student's t-test; *p<0.05).

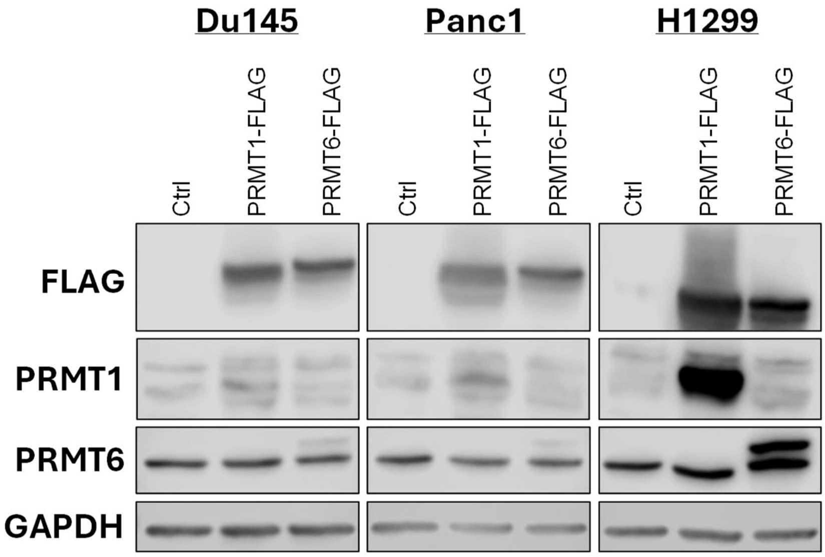

Supplementary Figure 10. Western blot experiments confirm the stable PRMT1/6-FLAG overexpression on Du145, Panc1, and H1299 cells. PRMT1 and PRMT6 constructs were cloned into pLJC2-3xFLAG lentiviral overexpression backbone and stable overexpression was validated via western blotting experiments using anti-FLAG, anti-PRMT1, and anti-PRMT6 antibodies. GAPDH was used as a loading control.

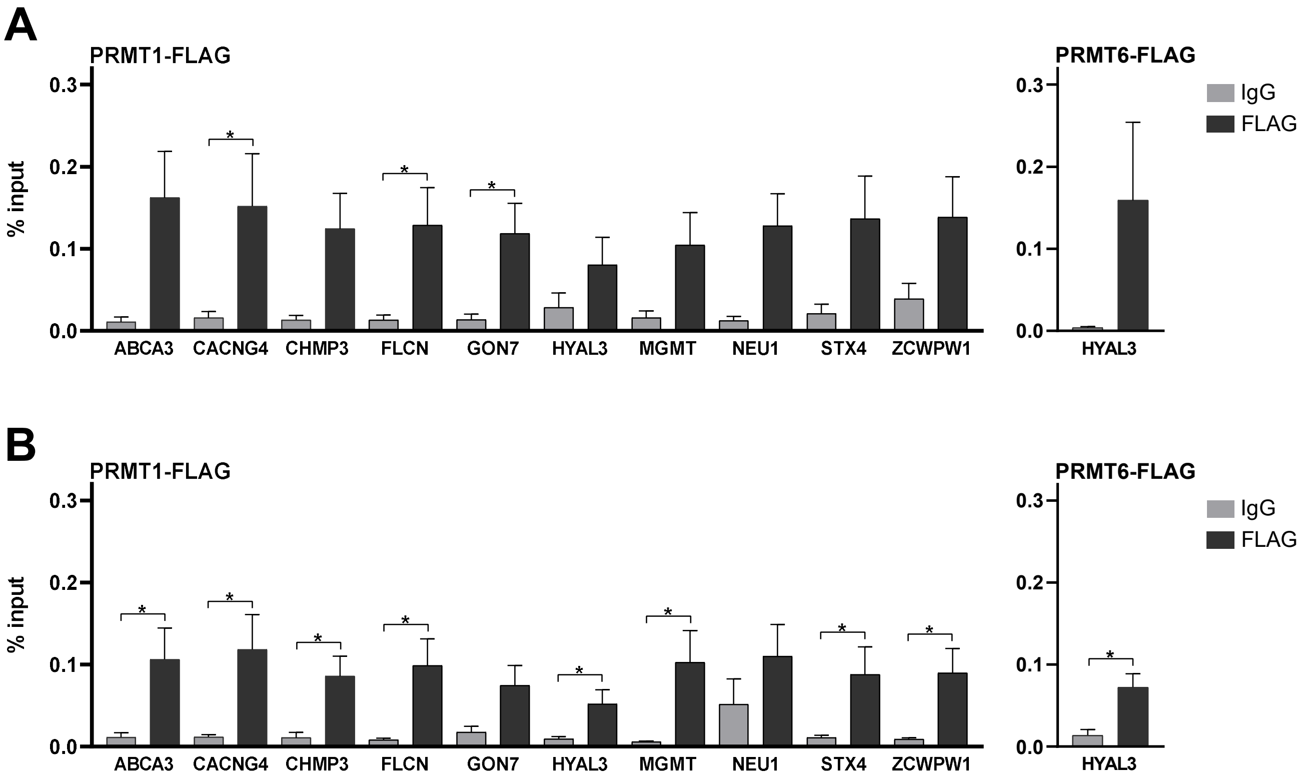

Supplementary Figure 11. ChIP-qPCR analysis confirm that promoter regions of selected genes found in combined gene sets are occupied by PRMT1 and PRMT6. PRMT1 and PRMT6 ChIP-qPCR experiments performed on PRMT1/6-FLAG overexpressing (**A**) Panc1 and (**B**) H1299 cells confirm that promoter regions of selected target genes are occupied by PRMT1 and PRMT6. Error bars indicate standard error of the mean (SEM) from three independent biological replicates done in duplicate (*$p<0.05$, Mann–Whitney U test).

Supplementary Table 1. Sequence information of primers used in the RT-qPCR experiments.

	Fwd Primer (5'-3')	Rev Primer (5'-3')
**ABCA1**	ACAAGATGCTGAGGGCTGATG	CCAGTTTCTCCCTTGGTAGGC
**ABCA2**	ACACCTCTGGTTCTACTCACGG	CCGACAATGTCTGCACCAGTGA
**ABCA3**	CTTGACAGTCGCAGAGCACCTT	CTCCGTGAGTTCCACTTGTCCT
**ALPG (ALPPL2)**	TACTCCATACCTGGGATTTCCG	TTCTCCTCCTCAACTGGGAT
**ATOX1**	TCTCTCGGGTCCTCAATAAGC	AAGCAGAGTGTCCATGCTGTG
**ATP6V0D1**	ACAAGACGCTGGAGGACCGATT	ACGATGTTGCGACACTCCTGCT
**ATP6V1A**	GAAACTTCTGGTGTGTCTGT	CCATAATGCCAGGACCAAG
**ATP7A**	TTTGGAACTTGTTGTGAGGGG	AGGGCCACGGAGCAGTATAG
**ATP7B**	GTGGGCAATGACACCACTTT	TGGGTGCCTTTGACATCTGA
**BCHE**	TCCATAGTGAAACGGTGGGC	AGGCCAGCTTGTGCTATTGT
**CALHM5**	GCAAGGGTAAGCCCAAAGAGTG	CGCTGAACAAATCAGGCACCATC
**CARF**	GAGATTATTCCAGCAACGCTTCA	ATTTTGGGCTGAAATGAGGAGAGTG
**CAVIN2**	GCGGTCAAAGAGCGCATGGATA	AAACACGCTGGCAGGGATCTCA
**CD38**	CAGCACTTTTGGGAGTGTGG	GATCCTGGCATAAGTCTCTGG
**CITED2**	TGCCGCCCAATGTCATAGACAC	CAGCTCCTTGATGCGGTCCAAA
**CLIC3 (CLI3)**	CATCCTGCTCTATGACAGCGAC	GGTGTTGGACTCCCTGTAACGA
**CPA4**	GGGGACATGAGGTGGATACTG	CCTCAAAACTTGGTCCCCAAAAA
**CTR1**	TGCGTAAGTCACAAGTCAGCA	AGGTGAGGAAAGCTCAGCATC
**CTSB**	GCTTCGATGCACGGGAACAATG	CATTGGTGTGGATGCAGATCCG
**CTSD**	GCAAACTGCTGGACATCGCTTG	GCCATAGTGGATGTCAAACGAGG
**DDB2**	TTGTGGGCCGATACCCAGAT	AAGCGAACTGATGCCAGAAGA
**DMGDH**	GGCTGAAACGAAGACTGGTCTG	GCTTCCAGATGTCGTGTTGCCA
**E2F5**	CCATTCAGGCACCTTCTGGTAC	AGCAGCACATGGATAGGTCCTG
**EGR1**	AGCAGCACCTTCAACCCTCAGG	GAGTGGTTTGGCTGGGGTAACT
**FAXDC2**	ACTGTGGCTACCACCTTCCCTT	GGTCTGCTTGAACATGGTGTCAG
**FBXO36**	TGGTCACCCGGTCTCAGGTAAT	GGGTTTCTTTTGCTTCTCCAGGT
**GAPDH**	CTGACTTCAACAGCGACACC	GTTGTCATACCAGGAAATGAGC
**GLIPR1L2**	TTTGCCAGACGAGGAGGACGTA	CCATGCTCTAGCAGTCCGTGAT
**GOLGA7B**	CGGATTTTACGCAGAGGCTGAG	CATTCTGCTCCTGGATGTAGCG
**HTRA1**	CAGACGTGATCTCAGGAGCGTA	TCGCTGACATCATTGGCGGAGA
**HTRA3**	CAAGAAGTCGGACATTGCCACC	CGTTGTCACTGTGTTCTGTAGGG
**LAMP1**	CGTGTCACGAAGGCGTTTTCAG	CTGTTCTCGTCCAGCAGACACT
**LAMP2**	GGCAATGATACTTGTCTGCTGGC	GTAGAGCAGTGTGAGAACGGCA
**LRP11**	GTCAGTGGACATGAAGGTGCC	GGAAGGTGTAGGTTCCCTCCT

	Fwd Primer (5'-3')	Rev Primer (5'-3')
**MCOLN1**	CGGACTGCTATACCTTCAGCGT	GGTGCTTACACTCCTGGATGTG
**MGMT**	CCTGGCTGAATGCCTATTTCCAC	GCAGCTTCCATAACACCTGTCTG
**MITF**	GGCTTGATGGATCCTGCTTTGC	GAAGGTTGGCTGGACAGGAGTT
**MRE11**	TGCCCAGGAAAATGAAGTGGA	CAGGCCGATCACCCATACAA
**MSH5**	CGAACCAAGGAGCTGGATGCAT	GGAGGCAAGGTCCAATACTCGG
**OPN3**	ATGGTCACCTGGTCACTCCAAC	GAGGCACAGAAGCTGCAAAAGG
**PGM5**	CTGATGGACTCAGGACGTTGCA	ATGGAGAGCCAGACCAAGACAG
**PPT1**	GGCGTACTCCAAAGTTGTTCAGG	CTGCCAAGAAGATGCTGTGGTTG
**PRMT1**	CCTCACTTACCGCAACTCCA	CTCGATCCCGATGACCTTGC
**PRMT3**	TGTCAGAACCTGCTCGTCAT	TCTCGGTAGCTTTCTGTTCGT
**PRMT6**	AAGATGTCGCAGCCCAAGAA	GGACCGAAACGTCCGAGTAG
**PRMT8**	AGCGAGTGGATGGGCTACT	CTGCCCGGTCTGGAAACATAA
**PRMT9**	CAGGACCTTGCAGACTACTGG	AGGTTAGCGAGGGCACTACA
**PTPRB**	TCTTCCCGACAAGTGGTTGTGG	AGCCAGGAAACGCTGAGGTAGT
**PTPRR**	GTAACCGATTCATTGAGCACCTAC	GCTGTCTTCCTGCCAAACCATC
**RRAD**	CGAGAGCGTTTACAAGGTGCTG	ATGGAGCGATCATAGGTGTGCC
**SECTM1**	GACAAGTCACGCTGGAGGTTTC	CACCTGTACCAGGCGAACATGA
**SERPINB7**	CGATGCCAAAGTGGAGCGAGTT	CAGCAGATGAGCTTATGCCACC
**SERPINE1**	GCCGCCTCTTCCACAAATCA	GGCAGTTCCAGGATGTCGTA
**SLAMF7**	ACTGTGGAAATACCGAAAAAGATGG	ATAGCCTTGGTGTGTCTGGC
**SLC1A1**	CGAAAGAACCCTTTCCGATTTGC	GAAGGTGACAGGCAGTGTTGCT
**SLC6A3**	CCTCAACGACACTTTTGGGACC	AGTAGAGCAGCACGATGACCAG
**TFEB**	CCTGGAGATGACCAACAAGCAG	TAGGCAGCTCCTGCTTCACCAC
**TM4SF1**	ATGCCTCCGAAAACCACCTC	TGGCAGGAGCATCAGCA
**TMEM107**	CGGAGCATTCTGGGAAGAAGT	TAACGCGCTGGTATGAGCAA
**TMEM116**	TCGTTTGCTGTCCTTCCTCTC	TGTATTGCAGGAAGAACCGAGG
**TMEM205**	GCAAATGTGGGTGACCTTCGTC	GCAGAGGTTGATGAAGGCACAG
**TMEM240**	CCGATTCCACAACTACATCCTCC	CGGGATCACGTAGTGGATATGG
**TMEM255B**	CATCGTGGACGGCGTATTTGCA	ACAGGTGACCTCTGTCTGGTAG
**TMEM40**	TTTCGTCCTCCTGTGCTTTGCC	GTCCGAAGTAGATGCCAACGGT
**TPP1**	GGTGGCTTCAGCAATGTGTTCC	GAAGTAACTGGATGGTGGCAGG
**TRPM6**	ACCCTTCTTTCAGGCTACCG	GGAGTGTCTCTGGTGCTTGT
**VAMP7**	CGGTTCAAGAGCACAGACAGCA	ATCCACTTGGGCTTGAGTCTCC

Supplementary Table 2. Primers used in the ChIP-qPCR experiments and PRMT1/6 cloning into pLJC2 lentiviral overexpression vector.

**ChIP_STX4_Fwd**	TGAGAACGGCGTCCCAGAGA
**ChIP_STX4_Rev**	CCAAACTCGTGGACCGCAGA
**ChIP_CACNG4_Fwd**	CCGGCGATTTCTGGTTGATTGG
**ChIP_CACNG4_Rev**	GGCTGGTTGAAAAGCCAGGG
**ChIP_CHMP3_Fwd**	GCGCAGAATCGAGTGAGTGG
**ChIP_CHMP3_Rev**	AGCACCGAATCCGTGGTCTC
**ChIP_HYAL3_Fwd**	ACAGCTTGGAAACGGTCCGA
**ChIP_HYAL3_Rev**	AGTCTAAGGGCGGCTGCTTC
**ChIP_MGMT_Fwd**	GGGGTAGTTTGATCCCTTCGGT
**ChIP_MGMT_Rev**	GAGAGACCACTCTGCAGGTCT
**ChIP_ZCWPW1_Fwd**	GCGCCTAGCACCTTCACCTA
**ChIP_ZCWPW1_Rev**	GTTCCGCCCATCGCCTTTAG
**ChIP_GON7_Fwd**	CATCCGTTTGTCGGAAGTCGC
**ChIP_GON7_Rev**	CGAGTGCCAGGAACCAATGAC
**ChIP_FLCN_Fwd**	CGCTGCCCGACAATTACCCT
**ChIP_FLCN_Rev**	GTGCGGTCTGAACCATTCGG
**ChIP_NEU1_Fwd**	CGCGCTTCCCGGACTCTAAT
**ChIP_NEU1_Rev**	AAGAGGGCCAATCGGAAGGG
**ChIP_ABCA3_Fwd**	GGTGGCCTCTGACAGGAATGAC
**ChIP_ABCA3_Rev**	ATGGAGAAACGGCCAAAGTCG
**PRMT1-PacI_Fwd**	atatatTTAATTAAATGGCGGCAGCCGAGGCCGC
**PRMT1-NotI_Rev**	atatatatGCGGCCGCGCGCATCCGGTAGTCGG
**PRMT6-PacI_Fwd**	gcgcgcgcTTAATTAAATGTCTCAGCCTAAGA
**PRMT6-NotI_Rev**	atatatGCGGCCGCATCCTCCATGGCGAAGTCTTT

Supplementary Table 3. Information about type I PRMT inhibitors and their inhibition IC50 values on target PRMTs

**MS023** (CAS; 1831110-54-3) 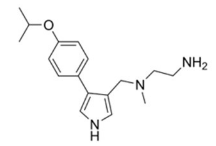	**Inhibition IC_{50}**				
	PRMT1	PRMT3	PRMT4/CARM1	PRMT6	PRMT8
	30 nM	119 nM	83 nM	4 nM	5 nM
**GSK3368715** (CAS; 1629013-22-4) 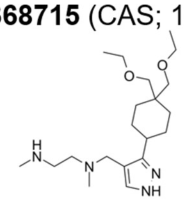	**Inhibition IC_{50}**				
	PRMT1	PRMT3	PRMT4/CARM1	PRMT6	PRMT8
	3.1 nM	48 nM	1148 nM	5.7 nM	1.7 nM

Supplementary Table 4. The list of gene sets being used to curate combined gene sets for RNA-seq analysis

**Exocytosis-related Gene Sets**
GO:0006887, GO:0045955, GO:2000301, GO:0045956, GO:0045055, GO:0017158, GO:0017157, GO:2000300, GO:0016079
**Lysosome-related Gene Sets**
GO:0008333, GO:0032510, GO:0090160, GO:0006622, GO:1905671, GO:0062196, GO:0150031, GO:0004563, GO:0015929, GO:0007042, GO:0097212, GO:1905146, GO:0007041, GO:0035751, GO:1905165, GO:0033299, GO:0098574, GO:0043202, GO:0097401, GO:0007035, HP:0004356
**Sequestering-related Gene Sets**
GO:0051238, GO:0140487, GO:0140313, GO:0140311
**Methyltranferase-related Gene Sets**
GO:0035242, GO:0008276, GO:0008169, GO:0034708, GO:0035097, GO:0140939
**DNA Repair-related Gene Sets**
GO:0006281, GO:0006302, GO:0036297, GO:0006289, GO:1990391, WP4946, WP4753, R-HSA-5693532, R-HSA-73894, R-HSA-5696398, hsa03420, Pubmed 26771021, Pubmed 17891185
**RNA Processing-related Gene Sets**
GO:0008380, GO:0001510, GO:0009451, GO:0003723

Supplemental Material: Epidrug Library Content

The following supplementary material section includes the content of the epidrug library used in the study

Location on library plates	Cayman Item No. (Cat No.)	Epidrug Name
1-A2	10496	Sulforaphane
1-A3	10875	UNC669
1-A4	10443	SB939
1-A5	10444	PCI 34051
1-A6	11876	L-α-Hydroxyglutaric Acid (sodium salt)
1-A7	10523	Sirtinol
1-A8	13856	5-Nitroso-8-quinolinol
1-A9	10549	C646
1-A10	14088	JNJ-26481585 (hydrochloride)
1-A11	10566	Garcinol
1-B2	14314	Plumbagin
1-B3	10572	Scriptaid
1-B4	10574	Suberohydroxamic Acid
1-B5	10575	Apicidin
1-B6	14969	LMK 235
1-B7	10582	UNC0321 (trifluoroacetate salt)
1-B8	15200	HDAC6 Inhibitor
1-B9	10599	Cl-Amidine (hydrochloride)
1-B10	15487	SP-2509
1-B11	10641	JGB1741
1-C2	16081	A-366
1-C3	10676	I-BET762
1-C4	10734	UNC0638
1-C5	16095	OICR-9429
1-C6	16173	EPZ004777 (formate)
1-C7	16174	EPZ6438
1-C8	16272	PBIT
1-C9	10975	Zebularine
1-C10	11012	Delphinidin (chloride)
1-C11	11045	ITF 2357
1-D2	16365	Octyl-α-hydroxyglutarate
1-D3	16400	UNC0379
1-D4	11091	Methylstat (hydrate)
1-D5	16615	BVT 948
1-D6	16918	BG45
1-D7	17123	UMB-32
1-D8	11138	2,4-Pyridinedicarboxylic Acid (hydrate)
1-D9	11155	PFI-1
1-D10	11164	5-Azacytidine
1-D11	11165	SGI-1027
1-E2	11166	Decitabine
1-E3	11181	I-BET151
1-E4	11187	(+)-JQ1
1-E5	17285	EPZ015666
1-E6	17385	BI-2536
1-E7	17448	BAZ2-ICR
1-E8	11323	Sodium 4-Phenylbutyrate
1-E9	17471	OG-L002
1-E10	11572	IOX1
1-E11	11620	MI-2 (hydrochloride)
1-F2	17472	ML-324
1-F3	11690	Gemcitabine

1-F4	17488	GSK484 (hydrochloride)
1-F5	17553	Resminostat (hydrochloride)
1-F6	17662	NI-57
1-F7	12033	Daminozide
1-F8	12054	GSK-J1 (sodium salt)
1-F9	17663	PFI-4
1-F10	12073	GSK-J4 (hydrochloride)
1-F11	17692	Tasquinimod
1-G2	12084	CI-994
1-G3	12086	CPTH2 (hydrochloride)
1-G4	17699	MM-102
1-G5	17718	TC-E 5003
1-G6	12095	Butyrolactone 3
1-G7	17778	L002
1-G8	13085	Tenovin-1
1-G9	13086	Tenovin-6 (hydrochloride)
1-G10	17897	BI-9564
1-G11	18124	RN-1 (hydrochloride)
1-H2	13144	Anacardic Acid
1-H3	13145	AGK2
1-H4	13146	CAY10603
1-H5	13168	Splitomicin
1-H6	13172	CBHA
1-H7	18287	Mocetinostat
1-H8	13176	Oxamflatin
1-H9	13178	Salermide
1-H10	18317	A-196
1-H11	18348	MS049 (hydrochloride)
2-A2	18354	GSK591
2-A3	13280	Panobinostat
2-A4	13284	MS-275
2-A5	18361	MS023 (hydrochloride)
2-A6	13302	RG108
2-A7	13373	2',3',5'-triacetyl-5-Azacytidine
2-A8	19093	PRT4165
2-A9	13631	UNC0224
2-A10	19136	ORY-1001
2-A11	19160	EPZ020411
2-B2	13828	3-Deazaneplanocin A
2-B3	13829	Sinefungin
2-B4	13870	Pyroxamide
2-B5	13944	N-Oxalylglycine
2-B6	19403	GSK2879552
2-B7	13965	AMI-1 (sodium salt)
2-B8	13966	EPZ005687
2-B9	13967	SGC0946
2-B10	13968	UNC1215
2-B11	19551	Cyproheptadine (hydrochloride hydrate)
2-C2	14094	GSK343
2-C3	14119	Bromosporine
2-C4	14120	GSK2801
2-C5	14407	SIRT1/2 Inhibitor IV
2-C6	14468	I-CBP112 (hydrochloride)
2-C7	14469	SGC-CBP30
2-C8	14604	UNC0642
2-C9	14621	UNC1999
2-C10	14678	(R)-PFI-2 (hydrochloride)

2-C11	15066	HPOB
2-D2	19828	CPTH6 (hydrobromide)
2-D3	15267	PFI-3
2-D4	15338	JIB-04
2-D5	15403	CAY10683
2-D6	15415	GSK126
2-D7	15479	CPI-203
2-D8	15774	6-Thioguanine
2-D9	19835	HAT Inhibitor II
2-D10	19956	CeMMEC1
2-D11	15947	OTX015
2-E2	20059	PCI 24781
2-E3	20209	BML-278
2-E4	20224	CeMMEC13
2-E5	16175	EPZ5676
2-E6	16265	MC 1568
2-E7	20541	4'-bromo-Resveratrol
2-E8	16374	α-Hydroxyglutaric Acid (sodium salt)
2-E9	20864	AZD 5153
2-E10	16424	RVX-208
2-E11	20893	Todralazine (hydrochloride)
2-F2	16427	LAQ824
2-F3	21057	HDAC3 Inhibitor
2-F4	16439	GSK-LSD1 (hydrochloride)
2-F5	21159	CXD101
2-F6	16917	RGFP966
2-F7	16919	BRD73954
2-F8	21234	NCH-51
2-F9	21273	UF010
2-F10	21568	RGFP109
2-F11	89730	Trichostatin A
2-G2	89740	CAY10398
2-G3	9001839	RSC-133
2-G4	10005019	BML-210
2-G5	21601	C-7280948
2-G6	10009797	CAY10591
2-G7	22031	EED226
2-G8	10009929	SAHA
2-G9	10010494	Tranylcypromine (hydrochloride)
2-G10	22404	CAY10722
2-G11	22942	PAOA
2-H2	23242	TMP-195
2-H3	26068	Bufexamac
2-H4	11706	BAPTA
2-H5	21601	C-7280948
2-H6	34886	GSK3368715
2-H7	21879	SGC2085
2-H8	17718	TC-E 5003
2-H9	19160	EPZ020411
2-H10	Selleckchem #S6414	Apilimod
2-H11	Unused	Unused (DMSO)
3-A2	11085	UNC0646
3-B2	13124	BIX01294 (hydrochloride hydrate)
3-C2	16614	Ryuvidine
3-D2	17017	SGC707
3-E2	20425	Vacuolin-1
3-F2	11796	Wedelolactone
3-G2	13148	Lysine-specific Demethylase Inhibitor (1C) (hydrochloride)
3-H2	13212	Pimelic Diphenylamide 106

3-A3	13686	Chidamide
3-B3	16874	Nexturastat A
3-C3	20985	GSK6853
3-D3	9002910	(+)-JQ1 (free acid)
3-E3	21273	UF010
3-F3	10004974	Ionomycin
3-G3	10007965	FK-506

Supplemental Material: Online ChIP-seq Data Interpretation

The following supplementary material section includes interpretation of online available ChIP-seq Data retrieved for this study

Target Gene Name	Combined Gene Set Number<br>**1.**Exocytosis<br>**2.** Lysosome<br>**3.** Sequestering<br>**4.** Methylation<br>**5.** DNA Repair<br>**6.** RNA Processing	RNA-seq Fold Change	PRMT1 ChIP-seq Data (Cell Type/Cell Line, Enrichment Peak Score, ChIP-seq Data ID)					PRMT4/CARM1 ChIP-seq Data (Cell Type/Cell Line, Enrichment Peak Score, ChIP-seq Data ID)		PRMT6 ChIP-seq Data (Cell Type/Cell Line, Enrichment Peak Score, ChIP-seq Data ID)
AGO2	6	2.15	Keratinocytes, 227.0, SRX3642946	Keratinocytes, 308.0, SRX3642947	-	-	-	Kasumi-1, 249.0, SRX15946676	Kasumi-1, 296.0, SRX15946675	-
APLF	5	0.48	Keratinocytes, 146.0, SRX3642947	Keratinocytes, 93.0, SRX3642946	-	-	-	Kasumi-1, 136.0, SRX15946675	-	-
ARC	6	2.08	-	-	-	-	-	-	-	-
ASCC1	5, 6	0.48	Keratinocytes, 90.0, SRX3642947	-	-	-	-	-	-	-
BCLAF1	6	0.47	Keratinocytes, 112.0, SRX3642946	Keratinocytes, 104.0, SRX3642947	-	-	-	-	-	-
BRAF	1	2.56	-	-	-	-	-	-	-	-
C7orf50	6	0.43	Keratinocytes, 101.0, SRX3642947	-	-	-	-	-	-	-
CACNG4	2	0.33	Keratinocytes, 476.0, SRX3642946	Keratinocytes, 424.0, SRX3642947	Keratinocytes, 100.0, SRX3642948	-	-	MCF-7, 265.0, SRX5185601	-	-
CCNA1	5	2.08	Keratinocytes, 114.0, SRX3642946	-	-	-	-	-	-	-
CELF5	6	2.14	-	-	-	-	-	-	-	-
CHMP3	1, 2	0.25	Keratinocytes, 149.0, SRX3642946	Keratinocytes, 168.0, SRX3642947	Keratinocytes, 253.0, SRX3642946	Keratinocytes, 302.0, SRX3642947	-	Kasumi-1, 228.0, SRX15946675	-	-
CLTRN	1	0.31	-	-	-	-	-	-	-	-
CUBN	2	0.23	Keratinocytes, 160.0, SRX3642946	-	-	-	-	-	-	-
DDB2	5	0.49	Keratinocytes, 133.0, SRX3642947	Keratinocytes, 102.0, SRX3642947	-	-	-	-	-	-
DHFR2	6	0.5	Keratinocytes, 72.0, SRX3642947	-	-	-	-	-	-	-
DMGDH	6	0.16	-	-	-	-	-	-	-	-
DUS4L	6	0.42	Keratinocytes, 109.0, SRX3642946	Keratinocytes, 81.0, SRX3642946	Keratinocytes, 130.0, SRX3642947	-	-	Kasumi-1, 89.0, SRX15946675	-	-
DYRK1B	5	2.13	Keratinocytes, 83.0, SRX3642947	-	-	-	-	MCF-7, 151.0, SRX5185600	MCF-7, 244.0, SRX5185601	-
EGFR	5	0.38	Keratinocytes, 158.0, SRX3642946	Keratinocytes, 166.0, SRX3642947	-	-	-	-	-	-
ERCC6	5	2	Keratinocytes, 351.0, SRX3642947	Keratinocytes, 408.0, SRX3642946	-	-	-	-	-	-
ERCC8	5	0.46	-	-	-	-	-	Kasumi-1, 71.0, SRX15946675	-	-
F2RL1	1	2.06	Keratinocytes, 151.0, SRX3642947	Keratinocytes, 119.0, SRX3642946	-	-	-	-	-	-
FAM120C	6	0.3	-	-	-	-	-	-	-	-
FASN	6	2.25	Keratinocytes, 139.0, SRX3642947	-	-	-	-	-	-	-
FLCN	2	2.07	Keratinocytes, 179.0, SRX3642947	Keratinocytes, 125.0, SRX3642946	-	-	-	-	-	-
GALNS	2	0.35	-	-	-	-	-	-	-	-
GEN1	5	0.47	Keratinocytes, 64.0, SRX3642946	-	-	-	-	Kasumi-1, 138.0, SRX15946675	-	-
GON7	6	0.33	Keratinocytes, 145.0, SRX3642946	Keratinocytes, 137.0, SRX3642947	-	-	-	Kasumi-1, 249.0, SRX15946676	Kasumi-1, 255.0, SRX15946675	-
GRB7	6	2.1	Keratinocytes, 117.0, SRX3642946	Keratinocytes, 114.0, SRX3642947	Keratinocytes, 193.0, SRX3642947	Keratinocytes, 121.0, SRX3642946	-	-	-	-
GRIK5	1	0.25	-	-	-	-	-	-	-	-
H1-0	6	0.44	-	-	-	-	-	-	-	-
H2AJ	5	0.48	-	-	-	-	-	-	-	-
H2BC15	5	0.29	Keratinocytes, 99.0, SRX3642947	Keratinocytes, 135.0, SRX3642946	-	-	-	-	-	-
HLA-A	6	1.99	-	-	-	-	-	-	-	-
HSPB1	6	2.66	Keratinocytes, 186.0, SRX3642946	Keratinocytes, 235.0, SRX3642947	-	-	-	-	-	-
HYAL3	1, 2	0.43	Keratinocytes, 261.0, SRX3642947	Keratinocytes, 148.0, SRX3642946	-	-	-	-	-	MDA-MB-231, 98.0, SRX17024137
IFFO1	5	4.22	-	-	-	-	-	-	-	-
IFIT1	6	2.26	-	-	-	-	-	-	-	-
IFIT2	6	2.47	-	-	-	-	-	-	-	-
INSIG1	3	2.18	-	-	-	-	-	MCF-7, 102.0, SRX5185601	-	-
ISG15	5	2.28	Keratinocytes, 186.0, SRX3642947	Keratinocytes, 101.0, SRX3642946	-	-	-	-	-	-
ITGB2	1	2.48	Keratinocytes, 283.0, SRX3642947	Keratinocytes, 172.0, SRX3642946	-	-	-	-	-	MDA-MB-231, 132.0, SRX17024137
JAKMIP1	6	0.45	-	-	-	-	-	-	-	-
KCTD12	6	0.48	Keratinocytes, 120.0, SRX3642946	Keratinocytes, 158.0, SRX3642946	Keratinocytes, 105.0, SRX3642947	Keratinocytes, 133.0, SRX3642947	-	-	-	-
KLRC2	1	3.81	-	-	-	-	-	-	-	-
KRT18	6	2.07	Keratinocytes, 146.0, SRX3642946	-	-	-	-	-	-	-
MCMDC2	5	0.33	-	-	-	-	-	-	-	-
MGMT	5	0.17	Keratinocytes, 205.0, SRX3642947	-	-	-	-	-	-	-
MIF4GD	6	0.34	-	-	-	-	-	-	-	-
MRE11	5	0.41	-	-	-	-	-	-	-	-
MRM1	6	0.32	-	-	-	-	-	-	-	-
MSH5	5	0.41	-	-	-	-	-	-	-	-
NAGLU	2, 3	0.46	Keratinocytes, 164.0, SRX3642946	Keratinocytes, 183.0, SRX3642947	-	-	-	-	-	-
NEU1	2	2.1	Keratinocytes, 166.0, SRX3642947	Keratinocytes, 85.0, SRX3642947	-	-	-	-	-	-
NPR2	5	2.86	Keratinocytes, 105.0, SRX3642947	-	-	-	-	-	-	-
NR0B1	6	0.44	-	-	-	-	-	-	-	-
NR4A3	1	2.59	-	-	-	-	-	-	-	-
OASL	6	2.51	Keratinocytes, 110.0, SRX3642946	-	-	-	-	-	-	-
OOEP	5, 6	0.45	-	-	-	-	-	-	-	-
PCDHGA9	6	10.18	-	-	-	-	-	-	-	-
PCID2	6	0.42	Keratinocytes, 107.0, SRX3642947	Keratinocytes, 164.0, SRX3642946	-	-	-	-	-	-
PIWIL4	6	0.38	-	-	-	-	-	-	-	-
PLBD2	2	2.24	Keratinocytes, 105.0, SRX3642947	Keratinocytes, 79.0, SRX3642946	-	-	-	-	-	-
PRIMPOL	5	0.46	-	-	-	-	-	-	-	-
PRRT2	1	2.67	-	-	-	-	-	-	-	-
RAB15	1	2.42	-	-	-	-	-	-	-	-
RAB26	1	2.65	Keratinocytes, 125.0, SRX3642946	Keratinocytes, 167.0, SRX3642947	-	-	-	-	-	-

RAD54B	5	0.47	-	-	-	-	-	-	-	-
RBM44	6	0.42	Keratinocytes, 146.0, SRX3642946	-	-	-	-	-	-	-
REXO5	6	0.49	Keratinocytes, 143.0, SRX3642946	-	-	-	-	Kasumi-1, 175.0, SRX15946675	Kasumi-1, 120.0, SRX15946676	-
SDC2	2	2.31	Keratinocytes, 81.0, SRX3642947	-	-	-	-	-	-	-
SEPSECS	6	0.46	Keratinocytes, 469.0, SRX3642947	Keratinocytes, 437.0, SRX3642946	-	-	-	-	-	-
SLPI	6	2.45	-	-	-	-	-	-	-	-
SLX1B	5	0.33	Keratinocytes, 181.0, SRX3642946	Keratinocytes, 208.0, SRX3642947	-	-	-	Kasumi-1, 139.0, SRX15946676	Kasumi-1, 181.0, SRX15946675	-
SORL1	2	2.18	Keratinocytes, 200.0, SRX3642947	Keratinocytes, 166.0, SRX3642947	Keratinocytes, 190.0, SRX3642946	-	-	-	-	-
SRBD1	6	0.46	-	-	-	-	-	Kasumi-1, 190.0, SRX15946675	Kasumi-1, 210.0, SRX15946676	-
STX4	1	2.07	Keratinocytes, 120.0, SRX3642946	Keratinocytes, 189.0, SRX3642947	-	-	-	-	-	-
SYNJ2	6	2.53	Keratinocytes, 79.0, SRX3642946	-	-	-	-	-	-	-
TAF9	4, 5, 6	0.48	Keratinocytes, 207.0, SRX3642946	Keratinocytes, 171.0, SRX3642947	-	-	-	-	-	-
TP53	5, 6	0.39	Keratinocytes, 119.0, SRX3642947	Keratinocytes, 195.0, SRX3642946	Keratinocytes, 113.0, SRX3642947	Keratinocytes, 167.0, SRX3642946	Keratinocytes, 162.0, SRX3642947	-	-	-
TRAK1	2	2.23	-	-	-	-	-	-	-	-
TRMT10A	6	0.44	Keratinocytes, 109.0, SRX3642947	Keratinocytes, 112.0, SRX3642946	Keratinocytes, 174.0, SRX3642946	Keratinocytes, 76.0, SRX3642948	-	Kasumi-1, 178.0, SRX15946675	-	-
TST	6	0.39	-	-	-	-	-	-	-	-
UFL1	5	0.47	Keratinocytes, 193.0, SRX3642947	Keratinocytes, 180.0, SRX3642946	Keratinocytes, 97.0, SRX3642947	-	-	Kasumi-1, 106.0, SRX15946675	-	-
UNC13A	1	5.39	-	-	-	-	-	-	-	-
ZC3H6	6	0.37	Keratinocytes, 203.0, SRX3642947	Keratinocytes, 83.0, SRX3642946	-	-	-	Kasumi-1, 160.0, SRX15946675	-	-
ZCWPW1	5	0.23	Keratinocytes, 120.0, SRX3642947	Keratinocytes, 153.0, SRX3642946	Keratinocytes, 233.0, SRX3642947	Keratinocytes, 84.0, SRX3642946	-	Kasumi-1, 111.0, SRX15946675	-	-
ZRANB3	5	0.24	Keratinocytes, 104.0, SRX3642947	-	-	-	-	-	-	-

Supplemental Material: Western Blot Raw Data

The following supplementary material section includes uncropped raw data version of Western Blot images

Image ID: 0000528_01
Acquire Time: 18.Kas.2023 10:38:29

Acquisition Information

#	Image ID	Acquire Time	Channels	Integration Times	Analysis	Image Name	Comment	Image Modifications
1	0000528_01	18.Kas.2023 10:38:29	Chemi 700	05:30 00:30	Manual	0000528_01		

Image Display Values

Channel	Color	Minimum	Maximum	K
Chemi	Gray Scale (Black on White)	0,000328	0,00472	0
700	Gray Scale (Black on White)	0,0000454	0,0111	0

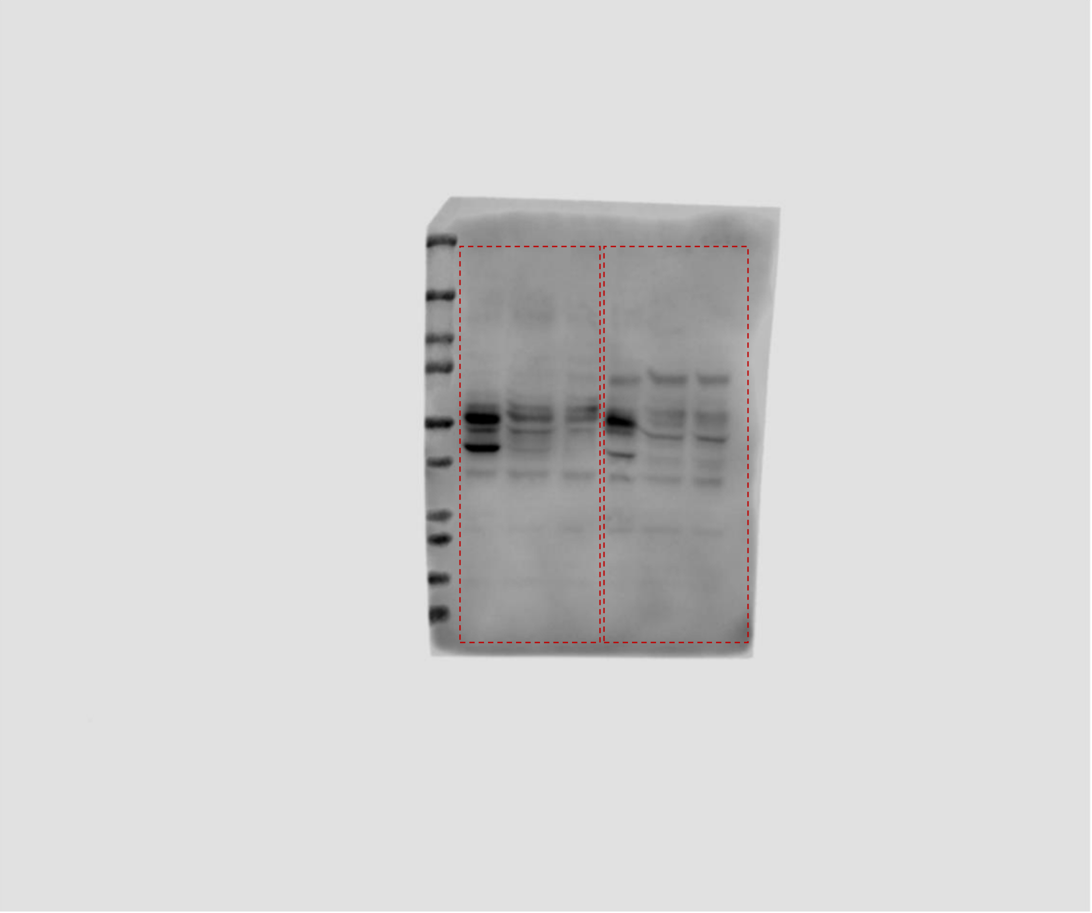

aDMA Western Blot
(Figure 4E)

Du145 and Panc1 samples were loaded sequentially

(Bands shown on the figure are framed in red on the raw data)

LI-COR®

Image ID: 0002868_02
Acquire Time: 24.ub.2025 08:11:55

Acquisition Information

#	Image ID	Acquire Time	Channels	Integration Times	Analysis	Image Name	Comment
1	0002868_02	24.ub.2025 08:11:55	Chemi 600	02:00 00:30	Manual	0002868_02	240225 h1299 adma 2min 2

Image Display Values

Channel	Color	Minimum	Maximum	K
Chemi	Gray Scale (Black on White)	0,0000218	0,0325	1
600	Gray Scale (Black on White)	0,0134	52,1	0

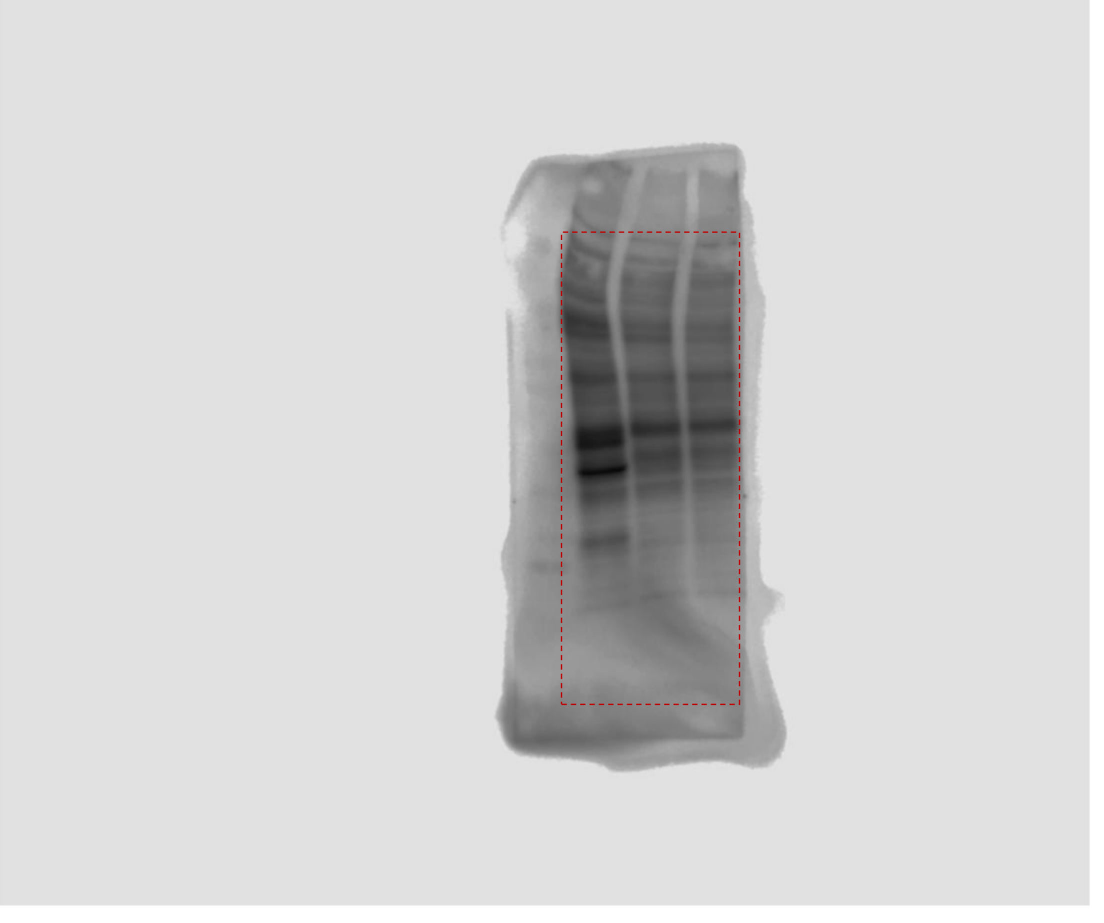

aDMA Western Blot
(Figure 4E)

H1299 samples were loaded

(Bands shown on the figure are framed in red on the raw data)

Image ID: 0000538_01
Acquire Time: 19.Kas.2023 09:45:49

Acquisition Information

#	Image ID	Acquire Time	Channels	Integration Times	Analysis	Image Name	Comment	Image Modifications
1	0000538_01	19.Kas.2023 09:45:49	Chemi 700	05:30 00:30	Manual	0000538_01		

Image Display Values

Channel	Color	Minimum	Maximum	K
Chemi	Gray Scale (Black on White)	0,0000824	0,00568	0
700	Gray Scale (Black on White)	0,000178	0,0966	0

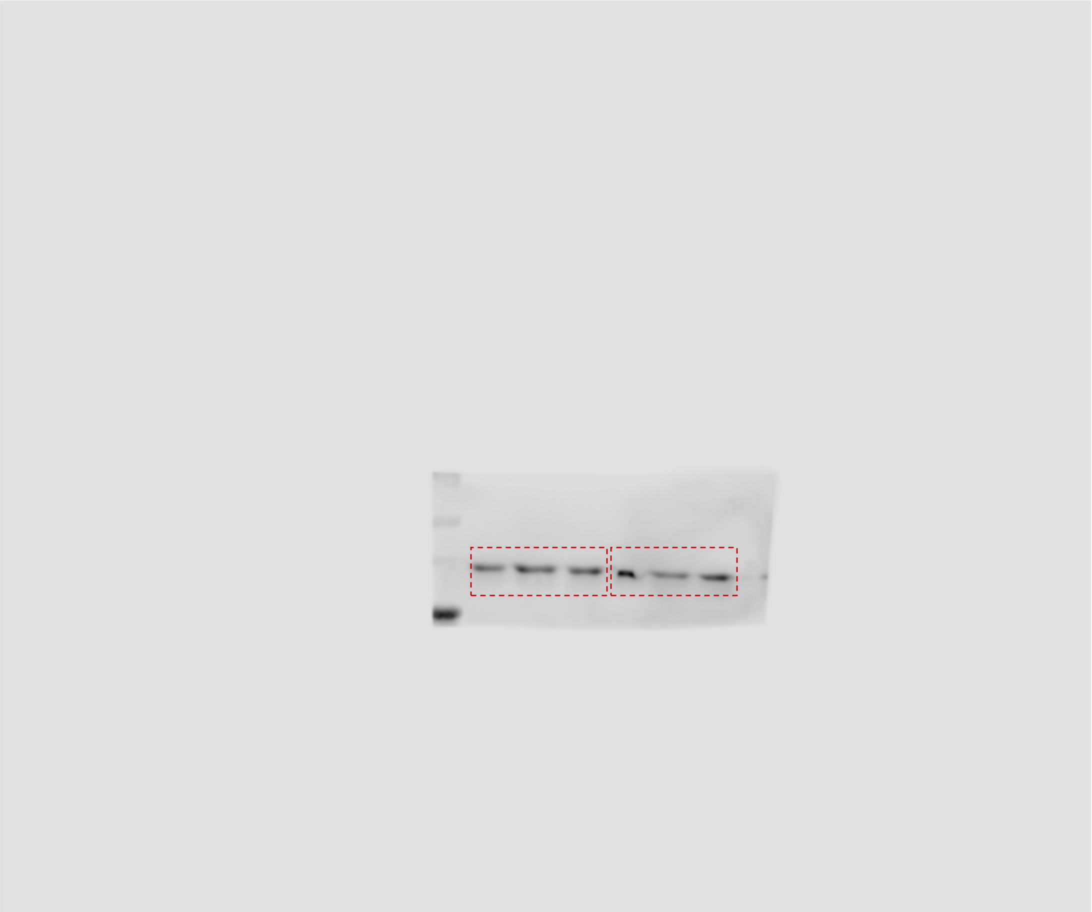

GAPDH Western Blot
(Figure 4E)

Du145 and Panc1 samples were loaded sequentially

(Bands shown on the figure are framed in red on the raw data)

Image ID: 0002869_01
Acquire Time: 24.ub.2025 08:16:40

Acquisition Information

#	Image ID	Acquire Time	Channels	Integration Times	Analysis	Image Name	Comment
1	0002869_01	24.ub.2025 08:16:40	Chemi 600	02:00 00:30	Manual	0002869_01	240225 gapdh bsa 2min

Image Display Values

Channel	Color	Minimum	Maximum	K
Chemi	Gray Scale (Black on White)	0,000272	0,0306	0
600	Gray Scale (Black on White)	0,00154	0,695	0

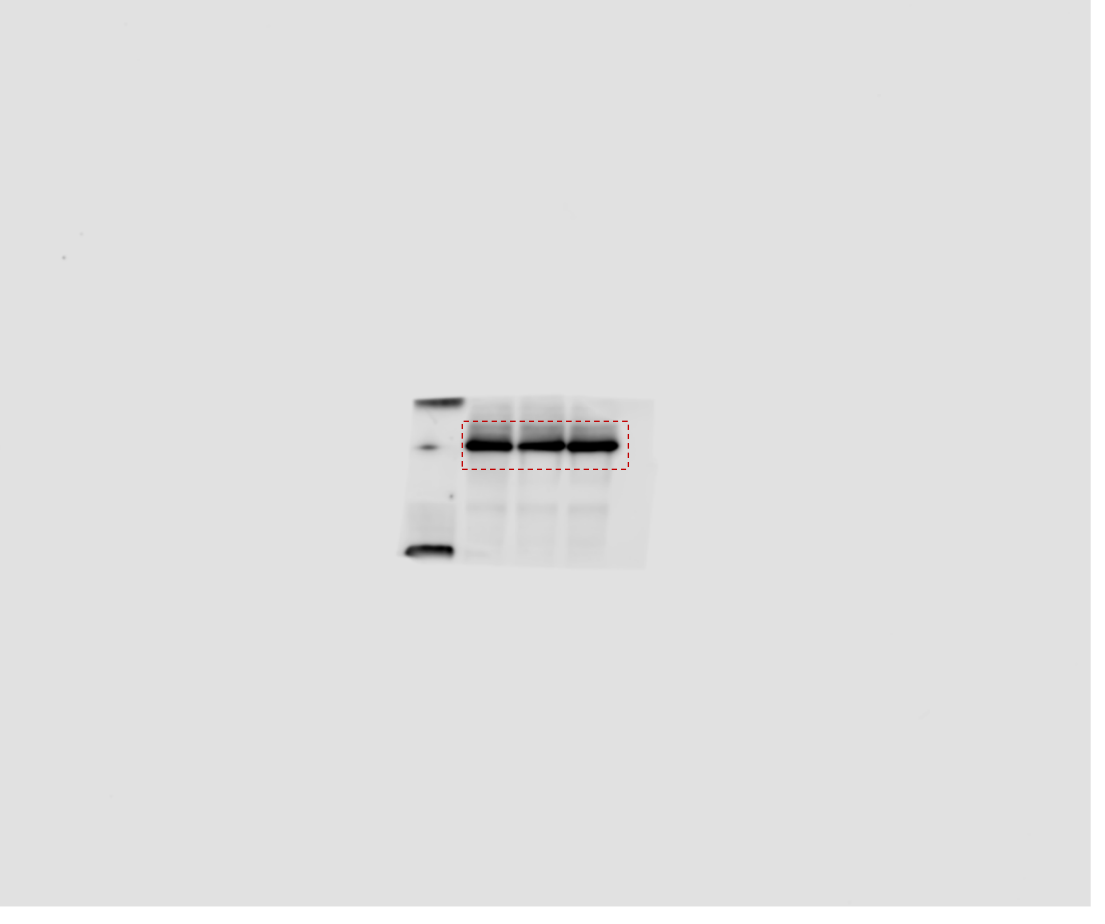

GAPDH Western Blot
(Figure 4E)

H1299 samples were loaded

(Bands shown on the figure are framed in red on the raw data)

LI-COR

Image ID: 0000526_01
Acquire Time: 18.Kas.2023 10:13:56

Acquisition Information

#	Image ID	Acquire Time	Channels	Integration Times	Analysis	Image Name	Comment	Image Modifications
1	0000526_01	18.Kas.2023 10:13:56	Chemi 700	05:30 00:30	Manual	0000526_01		

Image Display Values

Channel	Color	Minimum	Maximum	K
Chemi	Gray Scale (Black on White)	0,000659	0,0153	0
700	Gray Scale (Black on White)	0,0000502	0,0298	0

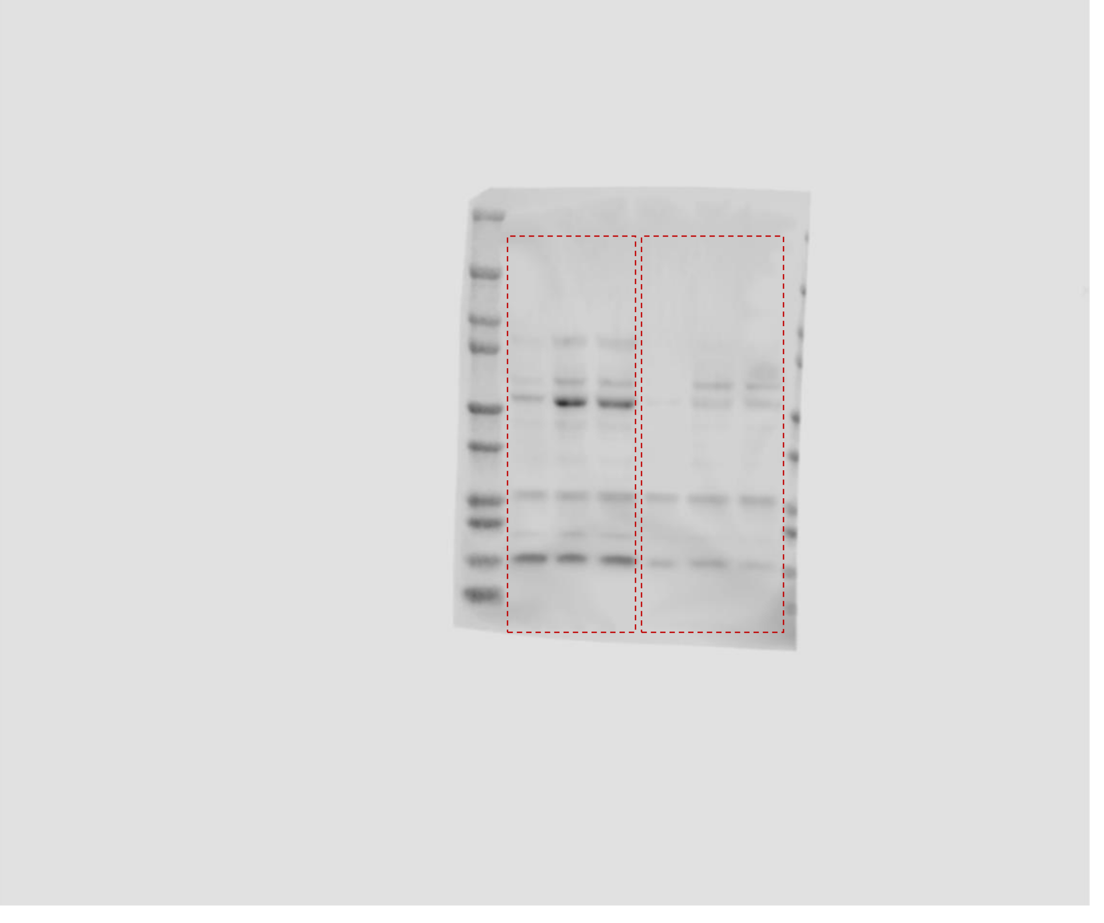

sDMA Western Blot
(Figure 4E)

Du145 and Panc1 samples were loaded sequentially

(Bands shown on the figure are framed in red on the raw data)

Acquisition Information

#	Image ID	Acquire Time	Channels	Integration Times	Analysis	Image Name	Comment
1	0002867_02	24.ub.2025 08:07:01	Chemi 600	02:00 00:30	Manual	0002867_02	240525 h1299 sdme 2min

Image Display Values

Channel	Color	Minimum	Maximum	K
Chemi	Gray Scale (Black on White)	0,00000727	0,101	0
600	Gray Scale (Black on White)	0,00177	0,916	0

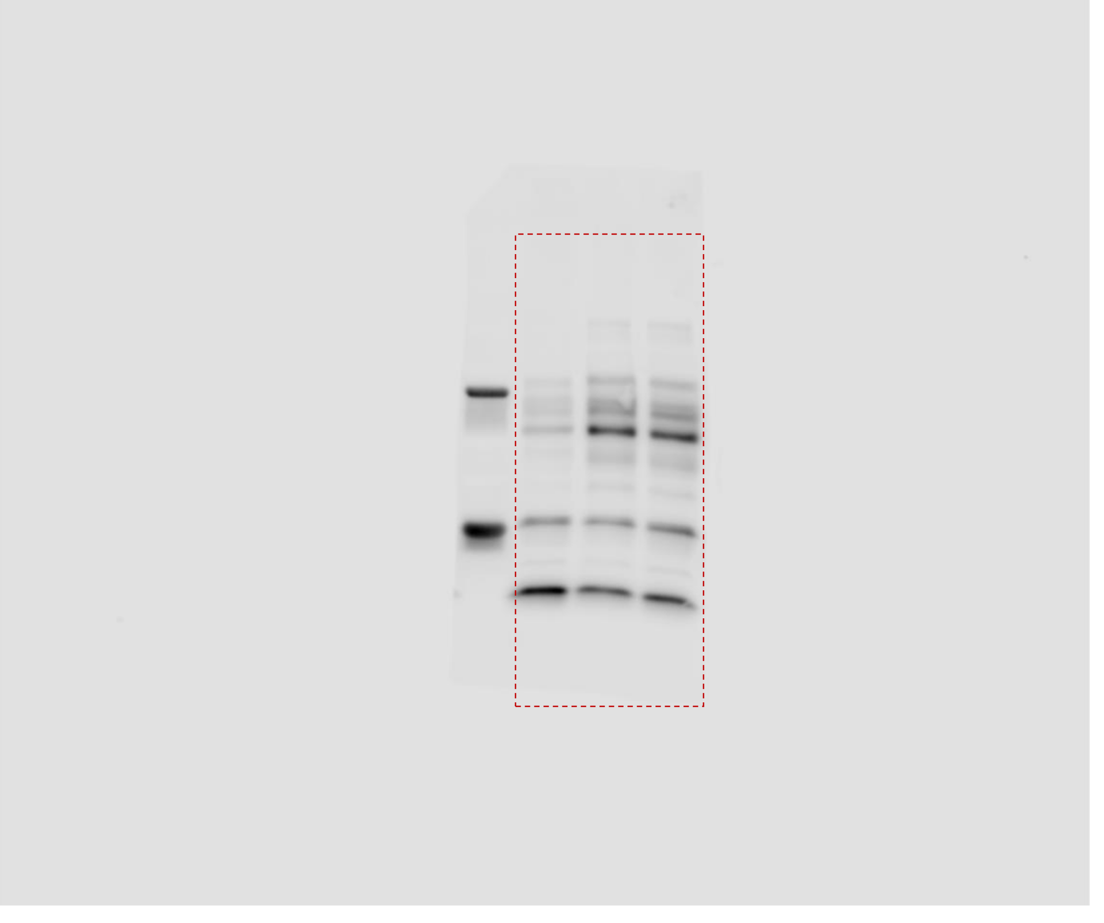

sDMA Western Blot
(Figure 4E)

H1299 samples were loaded

(Bands shown on the figure are framed in red on the raw data)

Image ID: 0000540_01
Acquire Time: 19.Kas.2023 10:02:40

Acquisition Information

#	Image ID	Acquire Time	Channels	Integration Times	Analysis	Image Name	Comment	Image Modifications
1	0000540_01	19.Kas.2023 10:02:40	Chemi 700	05:30 00:30	Manual	0000540_01		

Image Display Values

Channel	Color	Minimum	Maximum	K
Chemi	Gray Scale (Black on White)	0,000132	0,00301	0
700	Gray Scale (Black on White)	0,0000475	0,0363	0

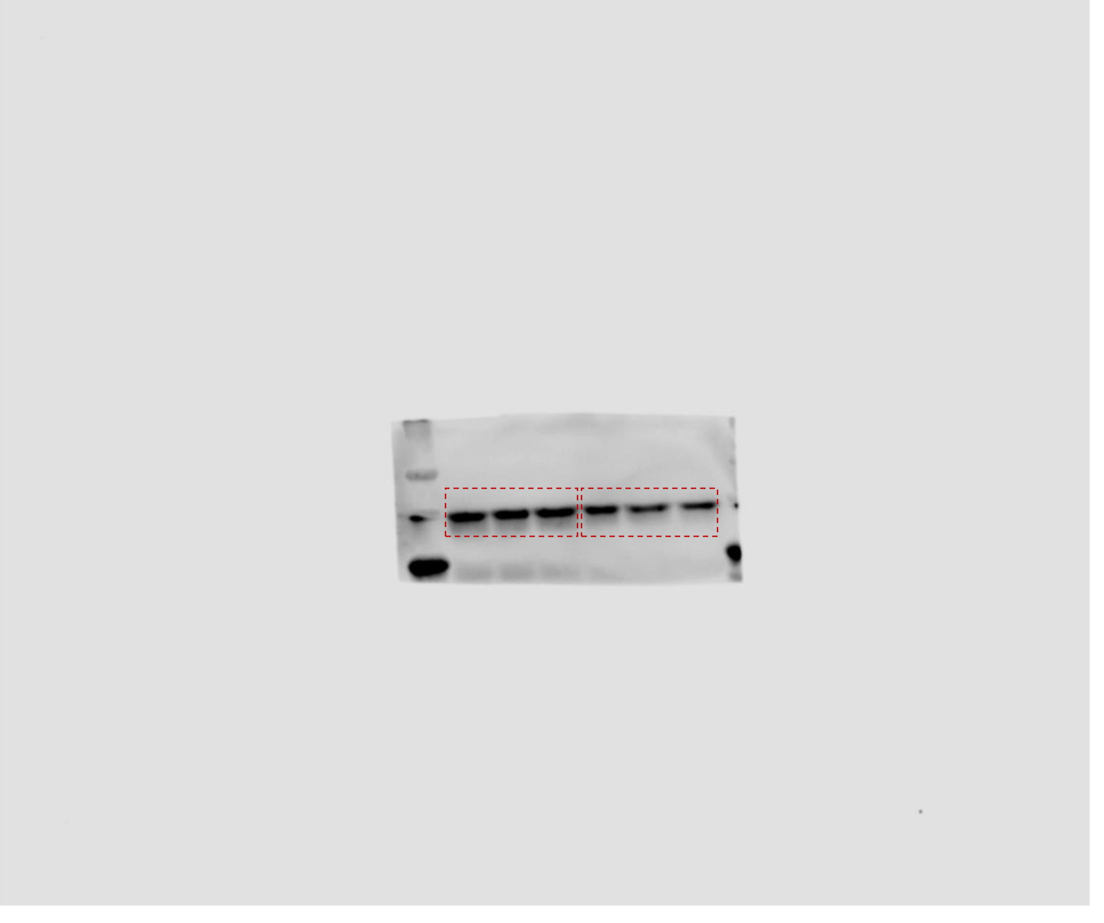

GAPDH Western Blot
(Figure 4E)

Du145 and Panc1 samples were loaded sequentially

(Bands shown on the figure are framed in red on the raw data)

Image ID: 0003015_02
Acquire Time: 07.Mar.2025 15:55:33

Acquisition Information

#	Image ID	Acquire Time	Channels	Integration Times	Analysis	Image Name	Comment
1	0003015_02	07.Mar.2025 15:55:33	Chemi 600	02:00 00:30	Manual	0003015_02	

Image Display Values

Channel	Color	Minimum	Maximum	K
Chemi	Gray Scale (Black on White)	0,000120	0,0154	0
600	Gray Scale (Black on White)	0,00141	0,900	0

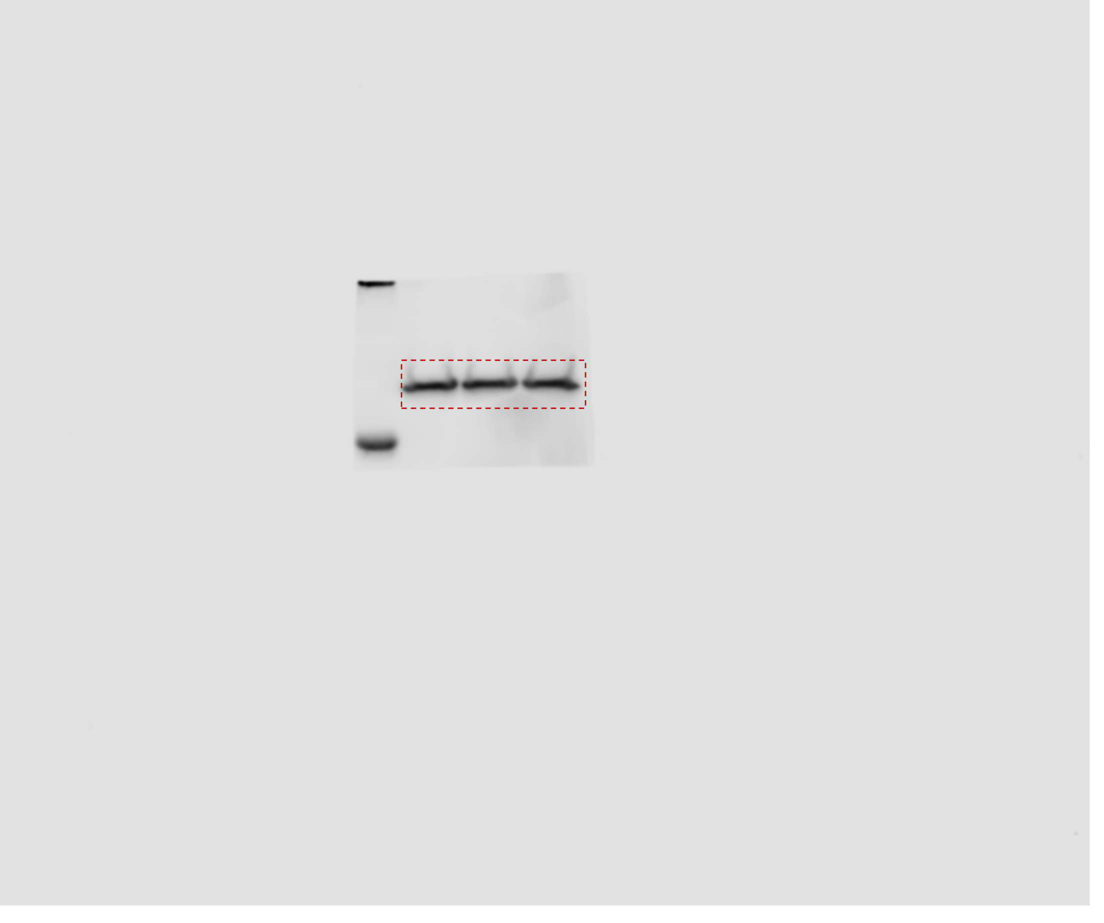

GAPDH Western Blot
(Figure 4E)

H1299 samples were loaded

(Bands shown on the figure are framed in red on the raw data)

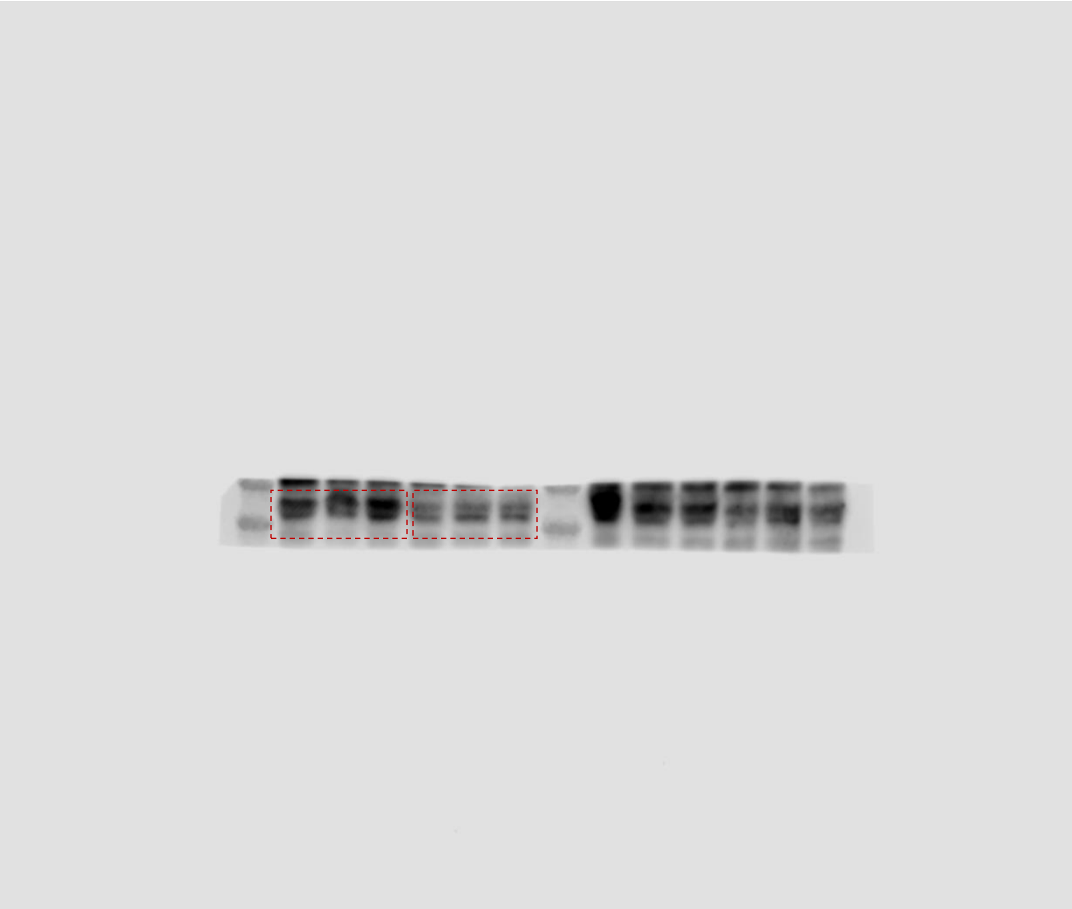

PRMT1 Western Blot
(Figure 4E)

Panc1 and Du145 samples were loaded sequentially
Two biological replicates were separated by protein ladder

(Bands shown on the figure are framed in red on the raw data)

Image ID: 0002871_02
Acquire Time: 24.ub.2025 08:26:18

Acquisition Information

#	Image ID	Acquire Time	Channels	Integration Times	Analysis	Image Name	Comment
1	0002871_02	24.ub.2025 08:26:18	Chemi 600	02:00 00:30	Manual	0002871_02	240225 h1299 PRMT1 2min

Image Display Values

Channel	Color	Minimum	Maximum	K
Chemi	Gray Scale (Black on White)	0,00000101	0,0152	0
600	Gray Scale (Black on White)	0,00159	6,00	0

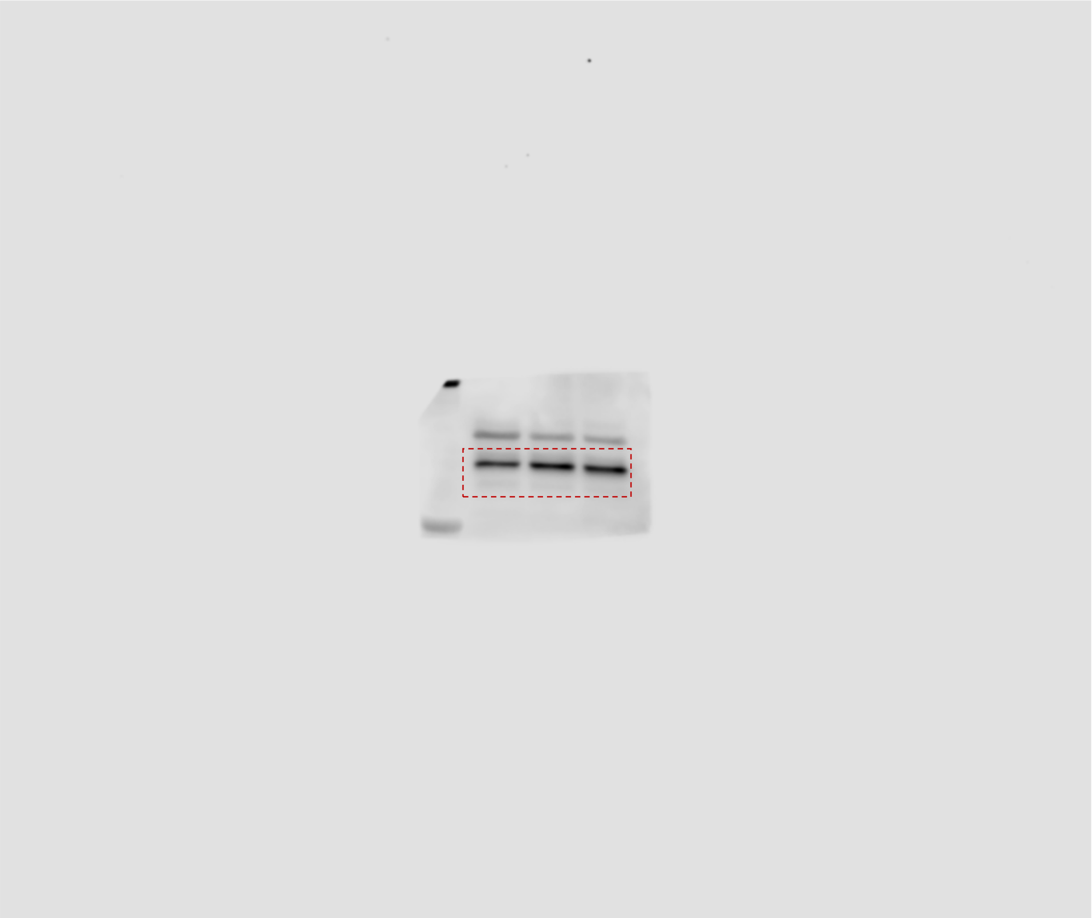

PRMT1 Western Blot
(Figure 4E)

H1299 samples were loaded

(Bands shown on the figure are framed in red on the raw data)

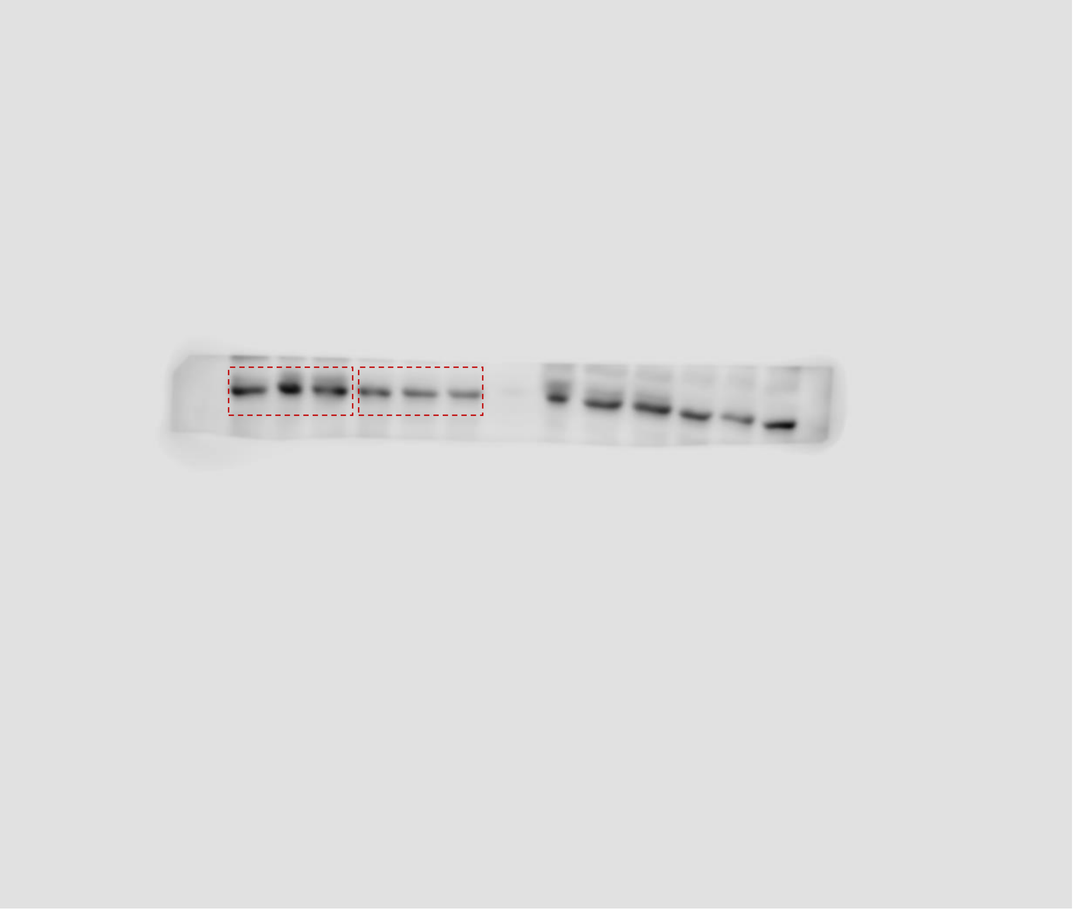

PRMT6 Western Blot
(Figure 4E)

Panc1 and Du145 samples were loaded sequentially
Two biological replicates were separated by protein ladder

(Bands shown on the figure are framed in red on the raw data)

LI-COR

Image ID: 0002872_01
Acquire Time: 24.ub.2025 08:31:27

Acquisition Information

#	Image ID	Acquire Time	Channels	Integration Times	Analysis	Image Name	Comment
1	0002872_01	24.ub.2025 08:31:27	Chemi 600	02:00 00:30	Manual	0002872_01	240225 h1299 prmt6 2min

Image Display Values

Channel	Color	Minimum	Maximum	K
Chemi	Gray Scale (Black on White)	0,000105	0,00653	0
600	Gray Scale (Black on White)	0,00155	1,37	0

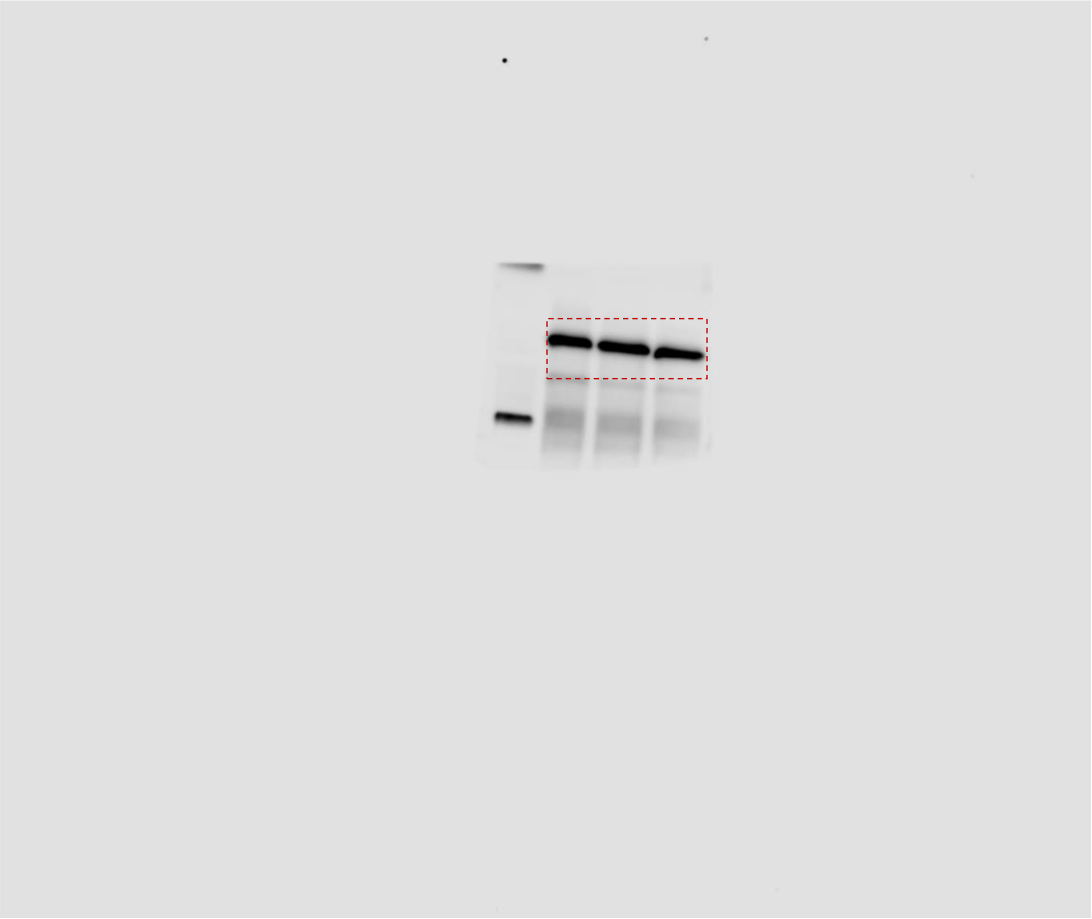

PRMT6 Western Blot
(Figure 4E)

H1299 samples were loaded

(Bands shown on the figure are framed in red on the raw data)

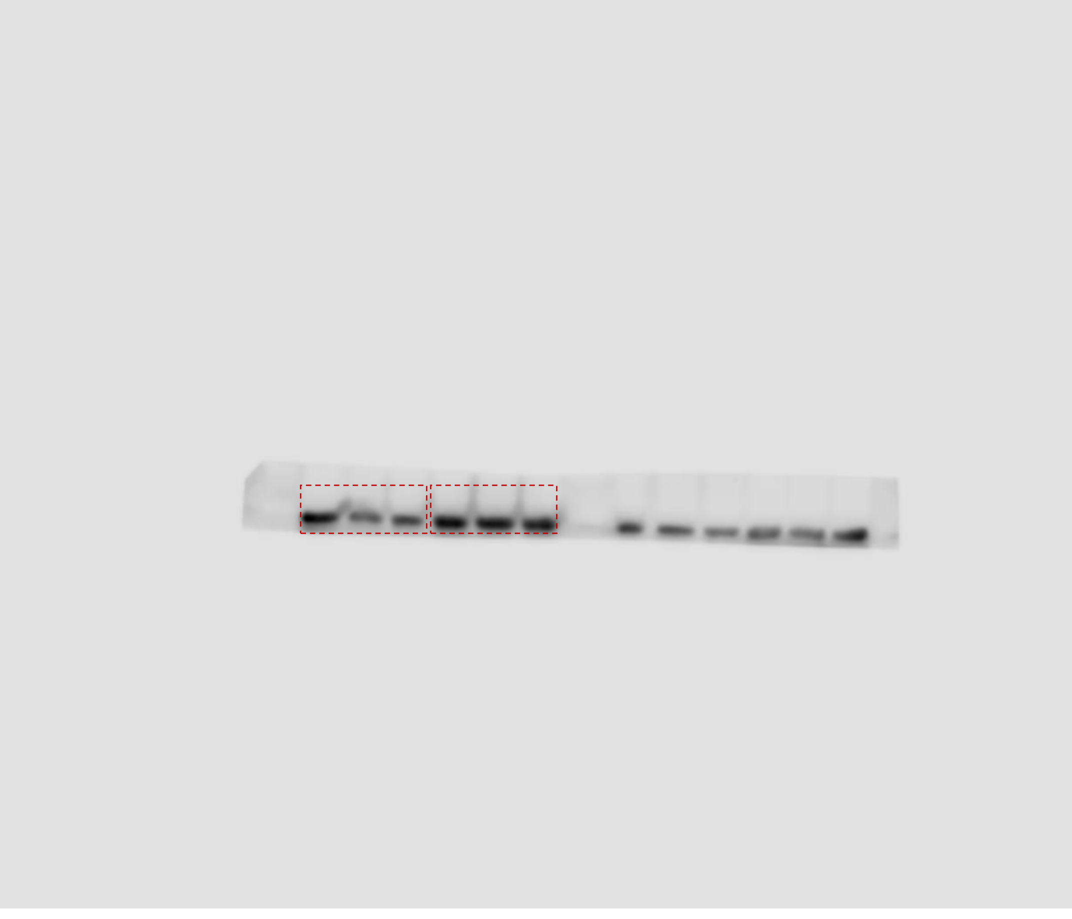

GAPDH Western Blot
(Figure 4E)

Panc1 and Du145 samples were loaded sequentially
Two biological replicates were separated by protein ladder

(Bands shown on the figure are framed in red on the raw data)

Image ID: 0002870_01
Acquire Time: 24.ub.2025 08:21:21

Acquisition Information

#	Image ID	Acquire Time	Channels	Integration Times	Analysis	Image Name	Comment
1	0002870_01	24.ub.2025 08:21:21	Chemi 600	02:00 00:30	Manual	0002870_01	240225 gapdhnfdm 2min

Image Display Values

Channel	Color	Minimum	Maximum	K
Chemi	Gray Scale (Black on White)	0,000140	0,0101	0
600	Gray Scale (Black on White)	0,00155	0,962	0

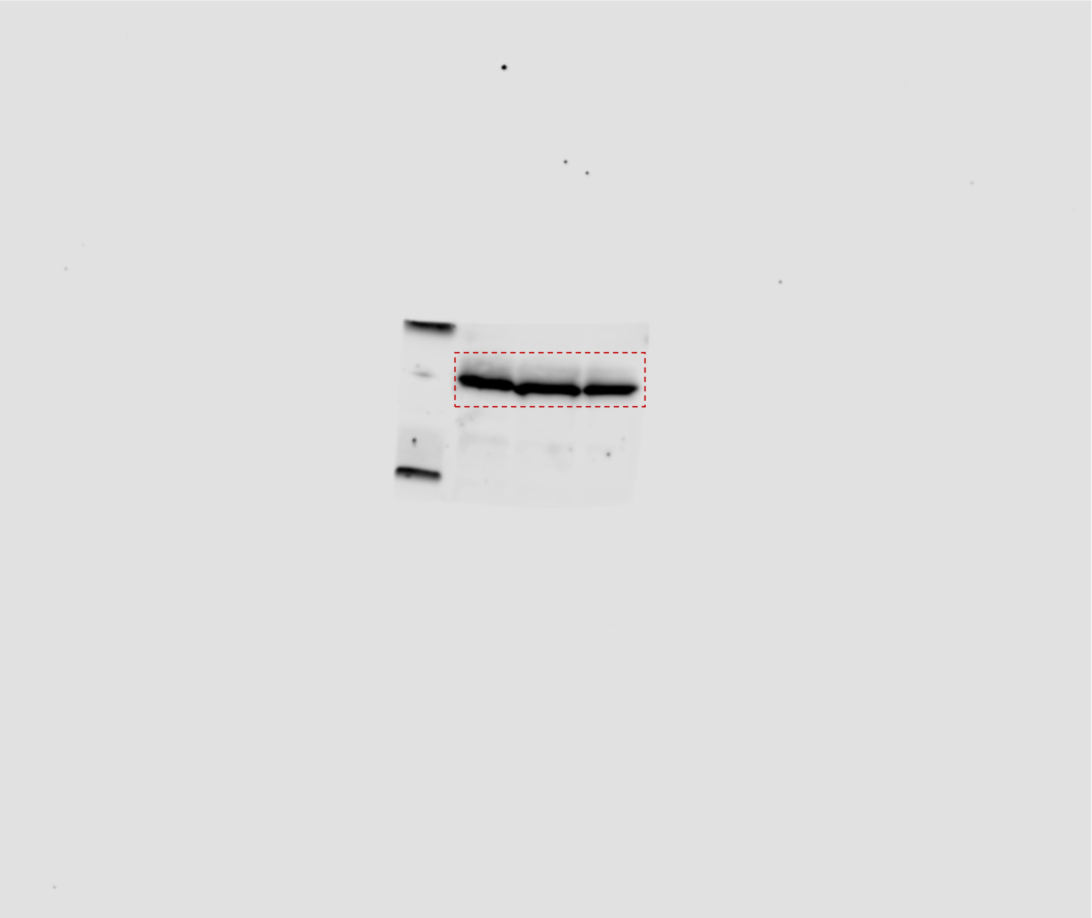

GAPDH Western Blot
(Figure 4E)

H1299 samples were loaded

(Bands shown on the figure are framed in red on the raw data)

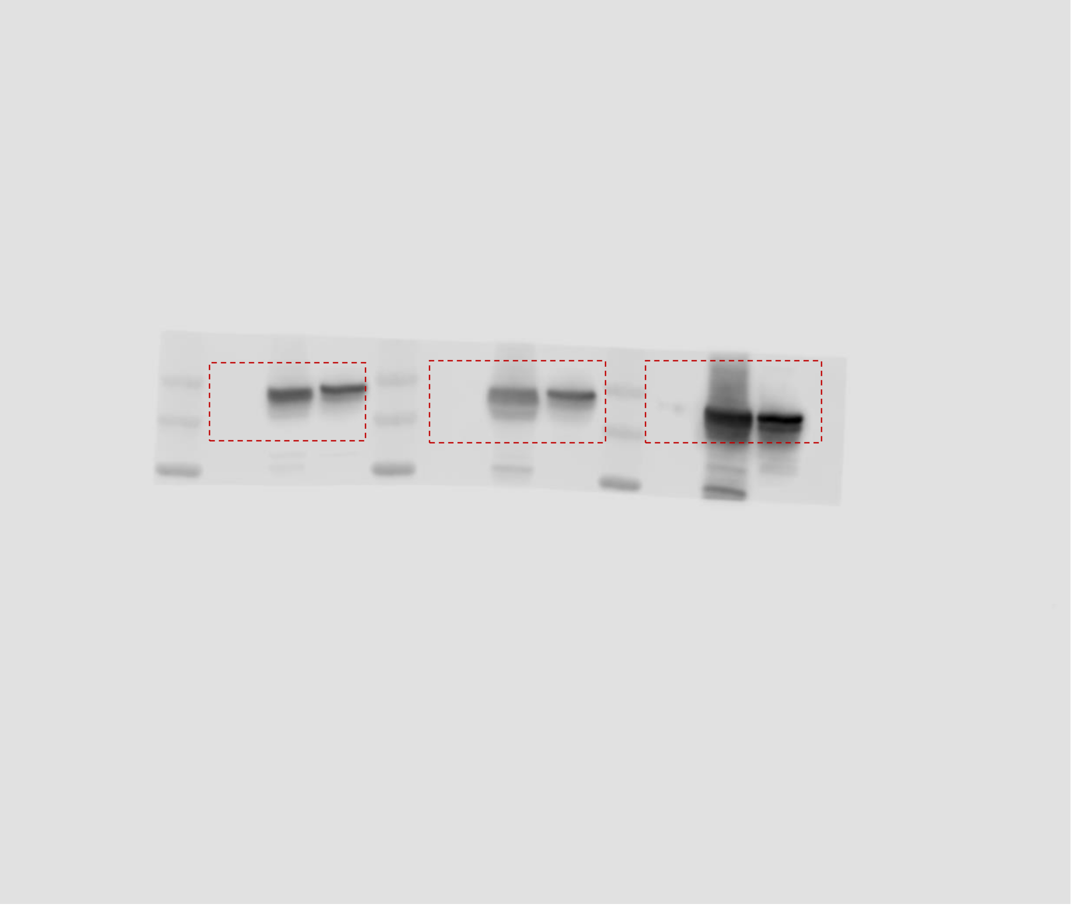

FLAG Western Blot
(Supplementary Figure 10)

Du145, Panc1, H1299 samples were loaded sequentially

(Bands shown on the figure are framed in red on the raw data)

Image ID: 0002267_01
Acquire Time: 17.Oca.2025 14:50:14

Acquisition Information

#	Image ID	Acquire Time	Channels	Integration Times	Analysis	Image Name	Comment	Image Modifications
1	0002267_01	17.Oca.2025 14:50:14	Chemi 700	02:00 00:30	Manual	170125_PRMT1		

Image Display Values

Channel	Color	Minimum	Maximum	K
Chemi	Gray Scale (Black on White)	0,0000378	0,0243	0
700	Gray Scale (Black on White)	0,000159	0,00866	0

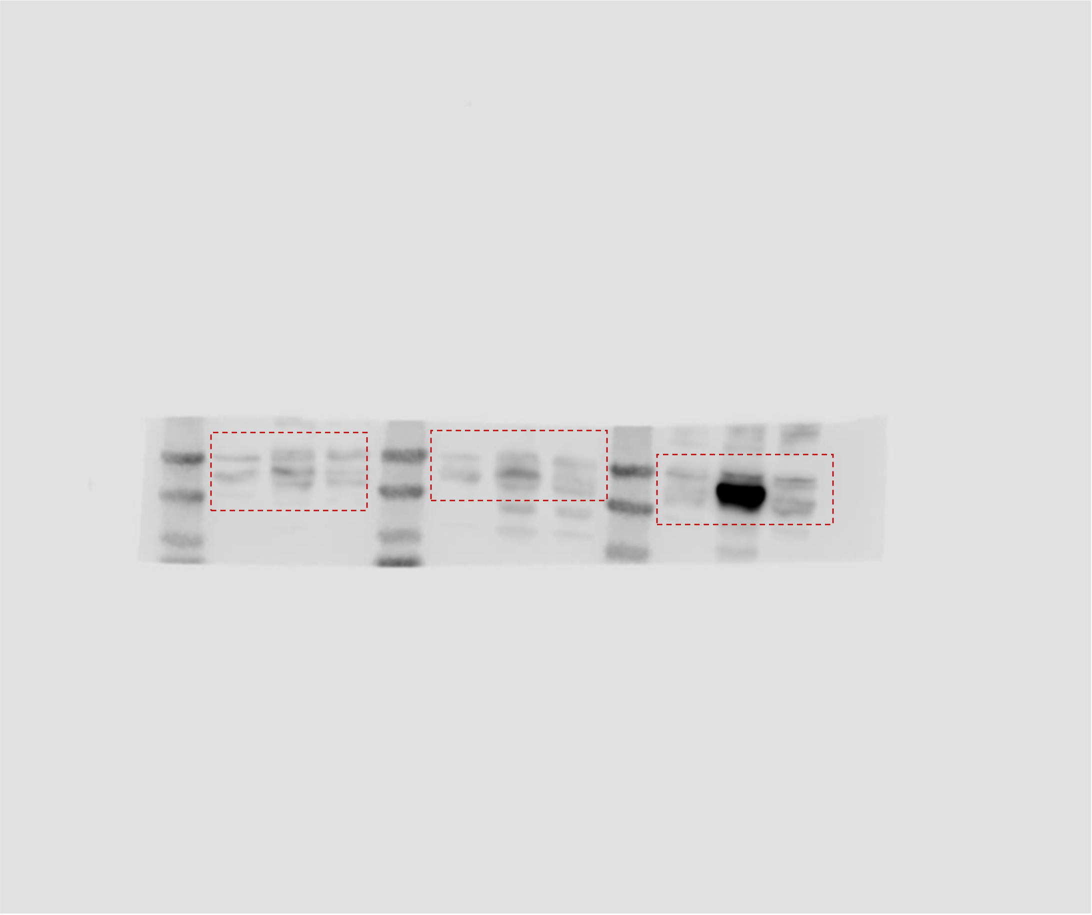

PRMT1 Western Blot
(Supplementary Figure 10)

Du145, Panc1, H1299 samples were loaded sequentially

(Bands shown on the figure are framed in red on the raw data)

Image ID: 0002268_01
Acquire Time: 17.Oca.2025 14:57:18

Acquisition Information

#	Image ID	Acquire Time	Channels	Integration Times	Analysis	Image Name	Comment	Image Modifications
1	0002268_01	17.Oca.2025 14:57:18	Chemi 700	02:00 00:30	Manual	170125_PRMT6		

Image Display Values

Channel	Color	Minimum	Maximum	K
Chemi	Gray Scale (Black on White)	0,0000000596	0,0193	0
700	Gray Scale (Black on White)	0,0000750	0,00842	0

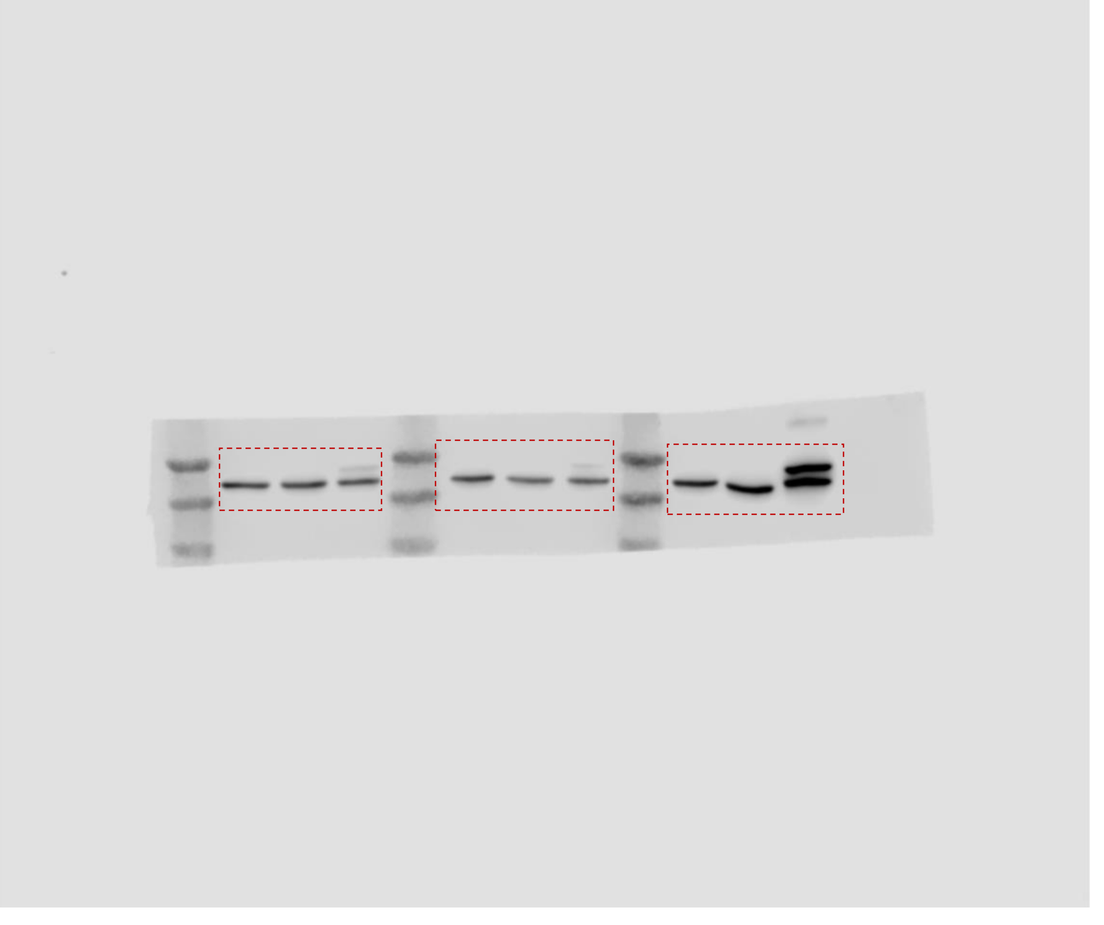

PRMT6 Western Blot
(Supplementary Figure 10)

Du145, Panc1, H1299 samples were loaded sequentially

(Bands shown on the figure are framed in red on the raw data)

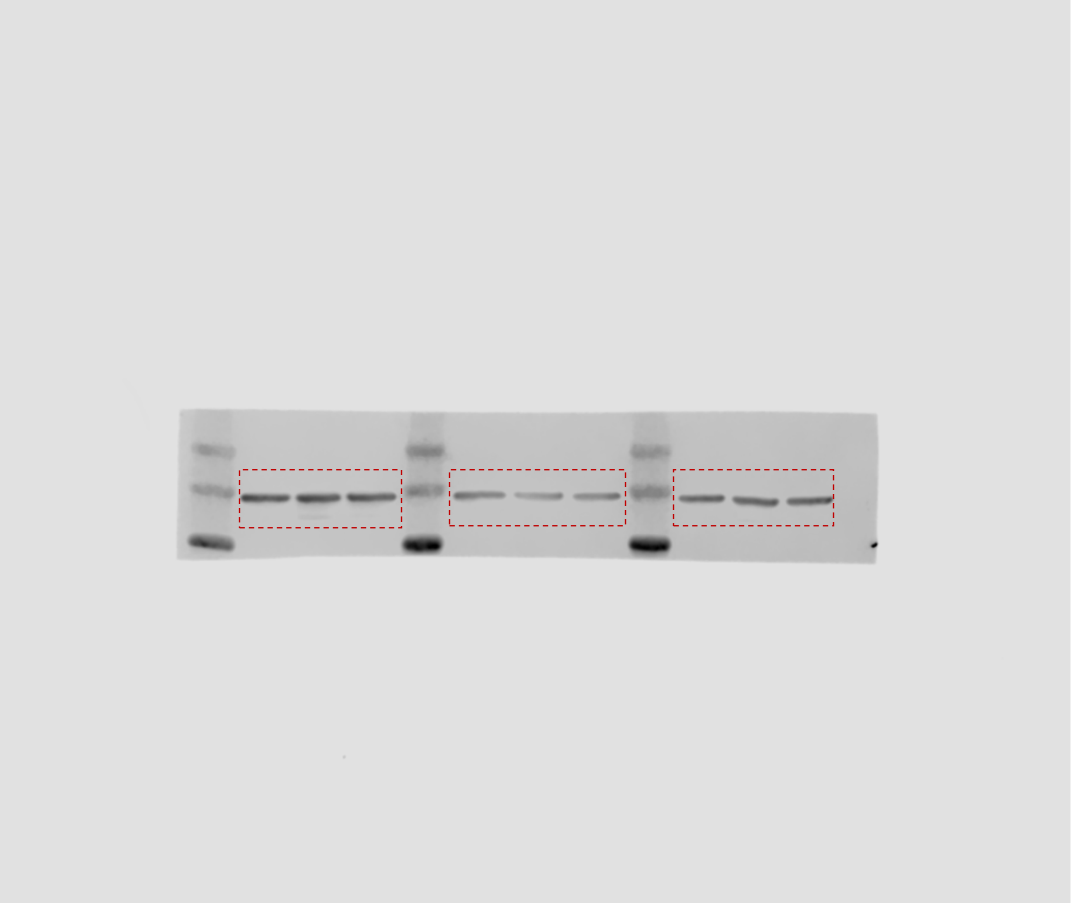

GAPDH Western Blot
(Supplementary Figure 10)

Du145, Panc1, H1299 samples were loaded sequentially

(Bands shown on the figure are framed in red on the raw data)
